# Virus induced gene editing using potyviral vectors in Cas12a expressing plants

**DOI:** 10.1093/hr/uhag017

**Published:** 2026-01-20

**Authors:** Fernando Merwaiss, Arcadio García, Ugo Rogo, Ivana Querol-Martí, Begoña García-Sogo, Carmine de Paola, Marta Rodriguez-Rodriguez, Benito Pineda, Vicente Moreno, Marta Vazquez-Vilar, Diego Orzáez, José-Antonio Daròs

**Affiliations:** Instituto de Biología Molecular y Celular de Plantas (Consejo Superior de Investigaciones Científicas-Universitat Politècnica de València), 46022 Valencia, Spain; Instituto de Biología Molecular y Celular de Plantas (Consejo Superior de Investigaciones Científicas-Universitat Politècnica de València), 46022 Valencia, Spain; Instituto de Biología Molecular y Celular de Plantas (Consejo Superior de Investigaciones Científicas-Universitat Politècnica de València), 46022 Valencia, Spain; Instituto de Biología Molecular y Celular de Plantas (Consejo Superior de Investigaciones Científicas-Universitat Politècnica de València), 46022 Valencia, Spain; Instituto de Biología Molecular y Celular de Plantas (Consejo Superior de Investigaciones Científicas-Universitat Politècnica de València), 46022 Valencia, Spain; Instituto de Biología Molecular y Celular de Plantas (Consejo Superior de Investigaciones Científicas-Universitat Politècnica de València), 46022 Valencia, Spain; Instituto de Biología Molecular y Celular de Plantas (Consejo Superior de Investigaciones Científicas-Universitat Politècnica de València), 46022 Valencia, Spain; Instituto de Biología Molecular y Celular de Plantas (Consejo Superior de Investigaciones Científicas-Universitat Politècnica de València), 46022 Valencia, Spain; Instituto de Biología Molecular y Celular de Plantas (Consejo Superior de Investigaciones Científicas-Universitat Politècnica de València), 46022 Valencia, Spain; Instituto de Biología Molecular y Celular de Plantas (Consejo Superior de Investigaciones Científicas-Universitat Politècnica de València), 46022 Valencia, Spain; Instituto de Biología Molecular y Celular de Plantas (Consejo Superior de Investigaciones Científicas-Universitat Politècnica de València), 46022 Valencia, Spain; Instituto de Biología Molecular y Celular de Plantas (Consejo Superior de Investigaciones Científicas-Universitat Politècnica de València), 46022 Valencia, Spain

## Abstract

Clustered regularly interspaced short palindromic repeat (CRISPR)-Cas systems are revolutionizing precision genome editing and gene expression control in crop plants. While effective CRISPR-Cas applications traditionally rely on labor-intensive stable genetic transformation to deliver Cas nucleases and guide RNAs into plant cells, plant viruses have emerged as a faster and efficient alternative, a strategy known as virus-induced gene editing (VIGE). Cas12a, Class 2 Type V CRISPR nucleases, are an alternative to broadly used Cas9 for plant genome engineering. Both kind of nucleases offer precise editing, but some Cas12a unique features make them particularly well suited for VIGE. In this study, we first used a tobacco rattle virus vector to compare editing efficiency of various target sequences and CRISPR RNA (crRNA) architectures in *Lachnospiraceae bacterium* ND2006 Cas12a (LbCas12a)-expressing *Nicotiana benthamiana* plants, evaluating results in infected tissues and seeds. Next, we developed a tobacco etch virus (genus *Potyvirus*)-derived vector efficiently delivering crRNAs throughout the plant. This approach enabled generation of plants with all four edited alleles in the allotetraploid *N. benthamiana* through *in vitro* regeneration from infected leaves, and to produce edited non-infected progeny, although at a very low frequency. We then demonstrated the successful application of the potyviral vector for VIGE in agronomically important crops, such as tomato or cultivated tobacco. Finally, we replicated this design using two other potyviral vectors, turnip mosaic virus, and lettuce mosaic virus. Given the conserved biological properties among potyviruses, we believe these findings are broadly applicable to the largest genus of plant RNA viruses, significantly expanding the host range of the VIGE technology.

## Introduction

Technologies derived from the clustered regularly interspaced short palindromic repeat (CRISPR) defensive systems from bacteria and archaea are revolutionizing all aspects of life sciences. Among these, agricultural biotechnology stands as a field with exceptional potential for advancement through CRISPR-derived applications [1, 2]. However, some aspects of plant biology, particularly the presence of a thick cell wall highly impermeable to large molecules pose some difficulties to the delivery of CRISPR reaction components. The most straight forward strategy to express CRISPR reaction components in plants is stable transformation. Although effective, this is a labor-intensive and time-consuming process that, in addition, cannot be generally applied to all crops and varieties. These characteristics make it difficult to use CRISPR technology in species that are recalcitrant to genetic transformation and regeneration through *in vitro* tissue culture. Alternative strategies to express the CRISPR reaction components in plant tissues are being intensively researched [3]. Among them, the use of viral vectors is gaining attention among plant biotechnologists based on simplicity and speed, and the promise of bypassing cumbersome tissue culture [4, 5].

Focusing on plant virus-derived vectors able to move systemically through the plant, the so-called virus-induced gene editing (VIGE) was first demonstrated in *Nicotiana benthamiana* transformed to constitutively express CRISPR-associated nuclease 9 from *Streptococcus pyogenes* (SpCas9) using a tobacco rattle virus (TRV) vector to express the single-guide RNA (sgRNA) [6]. CRISPR edits were even detected in the progeny, although at a very low rate. Heritability was substantially improved using an augmented sgRNA that incorporated a fragment of *Arabidopsis thaliana Flowering locus T* (*FT*) mRNA that was previously shown to favor RNA systemic movement, including the trafficking to the germline cells of the meristems, a tissue that is particularly surveilled to avoid viral infection [7–9]. While expression of sgRNAs in transgenic lines that stably express Cas nucleases is an important step forward, VIGE aims to co-express both the guide RNA and the Cas nuclease. This aim is particularly difficult due to cargo limitation of plant virus-derived vectors, although remarkable progress has been recently done using minus-strand RNA viruses, as well as intensively researched miniature Cas nucleases [10–12].

*Lachnospiraceae bacterium* ND2006 Cas12a (LbCas12a) is a Class 2 Type V CRISPR nuclease that has emerged as an alternative to the widely adopted SpCas9 for genome engineering. While both nucleases enable precise genome editing, LbCas12a possesses several distinct features that make it attractive for CRISPR applications [13–17]. Unlike SpCas9, which typically recognizes a guanine-rich PAM sequence, LbCas12a recognizes thymine-rich PAM sequences. This significantly expands the range of genomic sites that can be targeted, especially in adenine- and thymine-rich regions, such as promoters and introns, where SpCas9 might not be suitable. Moreover, LbCas12a has intrinsic endoribonuclease activity that allows it to process its own precursor into mature CRISPR RNA (crRNA). Finally, LbCas12a introduces staggered double-strand breaks in the target DNA with a 5′ overhang of four or five nucleotides, unlike the blunt ends produced by SpCas9. These staggered ends have been reported to favor the introduction of larger deletions as well as promoting gene insertion events by inducing homology-directed repair (HDR). It is noteworthy that, to date, few publications have described the implementation of VIGE in plant systems engineered to express Cas12a nucleases [10, 18].

Despite recent progress, VIGE must achieve substantial improvements to genuinely become a real alternative to stable plant transformation. A key challenge lies in host range: no universal viral vector exists. Each vector has a specific host range, and even with the same vector, CRISPR outcomes can vary drastically across different hosts, even within varieties of the same species, due to unique host–virus interactions. While various viral vectors have proven useful for CRISPR applications, the VIGE toolbox still needs to expand. Therefore, our aim in this work was to extend VIGE capabilities to vectors within the genus *Potyvirus*, the largest group of plant RNA viruses. Collectively, potyviruses infect a broad spectrum of host plant species, many of which are of significant agronomic interest [19].

In this study, we initially employed the well-established TRV VIGE vector to investigate critical aspects of genome editing in LbCas12a-expressing *N. benthamiana* plants. Specifically, we evaluated the efficiency of various crRNA sequences, the systemic spread of edits, and their heritability. Building on these findings, we then adapted the VIGE technology to utilize tobacco etch virus (TEV; genus *Potyvirus*) as a vector. Subsequently, we developed new tomato (*Solanum lycopersicum*) and cultivated tobacco (*N. tabacum*) lines constitutively expressing LbCas12a and successfully demonstrated the TEV vector functionality. Finally, we expanded VIGE to other potyvirus vectors, such as lettuce mosaic virus (LMV) and turnip mosaic virus (TuMV), highlighting the broad applicability of our findings.

## Results

### Optimization of a reporter system to evaluate VIGE using a TRV-derived vector

**Figure 1:**
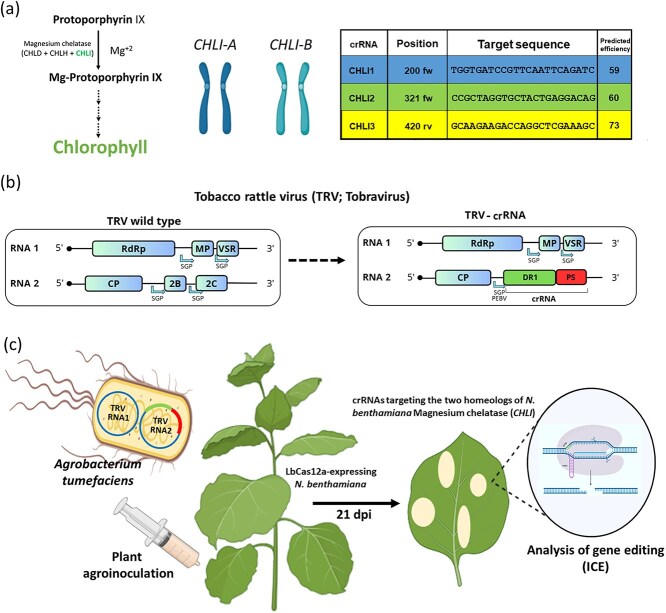
Schematic representation of the guide selection procedure and workflow followed to evaluate the CRISPR-Cas *CHLI* reporter system. (a) Metabolic pathway of chlorophyll biosynthesis, in which *CHLI* is involved. Three different crRNAs were selected that can simultaneously target both homeologs of *NbCHLI*, named here *CHLI-A* and *CHLI-B*. Target selection was based on the specificity scores given by CRISPOR tool. (b) The crRNA was formed by the LbCas12a direct repeat (DR1) scaffold, and the 23-nt protospacer (PS). The three selected crRNAs were cloned into a TRV vector, and their expression was driven by the PEBV promoter. (c) *N. benthamiana* plants were agroinoculated with the three constructs, and gene editing was analyzed at 21 dpi in systemic non-inoculated leaves displaying bleaching phenotype.

VIGE is gaining impact in plant biotechnology due to its great versatility and relative speed compared to more conventional techniques based on stable transformation. However, the properties of each viral vector are unique and the results of infections can vary even within cultivars in a single host species, meaning that not all viruses can be efficiently used to express guide RNAs in all host plants. The first objective of this work was to develop a robust VIGE system in *N. benthamiana* plants stably expressing the temperature-tolerant LbCas12a nuclease, as an alternative to SpCas9. To this end, we implemented a reporter system targeting the two homeologs of the *Magnesium chelatase subunit I* (*CHLI*), a component of an enzyme involved in chlorophyll biosynthesis (Fig. 1a, left), which is frequently used as a reporter gene in CRISPR-Cas assays, due to the particularly visible yellow or whitish phenotype conferred by its impairment [20]. To efficiently knockout *NbCHLI*, we targeted the two homeologous loci present in *N. benthamiana* genome, which we called for simplicity *CHLI-A* (located on chromosome 14) and *CHLI-B* (located on chromosome 12). Using the CRISPOR online tool [21], we designed three different crRNAs (named as crRNA-1, crRNA-2, and crRNA-3), which simultaneously targeted exon 3 of both homeologs (Fig. 1a, right, and Fig. S1). The crRNAs were composed of the LbCas12a direct repeat scaffold followed by the 23-nt protospacers CHLI1, CHLI2, and CHLI3. Protospacer selection was based on the specificity scores given by the CRISPOR tool, trying to avoid guides presenting off-targets at other genomic loci different from that targeted. To test *in vivo* the efficiency of the selected crRNAs, we cloned them into a TRV vector, well known to be functional in VIGE [22]. In this vector, crRNAs are expressed under the control of the pea early-browning virus (PEBV) promoter, and were cloned downstream the coat protein (CP) coding region, in the TRV RNA2 segment (Fig. 1b and Fig. S2).

**Figure 2:**
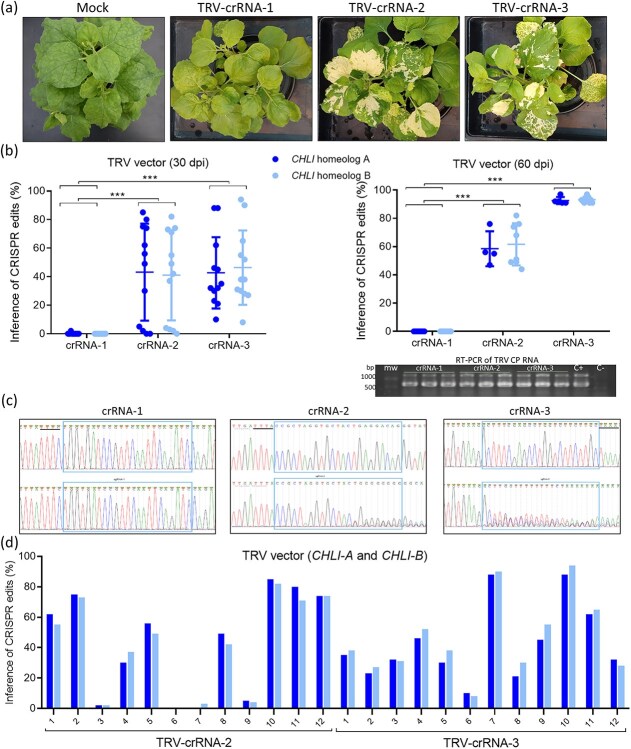
Comparison of the CRISPR editing efficiency for three different crRNAs against *NbCHLI*. (a) Pictures of representative plants agroinoculated with TRV vectors expressing the three selected crRNAs, as indicated. A control plant mock-inoculated is displayed on the left. (b) Comparison of the editing efficiency in both *NbCHLI* homeologs using the three selected crRNAs at 30 (left) and 60 dpi (right). Statistical analysis was performed using the GraphPad Prism 8 software. Data were analyzed by two-way ANOVA with Tukey's posttest (^***^P < 0.001). Electrophoresis gel shows the results of an RT-PCR analysis to detect the TRV CP RNA in all agroinoculated plants at 60 dpi. Mw, molecular weight marker; C+, positive control; C−, negative control. (c) Representative Sanger sequencing electropherograms at target sites of the three crRNAs analyzed. PAMs are highlighted by a black line. Electropherograms of control (unedited) plants are showed on top. (d) Analysis of the editing efficiency of TRV-crRNA-2 and -3 in the two *NbCHLI-A* (dark blue) and *NbCHLI-B* (light blue) homeologs from 12 individual samples per guide.

Viral constructs were agroinoculated in transgenic *N. benthamiana* plants expressing LbCas12a. Samples were taken from upper non inoculated leaves 21 days post-inoculation (dpi). Genomic DNA was purified, and the regions targeted by each crRNA were amplified by PCR, using primers specific of each homeolog. The Synthego interference of CRISPR edits (ICE) online tool was used to compare Sanger sequencing results with plants mock-inoculated, representing unedited control plants (Fig. 1c). Pictures of whole plants were taken at 30 dpi. The expected whitish phenotype in upper non-inoculated leaves was observed in plants inoculated with TRV-crRNA-2 and TRV-crRNA-3, but not in plants inoculated with TRV-crRNA-1 (Fig. 2a). Supporting these visual results, ICE analysis confirmed average indel percentages in systemic leaves of around 40% in both homeologs of plants inoculated with TRV-crRNA-2 and TRV-crRNA-3, while negligible genome editing was found in plants inoculated with TRV-crRNA-1. We wondered whether these results would be consistent along time, and we analyzed the same plants at 60 dpi. Again, both analyzed homeologs displayed very similar ICE percentages for each crRNA, with average results increased to around 60% and 90% for TRV-crRNA-2 and TRV-crRNA-3, respectively (Fig. 2b). In order to confirm that, in the case of TRV-crRNA-1, absence of editing was related with the target sequence and not with a problem with viral infection, RT-PCR analysis was applied to detect the presence of TRV in the different samples. TRV detection in all the analyzed samples supports the notion that crRNA-1 is not an efficient guide RNA for this technology (Fig. 2b). Representative sequencing electropherograms clearly showing gene editing in the expected target sites of crRNA-2 and crRNA-3 are shown in Fig. 2c. We realized that there was no significant difference between the average indel percentages obtained by analyzing the different homeologs separately, which suggests that the capacity of these two crRNAs to target both loci is the same. To deeper evaluate this idea, we individually plot the ICE values of both homeologs from 12 plants inoculated with each TRV-crRNA-2 and TRV-crRNA-3. Results showed comparable indel levels between both homeologs in all the samples, confirming that these crRNAs are equally functional against both genomic loci (Fig. 2d).

Because short RNAs, about the size of the 23-nt protospacers used in this work, expressed from a TRV vector have been recently shown to induce RNA silencing of the homologous plant genes [23], we asked about the contribution of *NbCHLI* silencing to our phenotypic observations. Wild-type and LbCas12a-expressing *N. benthamiana* plants were agroinoculated with TRV-crRNA-3. While wild-type plants showed a yellowing phenotype compatible with some degree of *NbCHLI* silencing at different times post-inoculation, LbCas12a-expressing plants exhibited intense white patches compatible with CRISPR-based know-out of the four *NbCHLI* alleles (Fig. S3a and b).

### Analysis of the crRNA architecture in the TRV vector

**Figure 3:**
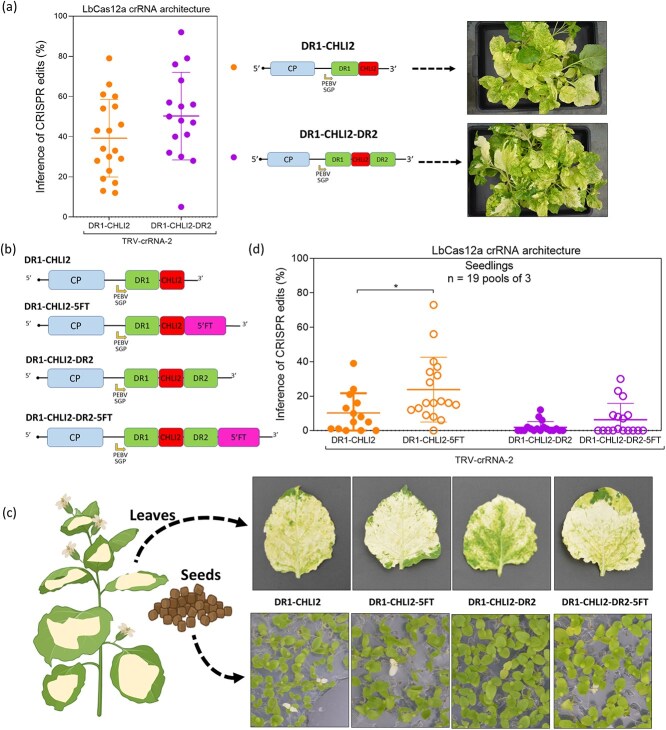
Heritable gene editing using the TRV vector. (a) A new crRNA-2 architecture with an additional direct repeat (DR2) located at the 3′ end of the protospacer (PS) was generated. Dot plot of the ICE percentages in tissues of *N. benthamiana* plants inoculated with TRV vectors expressing the different crRNA-2 architectures (DR1-CHLI2 and DR1-CHLI2-DR2), as indicated. Pictures of representative plants agroinoculated with TRV vectors expressing the two different architectures of crRNA-2 are displayed on the right. (b) Four different crRNAs architectures, differing on the DR flanking the protospacer (PS) and the presence or absence of the *A. thaliana Flowering locus T* (5FT) fragment*,* were expressed using the TRV vector (DR1-CHLI2, DR1-CHLI2-DR2, DR1-CHLI2-5FT, and DR1-CHLI2-DR2-5FT). (c) Leaves displaying white phenotype suggested the knockout of *NbCHLI* homologs in all cases (top). Seeds from infected plants were collected and sown *in vitro* in sterile Petri dishes supplemented with sucrose (bottom). (d) Seedlings coming from three different plants for each vector were grouped in pools of three and total DNA was extracted and analyzed. ICE values obtained for the different vectors are plotted.

To achieve heritable gene editing, we tested several crRNAs architectures. Based on previous studies supporting the use of two direct repeats flanking the 23-nt protospacer [13], we generated a modified TRV-crRNA-2, which also presented a second direct repeat located at the 3′ end of the crRNA (Fig. 3a and Fig. S2). The architecture of this new crRNA was named DR1-CHLI2-DR2. *Nicotiana benthamiana* LbCas12a plants were agroinoculated with TRV vectors expressing the crRNAs against *NbCHLI* with both architectures DR1-CHLI2 and DR1-CHLI2-DR2. Upper non-inoculated leaves displayed at 21 dpi, the expected bleaching phenotype in both cases (Fig. 3a). These visual symptoms correlated with the ICE percentages, in which values, although slightly higher for plants inoculated with the DR1-CHLI2-DR2 architecture, did not present statistically significant differences (Fig. 3a).

### Heritable gene editing using the TRV and the *CHLI* reporter system

Obtaining an edited progeny after plant selfing, and therefore avoiding tissue culture regeneration, is one of the main goals of the VIGE strategy. For this purpose, our next milestone was to induce editing of the germ line of agroinoculated plants. Different studies suggested that fusing mobile RNA motifs to guide RNAs can increase their mobility in plants, even reaching the germ line [7, 24]. To achieve this goal, we generated two new constructs based on the different TRV-crRNA-2 vectors (DR1-CHLI2 and DR1-CHLI2-DR2). We fused the 5′ fragment of the *Flowering locus T* (5FT) from *Arabidopsis thaliana* to the 3′ end of each crRNA, giving rise to two new architectures, named DR1-CHLI2-5FT and DR1-CHLI2-DR2-5FT (Fig. 3b and Fig. S2). These new constructs, along with the parentals with no 5FT, were agroinoculated in *N. benthamiana* LbCas12a-expressing plants. After 30 days, all agroinoculated plants displayed clear symptoms of *NbCHLI* inactivation in upper non-inoculated leaves (Fig. 3c, upper row). Seeds from different flowers of three different infected plants (for each construct) were collected. After sterilization, seeds were germinated *in vitro* on agar plates supplemented with sucrose. Completely white seedlings, indicating gene editing in the four alleles of the two homologs, were found in the progeny of plants inoculated with all the constructs but DR1-CHLI2-DR2 (Fig. 3c, lower row). We further analyzed the ICE percentages obtained in 57 seedlings for each construct grouped in pools of three, showing that best heritable editing was obtained when using a single direct repeat and a 5FT fragment flanking the protospacer (DR1-CHLI2-5FT) in the crRNA construct (Fig. 3d).

### Implementation of a potyvirus-derived vector for VIGE using LbCas12a

**Figure 4:**
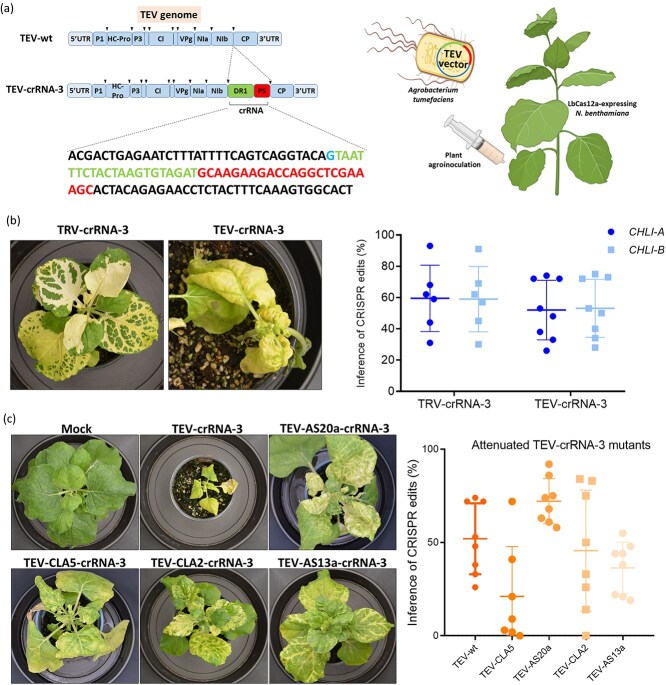
Developing a potyviral vector for crRNA delivery. (a) crRNA-3 was selected and cloned in between the NIb and CP cistrons of a TEV vector. A single direct repeat (DR1, green) located at 5′ of the protospacer (PS, red) was used. An additional G (blue) was added at the 5′ end of the crRNA-3 to maintain the viral open reading frame. *N. benthamiana* (LbCas12a) plants were agroinoculated and systemic tissue was analyzed at 21 dpi. (b) Both TRV and TEV vector infected plants presented the expected whitish phenotype, but TEV killed the plants after 30 dpi. When analyzing ICE scores, both homeologs resulted edited at similar proportions using the two viral vectors. (c) Plants were inoculated with four attenuated versions of TEV (AS20a, CLA2, CLA5, and AS13a) and a considerable symptom reduction was observed (left). ICE was analyzed in the four different mutant viral vectors and compared to that from the unmodified TEV-crRNA-3 vector (right).

Extending the VIGE technology to potyviruses is of great interest due to the large number of species in the genus and a broad collective host range. Although presenting a big cargo capacity, the ability to express heterologous sequences is restricted due to their replication strategy, which relies on the expression of large polyproteins. Thus, when expressing exogenous sequences within a potyviral vector, it is essential to keep the reading frame and not to introduce any stop codon in the coding sequence, which would impair infectivity. To evaluate this, we selected the crRNA-3 instead of the crRNA-2 because the former had no stop codons in its sequence. The crRNA-3 was then cloned in between the nuclear inclusion b (NIb) and the CP cistrons of a vector derived from TEV (Fig. 4a and Fig. S4). *Nicotiana benthamiana* (LbCas12a) plants were agroinoculated, and ICE was evaluated as explained above. A TRV vector expressing the same crRNA-3 was used as a control. At 21 dpi, plants showed the expected whitish phenotypes, and both homeologs of the *NbCHLI* gene were edited in the same proportion when guide RNAs were delivered by TRV or TEV vectors (Fig. 4b). However, TEV-infected plants presented necrotic symptoms and died after 30 dpi (Fig. 4c). To overcome this situation, we introduced four single-point mutations in the TEV HC-Pro cistron, which can attenuate symptom development in infected plants [25]. The four mutations are named AS20a, CLA2, CLA5, and AS13a. When inoculated with these mutated viral vectors, plants showed a better morphological development compared to the wild-type version of our TEV-crRNA-3. However, only CLA2 and AS13a allowed plant survival and production of functional flowers. Genome editing evaluation in upper non-inoculated tissues, showed comparable results in plants inoculated with the unmodified TEV-crRNA-3 and the CLA2 and AS13a mutant vectors. Average indel percentage decreased in plants inoculated with the CLA5 mutant, and was higher in plants inoculated with the AS20a mutant (Fig. 4c). Symptoms on wild-type and LbCas12a-expressing *N. benthamiana* plants infected with TEV-CLA2-crRNA-3 were compared, with similar results to those obtained previously with TRV-crRNA-3 (Fig. S3c).

**Figure 5:**
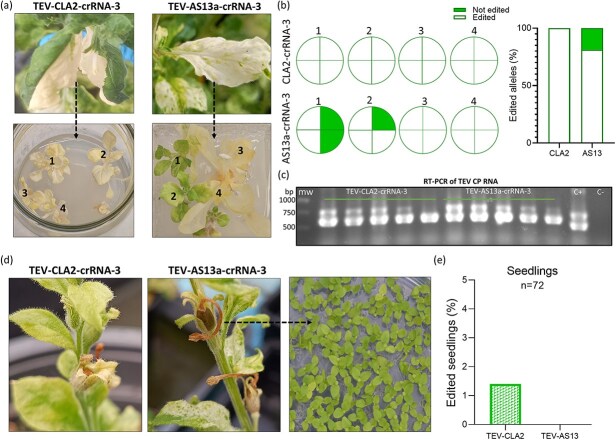
*In vitro* regeneration of edited plants from TEV-infected tissue and heritable gene editing. (a) Plant leaves displaying white patches were collected and served as explants for *in vitro* regeneration. Complete white plants were obtained after regeneration from plants infected with TEV attenuated variants TEV-CLA2-crRNA-3 and TEV-AS13a-crRNA-3. (b) ICE analysis was conducted on four plants from each condition. Results showed that most of the plants were completely edited. The proportion of edited (white) and non-edited (green) alleles is displayed for individual plants (left) and in the complete population (right). (c) Gel electrophoresis showing the results of a RT-PCR to detect the TEV CP RNA in regenerated plants. mw, molecular weight marker; C+, positive control; C−, negative control. (d) Seeds from plants infected with TEV-CLA2-crRNA-3 and TEV-AS13a-crRNA-3 were harvested and sown in sterile Petri dishes supplemented with sucrose. (e) The presence of edited alleles in both homeologs was evaluated by ICE in grown seedlings derived from mother plants infected either with TEV-CLA2-crRNA-3 or TEV-AS13a-crRNA-3.

In order to obtain homozygous mutants, we decided to regenerate complete plants from infected tissues. At 60 dpi, tissues from plants inoculated with TEV-CLA2-crRNA-3 and TEV-AS13a-crRNA-3 presenting white patches were surface sterilized and used for tissue culture regeneration. Several fully white plants were obtained. Light green plants were also obtained in the case of the AS13a mutant vector (Fig. 5a). ICE analysis of both *NbCHLI* homeologs of four white plants regenerated from a TEV-CLA2-crRNA3-infected plant showed that all the alleles were edited, supporting complete inactivation of the reporter gene, and thus explaining the white phenotype. In the case of white plants regenerated from a TEV-AS13a-crRNA3-infected plant, the same outcome was observed, while light green plants presented only two or three edited alleles (Fig. 5b). The presence of TEV was confirmed in all regenerated plants by RT-PCR (Fig. 5c).

Despite our constructs did not contain mobile signals to potentially traffic to the germline cells, we finally evaluated the heritability of the genome edits, which were caused from attenuated TEV vectors (TEV-CLA2-crRNA-3 and TEV-AS13a-crRNA-3). Seeds from infected plants were collected from different flower buds close to white tissues, and they were germinated *in vitro* (72 seedlings per construct) (Fig. 5d). ICE analysis of the obtained progeny revealed that one of the seedlings from TEV-CLA2-crRNA-3-infected plants presented an edited allele. However, no edited seedlings were found in the case of TEV-AS13a-crRNA-3-infected plants progeny (Fig. 5e). Notably, TEV was not detected by RT-PCR in the seedling that harbor the edited allele. Taken together, these results suggest that the TEV attenuated vector (TEV-CLA2) can be implemented for VIGE, and edited plants can be obtained after tissue culture regenerations, as well as after plant selfing, although in this last case at a very low rate.

### VIGE using a potyviral vector in species of agronomical interest

Encouraged by the validation of our TEV-based VIGE system in the model plant *N. benthamiana*, we set out to verify the transferability of the results to plants of agronomic interest, such as cultivated tobacco and tomato. To do so, the first step was to obtain transgenic *N. tabacum* (cv. Ottawa) and *S. lycopersicum* (cv. Moneymaker) plants that expressed the LbCas12a nuclease. We first designed a construct (pLBHig-Cas12att; Fig. S5) to transform the plants to stably express a temperature-tolerant version of LbCas12a, harboring the single mutation D156R [26, 27]. The expression of the Cas nuclease was driven by the cauliflower mosaic virus (CaMV) 35S promoter and terminator. Plants regenerated in selective media were tested by PCR to verify the presence of the T-DNA. Positive plants were selfed to generate the lines that were later used in the analyses.

**Figure 6:**
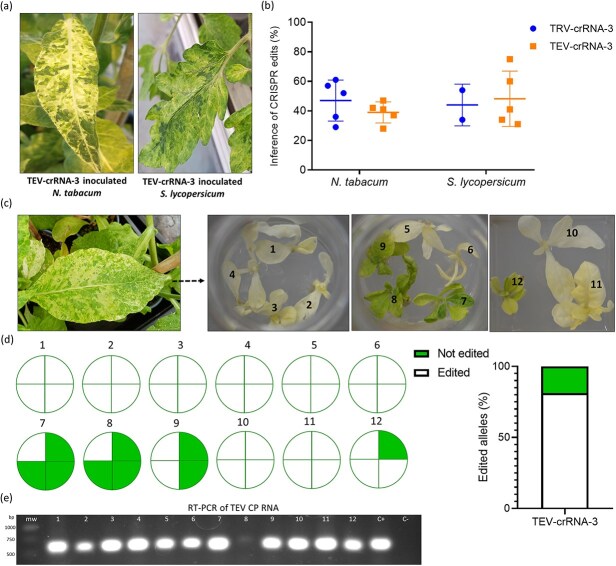
Implementation of the VIGE approach in species of agronomical interest. (a) *N. tabacum* and *S. lycopersicum* LbCas12a-expressing plants agroinoculated with TEV vectors displayed whitish and yellowish phenotypes at 30 dpi. (b) Both TRV and TEV vectors were able to induce similar CRISPR editing in inoculated plants. (c) As performed with *N. benthamiana*, *N. tabacum* plant leaves displaying white patches were collected and served as explants for *in vitro* regeneration (left). Complete white shoots, and light green plants were regenerated from *N. tabacum* plants infected with TEV-crRNA-3. (d) ICE analysis was conducted on eight fully white plants, and four light green plants. The proportion of edited (white) and non-edited (green) alleles in individual plants (left) and in the complete population (right) is presented. (e) Electrophoresis gel showing the results of a RT-PCR to detect the TEV CP RNA in regenerated plants. mw, molecular weight marker; C+ and C−, positive and negative controls, respectively.

Transformed tobacco and tomato plants expressing LbCas12a nuclease were inoculated with both TRV and TEV vectors expressing crRNA-3. The 23-nt sequence targeted by these vectors is conserved in cultivated tobacco (*NtCHLI*) homeologs, while presenting a single mismatch at position 21 in the case of tomato (*SlCHLI*). At 30 dpi plants displayed the expected bleaching phenotype in upper non inoculated leaves, especially in cultivated tobacco (Fig. 6a). Samples were taken from these tissues, and ICE analysis was performed. The results showed a substantial gene editing in systemic tissues of both plants, with a comparable outcome when using the TRV or TEV vectors (Fig. 6b).

Aiming to obtain *N. tabacum* homozygous mutant plants, we repeated the same approach used with edited *N. benthamiana* plants. Adult plants were regenerated by tissue culture techniques from infected tissues collected 2 months post-agroinoculation. Gene editing in *NtCHLI* homeologs was verified in the explant leaf sources, prior to plant regeneration. As in the case of *N. benthamiana*, several fully white plants were obtained, suggesting a complete inactivation of both *NtCHLI* homeologs. Similarly, light green plants were also obtained after regeneration (Fig. 6c). ICE analysis of eight different fully white plants proved the presence of CRISPR edits in all the four alleles of *NtCHLI* (Fig. 6d). Light green plants presented either one, two, or three edited alleles. This showed that the presence of at least one allele, coding for a functional protein, leads to a chlorophyll level visible to the naked eye in *N. tabacum*. Considering the total amount of alleles analyzed, more than 80% of them were classified as edited. Notably, TEV diagnosis in the regenerated plants by RT-PCR showed that one of the plants, carrying one edited allele, was apparently free of the virus (Fig. 6e).

### Transferring the TEV-based VIGE approach to other potyvirus species

**Figure 7:**
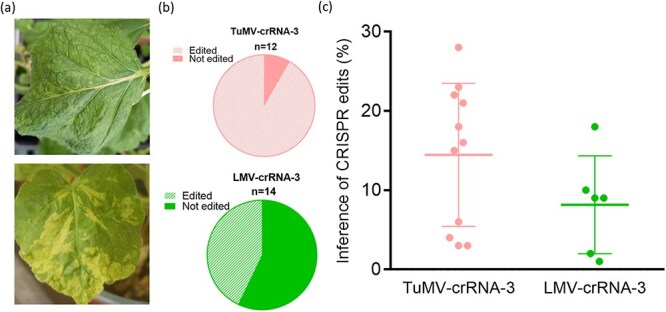
Transferring the VIGE approach to other potyviral systems. (a) LbCas12a-expressing *N. benthamiana* plants were agroinoculated with TuMV (top) and LMV (bottom) vectors expressing crRNA-3, and plants were analyzed at 30 dpi. (b) Pie charts represent the proportion of samples that presented detectable editing in the *NbCHLI-A* homeolog according to the ICE analysis. (c) Plot of ICE percentages from positive edited samples.

The positive results obtained when expressing crRNAs using the TEV vector, led us to evaluate the possibility of using other potyviral vectors with the same purposes. This would increase the number of hosts that would benefit from our VIGE strategy. We therefore developed two vectors, derived from TuMV and LMV, which expressed crRNA-3 in the same position as previously described for the TEV vector (Fig. S4). In the case of TuMV, we used the JPN1 sequence variant [28] that induces mild symptoms in *N. benthamiana*. The LMV sequence variant used [29] also induces mild symptoms in *N. benthamiana*. LbCas12a-expressing *N. benthamiana* plants were agroinoculated with the two viral vectors, and plants were analyzed at 30 dpi. Upper non inoculated leaves displayed infection symptoms for both vectors (Fig. 7a). ICE analysis supported this visual symptom evaluation. When guides were delivered by TuMV, 11 out of 12 analyzed samples were positively edited, while for LMV, close to 50% of analyzed samples presented genome editing in the target alleles (Fig. 7b). Positively edited samples were plotted individually based on their ICE value, showing a higher editing efficiency of TuMV compared to LMV (Fig. 7c). These results indicate that vectors based in different potyvirus species can be extensively applied for VIGE following the strategy here developed.

## Discussion

In recent years, we have witnessed the emergence of CRISPR-Cas systems as highly effective platforms for precision genome editing and targeted control of gene expression. This technology holds significant appeal for crop science, offering the potential to develop new cultivars that are more productive, nutritious, resistant to pests and pathogens, and better adapted to evolving environmental conditions. However, plant genome editing has traditionally depended on stable genetic transformation and *in vitro* tissue culture regeneration. This lengthy and complex process involves *A. tumefaciens*–mediated genetic transformation to express CRISPR-Cas reaction components, followed by *in vitro* culture to regenerate whole plants. Consequently, the genomes of many plant species are difficult to edit due to low transformation efficiency or poor *in vitro* regeneration [4]. To overcome this limitation, VIGE has emerged as a faster innovative alternative, which also includes in some modalities a tissue culture-free process. VIGE utilizes plant viruses natural ability to replicate and spread systemically, employing them as vectors to deliver CRISPR-Cas reaction components directly to adult plants, ideally reaching the germline cells for heritable genome editing [30, 31].

Most VIGE-based research to date has utilized plants that express the SpCas9 nuclease with excellent results [6, 7, 32]. However, Cas12a nucleases possess some features that could facilitate genome editing in some instances [13–17]. Unlike Cas9, Cas12a nucleases recognize thymine-rich PAM sequences (5′-TTTN-3′ in the case of LbCas12a), thereby expanding the range of targetable genomic sites, especially within adenine- and thymine-rich regions, like promoters and introns. Cas12a nucleases possess intrinsic endoribonuclease activity, allowing them to process their own crRNAs without the need for tracrRNA, which streamlines gene editing tool design and facilitates multiplex editing. Furthermore, Cas12a nucleases produce staggered double-stranded breaks with 5′ overhangs, which have been reported to favor gene insertion events and potentially promote HDR. In addition, the thermotolerant LbCas12a engineered variant offers improved efficiency at typical plant growing temperatures [24]. Furthermore, a side-by-side comparison of LbCas12a and SpCas9 nucleases in eight *N. benthamiana* overlapping loci using transient expression showed that, although both nucleases exhibit drastic target-dependent differences in efficiency, LbCas12a produced higher mutagenesis rates in five of the eight loci assayed, and displayed the highest editing average [13]. TRV has become one of the most widely used viral vectors for genome editing and its ability to systemically spread through plant tissues has been used as an effective tool for delivering sgRNAs into transgenic plants that express a SpCas9 nuclease [7, 9]. The first objective of our work was to develop a robust VIGE system in *N. benthamiana* plants stably expressing the LbCas12a nuclease. Various studies have demonstrated TRV effectiveness in inducing visible somatic gene edits by delivering sgRNAs targeting the *CHLI* gene. This gene is involved in the biosynthesis of chlorophyll, serving as an effective visual marker [33]. In particular, *N. benthamiana* possesses two homeologous genes that encode this subunit, named in this work *CHLI-A* and *CHLI-B*. The expression of both must be interrupted to observe a visually detectable white phenotype [34]. Three distinct crRNAs were computationally designed using the CRISPOR online tool, aiming for simultaneous targeting of exon 3 in both homeologous genes (Fig. 1). Each crRNA was structured with the LbCas12a direct repeat scaffold preceding a 23-nt protospacer. Protospacer selection was rigorously guided by specificity scores provided by the CRISPOR tool, with a strong emphasis on avoiding off-target interactions at non-target genomic loci. Indel percentages, quantified via ICE analysis in systemic leaves, demonstrated ~40% editing efficiency in both homeologs following inoculation with viruses expressing crRNA-2 and crRNA-3. Conversely, plants treated with crRNA-1 showed negligible evidence of genome editing (Fig. 2). These quantitative results increased with time, reaching about 90% in the case of crRNA-3, suggesting that VIGE efficiency can intensify over time in inoculated plants, probably due to the accumulative effect of the editing complex. Notably, TRV was detected by RT-PCR in all the analyzed samples, supporting the notion that crRNA-1 is not an efficient guide RNA for this technology. Although predicted efficiencies of the three selected crRNAs were very similar based on CRISPOR results, *in vivo* assays showed that *in silico* results are not completely transferrable [21]. A complementary analysis revealed that the designed crRNAs effectively and consistently targeted both homeologous genes, achieving similar editing levels across both. Consequently, evaluating the editing outcome of just one of the *CHLI* homeologs can serve as a reliable indicator of overall functional efficacy in future comparative studies, significantly reducing sequencing needs. Short RNAs, about the size of the crRNA protospacers used in this work, has been recently shown to induce RNA silencing when expressed from a TRV vector [23]. We confirmed a contribution from *NbCHLI* silencing to the observed phenotype, likely arising from the expression of the 23-nt crRNA-3 protospacer. However, this is a minor contribution compared to the white patches resulting from CRISPR-based editing, likely arising from knocking-out the four *NbCHLI* alleles (Fig. S3).

In model organisms like *N. benthamiana* and *A. thaliana*, VIGE capacity to induce heritable modifications has been demonstrated when sgRNAs reach germline cells of SpCas9 expressing plants [7, 9]. Different studies have shown that fusing mobile RNA motifs to transcripts can increase their mobility in plants, and even direct them to the germ line [7, 24]. To achieve this goal, we compared four different designs based on the TRV-crRNA-2 vector. We obtained seedlings with at least one edited allele with all the assayed vectors, although the higher level of inheritance was achieved with the crRNA presenting one LbCas12a direct repeat at the 5′ end and a 102-nt fragment of the *A. thaliana FT* fused to the 3′ end, which is in accordance with previous works [7, 9]. Notably, adding a second direct repeat at the 3′ end, and therefore promoting a clean end of the crRNA after processing, does not increase significatively the editing efficiency in adult plants and in seedlings (Fig. 3). This strategy, however, could be used in a multiplexing approach, as Cas12a ability to process multimeric crRNAs containing two or more protospacer sequences separated by direct repeats has already been described [13, 35]. Taken together, our results demonstrate the feasibility of using the TRV-vector to obtain edited seedlings from *N. benthamiana* plants expressing LbCas12a and therefore bypassing tissue culture regeneration, similarly to what was previously shown in SpCas9-expressing plants [7, 32].

Despite recent remarkable VIGE advancements obtained with different viral vectors, such as TRV [7, 9, 36, 37], tomato spotted wilt virus (TSWV) [10] or barley yellow striate mosaic virus (BYSMV) [38], VIGE still necessitates substantial improvements to genuinely become a robust alternative to stable plant transformation. A primary impediment is the restricted host range of viral vectors. Each vector exhibits a specific host range, and even with the same vector, CRISPR outcomes can vary drastically across different hosts, including varieties within the same species, owing to unique host–virus interactions. While diverse viral vectors have demonstrated utility for CRISPR applications, the VIGE toolbox remains limited. Consequently, a key objective of this work was to broaden VIGE capabilities by incorporating vectors from the genus *Potyvirus*. Potyvirus-based viral vectors are particularly appealing because they constitute the largest group of RNA viruses infecting plants and, collectively, exhibit a broad host range, including major crops. They possess a positive-sense, single-stranded RNA genome of ~10 kb and are characterized by a gene expression strategy based on production of large polyproteins that are specifically processed by virus-encoded proteases [39]. Given that their replication mechanism hinges on this polyprotein processing, any engineered clones must preserve the correct translational reading frame to ensure proper processing and accurate protein formation. Therefore, when expressing exogenous sequences within a potyviral vector, it is crucial to avoid introducing any premature stop codons in the coding sequence, as this would impair viral replication. For this reason, we selected the crRNA-3, instead of the crRNA-2, because the former has no stop codons in its sequence. The crRNA-3 was then cloned in between the NIb and CP cistrons of a vector derived from TEV (Fig. 4).

Despite both homeologs of *NbCHLI* gene resulted edited in the same proportion when guides were delivered by TRV or TEV vectors, TEV-infected plants presented necrotic symptoms and died after 30 dpi. To overcome this situation, we selected four single-point mutations in the HC-Pro cistron, which were previously described to attenuate symptom development in infected plants [25]. Infections with TEV CLA2 and AS13a mutants led to plant survival and functional flower development. We then explored the feasibility of obtaining stable mutants from the infected plants (Fig. 5). On the one side, through tissue regeneration, we successfully recovered multiple fully white plants. ICE analysis confirmed that all alleles of both *NbCHLI* homeologs were edited, consistent with the complete inactivation of the reporter gene and the observed white phenotype. TEV presence was confirmed in all these regenerated plants. The need to resort on mild TEV mutants, such as CLA2 or AS13a in *N. benthamiana* shows the importance of infection symptoms induced by the viral vector. Design of VIGE approaches must consider this aspect for the plant to complete the biological cycle and produce seed or to collect infected tissue in enough good conditions to proceed with plant regeneration using *in vitro* culture techniques. On the other side, when seeds from agroinoculated plants were germinated *in vitro*, one seedling from a TEV-CLA2-crRNA-3–infected plant showed an edited allele, even though TEV was no longer detectable by RT-PCR. These compelling results indicate that the attenuated TEV vector is a suitable tool for VIGE, offering paths to obtain edited plants both through tissue culture regeneration and, significantly, through seed propagation where the virus could be segregated, although at a very low rate. It is worthy to note that crRNA expressed through potyviral constructs in this work did not contain mobile signals to facilitate entry into germplasm cells in meristematic tissues.

We aimed to replicate these experiments in agronomically important crops like cultivated tobacco and tomato. For this, we generated transgenic tobacco and tomato plants expressing a temperature-tolerant LbCas12a nuclease, specifically harboring the D156R mutant previously reported [26], and agroinoculated these transformed lines with TRV and TEV vectors carrying crRNA-3. Both vectors achieved 40%–50% ICE levels in both species (Fig. 6). Notably, the TEV vector did not require attenuation, as in the case of *N. benthamiana*, for these hosts to reach the flowering stage. Next, we evaluated the capacity to regenerate stable tobacco mutants from infected leaves showing editing symptoms. Similar to our *N. benthamiana* results, we successfully grew white plants on agar plates, all of which had all four of their alleles edited. Interestingly, one of the regenerated plants carrying one edited allele showed no detectable TEV via RT-PCR. Adding antiviral treatment to the regeneration media, as previously described, could further support the growth of edited, non-infected plants [10]. Finally, to further enhance the versatility of our VIGE strategy, we successfully demonstrated the implementation of additional potyviral vectors, such as TuMV and LMV (Fig. 7). This expands the potential number of plant hosts that could benefit from our gene-editing approach. While these vectors proved effective when tested in LbCas12a-expressing *N. benthamiana* plants, future research will focus on evaluating their performance in their natural hosts, brassica, and lettuce, following the development of the necessary transformed lines.

While expression of crRNAs in transgenic lines that stably express Cas nucleases is an important step forward, the major aim of VIGE is to co-express both the guide RNA and the Cas nuclease. This would make it possible to avoid the transformation process, thereby avoiding future regulatory problems when entering the market. Expressing Cas nucleases with a viral vector is a particularly difficult aim due to cargo limitation of plant virus-derived vectors. However, significant advances have been recently made in this regard. CRISPR edits were obtained in *N. benthamiana* wild-type plants when LbCas12a nuclease was expressed by means of a TEV vector lacking NIb protein, which was complemented with a potato virus X (PVX; genus *Potexvirus*), which also expressed the crRNA [18]. Moreover, using a vector derived from TSWV (genus *Orthosposvirus*), to express CRISPR-Cas reaction components, CRISPR edits were obtained not only in *N. benthamiana* plants, but also in crop plants such as tobacco, tomato, pepper, and peanut [10]. Further, BYSMV (genus *Cytorhabdovirus*) was recently used to deliver Cas9 in different wheat cultivars, achieving heritable gene editing. In addition, miniature variants less than half the size of Cas12a, such as Cas12j and Cas12f, have been developed and demonstrated functionality in editing of soybean and rice [40, 41]. Among these, the AsCas12f variant from *Acidibacillus sulfuroxidans* stands out due to its compactness, which has allowed for its successful integration into viral vectors like PVX, achieving efficient gene editing in both inoculated and systemic leaves of *N. benthamiana* [42]. TnpB is also an attractive candidate as a compact genome editor, whose use as a new type of genome editing tool has been shown in both monocot and dicot plant species [43]. Recently, this compact nuclease and its guide RNA has been successfully co-expressed from an engineered TRV vector. This system allowed generation of transgene-free editing of *A. thaliana* in a single step, with CRISPR edits inherited in the subsequent generation [12].

Future research is required to improve heritability using potyviral vectors and incorporation of miniature Cas nucleases to create fully transgene-free, tissue culture-free, and DNA-free genome editing [40, 42]. Ultimately, VIGE represents a substantial advance in plant biotechnology, capable of accelerating biological discoveries and offering a powerful, accessible, and potentially regulatory-compliant tool for precise genome engineering across a broad spectrum of economically vital crops and model plants.

## Materials and methods

### Design of crRNAs and vector construction

Both homeologs of *N. benthamiana CHLI* gene (*CHLI-A* and *CHLI-B*), were selected as targets for the VIGE LbCas12a system. crRNAs were designed using the CRISPOR online tool (http://crispor.tefor.net/) [21], specifically selecting sequences within exon 3 of both homeologs based on the specificity scores and trying to avoid guides presenting off-targets at other genomic loci different from those targeted. Genomic analysis determined that the region targeted by crRNA-3 was conserved also in both homologs of *N. tabacum CHLI* gene and contained a mismatch in *S. lycopersicon CHLI*.

Plasmids were built through standard molecular biology techniques including PCR amplification of DNA fragments with high-fidelity Phusion DNA polymerase (Thermo Scientific), restriction enzyme cleavage (Thermo Scientific) and the Gibson assembly reaction with the GeneArt Gibson assembly HiFi master mix (Invitrogen). pLX-TRV2 (GeneBank accession number OM372496) contains an engineered TRV2 cDNA, whose expression is driven by a CaMV 35S promoter and terminator, and presents a hepatitis delta virus-derived ribozyme at the 3′ end of the viral sequence [22]. This vector also incorporates a heterologous PEBV CP promoter to drive in planta crRNA expression [22]. pLX-TRV2 was digested with BsaI (BsaI-HFv2, New England Biolabs) and the cDNAs corresponding to the different crRNAs inserted to create plasmids pLX-TRV2-crRNA-1, -2, -3, -2(DR1-CHLI2-DR2), -2(DR1-CHLI2-5FT), and -2(DR1-CHLI2-DR2-5FT). The full sequences of all plasmids generated in this work was confirmed by Oxford Nanopore sequencing (Plasmidsaurus). Full sequences of the TRV2 segments of TRV vectors are in Fig. S2. Plasmid pGTEVa [44] contains the cDNA of an infectious TEV variant (GenBank DQ986288, with G273A and A1119G silent mutations), whose expression is driven by a CaMV 35S promoter and terminator within a binary vector derived from pCLEAN-G181 [45]. pGTEV-G2 (Fig. S6) is a pGTEVa-derivative with a MluI-Kpn2I polylinker between the NIb and CP cistrons. The cDNA corresponding to crRNA-3 was inserted in MluI-Kpn2I-digested pGTEV-G2 using the Gibson assembly reaction producing pGTEV-crRNA-3 (Fig. S6). In this TEV-based recombinant vector, the crRNA cDNA was strategically inserted between the NIb and CP flanked by sequences to complement the split NIb/CP proteolytic (NIaPro) site to allow releasing the exogenous peptide from the viral polyprotein (Fig. S4). Similarly, pGTuMVJPN1-crRNA-3 and pGLMV-crRNA-3 (Fig. S6) were created to express the crRNA-3 from TuMV (sequence variant JPN1) and LMV vectors (Fig. S4). These plasmids were built from pGTuMVJPN1-K and pGLMV-X2 (Fig. S6) by, respectively, KasI and XbaI digestion followed by Gibson assembly of the crRNA-3 cDNA.

Plasmids pGBKan-LbCas12att and pLBHig-LbCas12att (Fig. S5) were used for *N. benthamiana* (pGBKan-LbCas12att), and *N. tabacum* and *S. lycopersicum* (pLBHig-LbCas12att) stable transformation. Plasmid pLBHig-LbCas12att derives from minibinary vector pLX-B2 [46], which contains the broad host-range pBBR1 origin for plasmid replication suitable for both *Escherichia coli* and *A. tumefaciens*. The transfer DNA (T-DNA) contains the *Hygromycin phosphotransferase* (*hpt*) gene as a selection marker, under the control of the *A. tumefaciens Nopaline synthase* (*NoS*) promoter and terminator. pLBHig-LbCas12att expresses a temperature-tolerant version harboring the single mutation D156R of a LbCas12a nuclease previously reported [26, 27]. This nuclease was under the control of a CaMV double 35S promoter and a 35S terminator. *5′ and 3′ untranslated regions (UTR) of* cowpea mosaic virus (CPMV) flanked the coding region of the nuclease. The nuclease was *fused to two nuclear localization signals (NLS) from* nucleoplasmin and simian virus 40 (SV40) at the carboxyl terminus (Fig. S5)*.*

### Plant growth conditions and inoculation

Transgenic LbCas12a-expressing *N. benthamiana*, *N. tabacum*, and *S. lycopersicum* plants were grown under controlled conditions at 25°C and a 16-hour day/8-hour night cycle. In the case of tobacco and *N. benthamiana*, fully expanded upper leaves from 4- to 6-week-old plants were used for agroinoculation with plasmids carrying the viral vectors expressing the different crRNA. For tomato plants, the first true leaf and one cotyledon were agroinoculated.

*A. tumefaciens* C58C1 electrocompetent cells carrying the pLX-TRV1 plasmid [22] were electroporated with pLX-TRV2 derivatives and selected on Luria–Bertani (LB) plates supplemented with rifampicin (50 μg/ml), gentamicin (20 μg/ml), and kanamycin (50 μg/ml). The same *A. tumefaciens* strain, previously transformed with the pCLEAN-S48 helper plasmid [45] was transformed with pGTEV-crRNA-3, pGTuMVJPN1-crRNA-3, and pGLMV-crRNA-3. Transformed bacteria, in this case, were selected on LB plates supplemented with rifampicin (50 μg/ml), kanamycin (50 μg/ml) and tetracycline (7.5 μg/ml).

Individual colonies were selected and grown for 24 hours at 28°C in liquid LB media supplemented with 50 μg/ml kanamycin and 20 μg/ml of gentamicin (TRV clones) or only 50 μg/ml kanamycin (TEV, TuMV, and LMV clones). When cultures reached an optical density at 600 nm (OD600) from 1 to 2, cells were pelleted by centrifugation and resuspended to an OD600 of 0.5 in inoculation buffer (10 mM 2-(N-morpholino)ethanesulfonic acid (MES)–NaOH) pH 5.6, 10 mM MgCl_2_, supplemented with 150 μM acetosyringone [47]. Bacterial cultures were induced for 2 hours at 28°C. The abaxial side of selected leaves or cotyledons were infiltrated using a needle-less 1-ml syringe. After infiltration, plants were transferred to a growth chamber under a 16-hour day/8-hour night cycle at 25°C. At different times after inoculation, as indicated in the different experiments, systemic non-inoculated leaves were sampled (1 cm^2^ approximately), immediately frozen in liquid nitrogen and stored at −80°C until they were processed.

### Analysis of LbCas12-mediated gene editing

Plant tissues were ground in 2-ml Eppendorf tubes containing a 4-mm diameter stainless steel ball, and using a VWR Star-Beater mill, for 1 minutes at 30 s^−1^. Extraction buffer (4 M guanidinium thiocyanate, 0.1 M sodium acetate, 10 mM EDTA and 0.1 M 2-mercaptoethanol, pH 5.5) was previously added to each tube. After homogenization, samples were centrifuged at 15000×*g* for 5 minutes. An aliquot of the supernatant (0.7 ml) was transferred to a silica-gel column (Zymo Research). After centrifugation at 15000×*g* for 1 min, the column was washed twice with 0.5 ml of washing buffer (70% ethanol, 10 mM sodium acetate, pH 5.5). The DNA was eluted from the column in 10 μl of 20 mM Tris–HCl, pH 8.5.

*Nicotiana benthamiana CHLI-A* and *CHLI-B* target DNA fragments corresponding to both homologs were individually amplified by PCR using the high-fidelity Phusion DNA polymerase and primers 5′-TTACGATAGGAAGTATATTT-3′ and 5′-AACTCGAAGATCATGATCTA-3′ (for *NbCHLI-A*) or 5′-TTACGACACGAAGTATATAG-3′ and 5′-AACTCGAAGATCATGAGCAG-3′ (for *NbCHLI-B)*. In the case of *N. tabacum* samples, a primer pair amplifying both homeologs at the same time was used (5′-GGAGGAGAGCCAGAGACCGGTG-3′ and 5′-TTAGTTCTGCACAGACCTTAG-3′). To amplify the target region of *SlCHLI*, primer pair 5′-ACAGCCAGAGACCGGTGTATCC-3′ and 5′-CTTGGGCATGCATTCCAAATCG-3′ was used. PCR products were separated by agarose gel (1%) electrophoresis, purified from the gel, and subjected to Sanger sequencing. The presence of sequence modifications was analyzed using the ICE software by the EditCo Bio (https://ice.editco.bio/).

### RNA purification and analysis of virus infectivity

To verify the presence of TRV in *N. benthamiana* inoculated in plants as well as the presence of TEV in *N. benthamiana* and *N. tabacum* seedlings and plants regenerated from tissue culture, RT-PCR were performed. Total RNA was purified from leaf tissue using silica-gel columns (Zymo Research). The protocol followed was the same as previously described for DNA isolation, with an additional step. Before loading into the column, the supernatant was mixed with 0.65 volumes of 96% ethanol and centrifuged at 15000×*g* for 1 minute. Aliquots of 1 μl of the purified RNA were used for reverse transcription (RT) with RevertAid reverse transcriptase (Thermo Scientific), using 5′-GAATATGGTATCACCCACCCTC-3′ primer for TRV analysis or 5′-TCATAACCCAAGTTCCGTTC-3′ primer for TEV analysis. Aliquots (1 μl) of the obtained cDNAs were used for PCR amplification with primers 5′-ATGGGAGATATGTACGATGAAT-3′ and 5′-GGGATTAGGACGTATCGGACC-3′ in the case of TRV or with primers 5′-CATCTGTGCATCAATGATCGAA-3′ and 5′-GTGTGGCTCGAGCATTTGACAA-3′ in the case of TEV. PCR products were analyzed by electrophoresis in 1% agarose gels that were stained with ethidium bromide.

### Plant stable transformation

Transgenic LbCas12a-expressing *N. benthamiana* plants were obtained as previously described by Bernabé-Orts et al [13]. Plant transformation and regeneration were done following modifications of standard procedures for *N. tabacum* [48] and *S. lycopersicum* [49] using the *A. tumefaciens* strain LBA4404. *In vitro* steps were carried out under light, humidity, and temperature-controlled conditions (16-hour light/8-hour dark; 26 ± 2°C, 40% relative humidity during the light period; 22 ± 2°C, 70% relative humidity during the dark period). Selective media for plant regeneration was supplemented with 5 μg/ml of hygromycin. For both species, T-DNA detection in regenerated plants was performed by PCR using primers 5′-ATAAGGAGACAGACTATCGGGC-3′ and 5′-TGATCTGTCCGTGATTGTTCTC-3′ to amplify a fragment of LbCas12a cDNA.

### *In vitro* regeneration of edited *N. benthamiana* and *N. tabacum* plants

Upper leaves showing symptoms of viral infection were collected and submerged in water for 30 minutes to remove dirt and dust present on the surface. After that, leaves were surface-sterilized by submersion in 50% commercial bleach solution for 10 min, and subsequently washed three times in sterile water. Sterilized leaves were cut into pieces of ~1 cm^2^ using a scalpel, and were transferred to an organogenesis media, composed of 4.3 g/l Murashige and Skoog (MS) with vitamins, 30 g/l sucrose, 8 g/l bacteriological agar, 1 mg/l of 6-benzylaminopurine (BAP), and 0.1 mg/l 1-naphthaleneacetic acid (NAA), pH 5.8. Leaf explants were transferred to fresh plates every 2 weeks until shoots emerged. Shoots were cut and transferred to shoot elongation media, composed of 4.3 g/l MS with vitamins, 30 g/l sucrose, 8 g/l bacteriological agar, 0.1 mg/l BAP, and 0.1 mg/l NAA, pH 5.8. Elongated shoots were transferred to root induction media, composed of 4.3 g/l MS with vitamins, 30 g/l sucrose, 7 g/l bacteriological agar, and 0.2 mg/l NAA, pH 5.8. Regenerated plantlets with roots were analyzed for editing of the target genes in the case of *N. benthamiana*. In the case of *N. tabacum*, elongated unrooted shoots were analyzed.

### Seed germination of *N. benthamiana* and *N. tabacum*

*Nicotiana benthamiana* seeds were collected from infected plants. Seeds from different flowers were pooled in the same Eppendorf tube, and they were sterilized with 50% bleach and 0.1% Nonidet P-40, and washed with sterile water three times before sowing on plates with germination media (4.3 g/l MS, 0.1 g/l MES, 20 g/l sucrose, 7.5 g/l agar, pH 5.8). Once germinated, seedlings were collected for genome extraction and ICE analysis. In the case of *N. tabacum* seeds, the collection method was the same as described above. Seeds were directly sown on soil mixture composed of one part of perlite and two parts of potting substrate. Pots were located in a growth chamber set at 16-hour light/8-hour dark and 25°C until cotyledons emerged and samples were taken.

## Supplementary Material

Web_Material_uhag017

## Data Availability

The data that supports the findings of this study are in Supplementary material.
