# Supplementary material for: Virus induced gene editing using potyviral vectors in Cas12a expressing plants
Source: Hortic Res. 2026 Jan 20;13(4):uhag017. doi: 10.1093/hr/uhag017 (PMC13098375; doi:10.1093/hr/uhag017)
Supplement: Web_Material_uhag017 [file web_material_uhag017.pdf]

SUPPLEMENTARY DATA

Virus induced gene editing using potyviral vectors in LbCas12a expressing plants

Fernando Merwaiss*, Arcadio García, Ugo Rogo, Ivana Querol-Martí, Begoña García-Sogo, Benito Pineda, Vicente Moreno, Carmine de Paola, Marta Rodríguez-Rodríguez, Marta Vázquez, Diego Orzáez and José-Antonio Daròs*

Instituto de Biología Molecular y Celular de Plantas (CSIC-Universitat Politècnica de València), 46022 Valencia, Spain.

* Correspondence (email fmerwaiss@upv.es; jadaros@ibmcp.upv.es)

Figure S1. Selected crRNAs that simultaneously target both homeologs of *NbCHLI* (*CHLI-A*, chromosome 14; and *CHLI-B*, chromosome 12).

```
CHLI-A   ATGGCTTCACTACTAGGAACTTCCTCTTC---AGCAGCAGCTGCAGTATTAGCTTCTACACCCTTATCTTCTCGCTCCTCTAAATCTTCCATTTTCTCTCTCCTCCCTTCTTCAGGGCAG
CHLI-B   ATGGCTTCACTATTAGGAACTTCCTCTTCAGCAGCAGCTGCTGCAATATTAGCTTCTACACCCTTATCTTCTCGCTCCTCTAAATCTTCCATTTTCTCCCTCTTCCCTTCTTCAGGGCAG
         ************ ****************   ****** ****** **************************************************** *** *****************

CHLI-A   AGTCAAGGGAGGAAGTTTTATGGAGGGATTAGAGTCCCGGTTAAAAAAGGGAGGTCCCAATTCCATGTCGCAATTTCAAATGTTGCGACGGAAATCAGCCCTGCTCAAGAACAGGGTCAG
CHLI-B   TGTCAAGGGAGGAAGTTTTATGGAGGGATTAGAATCCCAGTTAAGAAAGGGAGGTCCCAATTGCATGTGGCAATTTCAAATGTTGCAACGGAAGTCAGCCCTGCTCAAGAACAGGGTCAG
          ******************************** **** ***** ***************** ***** ***************** ****** **************************

CHLI-A   AAACTTGCTGAGGAGAGCCAGAGACCGGTGTATCCATTTGCAGCTATAGTGGGACAAGATGAAATGAAGTTATGTCTTTTGCTGAATGTAATTGATCCAAAGATTGGAGGTGTGATGATA
CHLI-B   AAACTTGCTGAGGAGAGCCAGAGACCAGTGTATCCATTTGCAGCTATAGTGGGACAAGACGAGATGAAGTTATGTCTTTTGCTGAATGTAATTGATCCAAAGATTGGAGGTGTGATGATA
         ************************** ******************************** ** *********************************************************

CHLI-A   ATGGGTGATAGAGGAACCGGGAAGTCCACCACGGTTAGATCTTTGGTAGATTTACTTCCTGATATCAAAGTTATTTCTGGTGATCCGTTCAATTCAGATCCAGATGACCAAGAAGTAATG
CHLI-B   ATGGGTGATAGAGGAACCGGGAAGTCCACCACGGTTAGATCTTTGGTAGATTTACTTCCTGAAATTAAAGTTATTTCTGGTGATCCGTTCAATTCAGATCCAGATGACCAAGAAGTAATG
         ************************************************************** ** ******************************************************

CHLI-A   AGTGCAGAAGTCCGTGACAAATTGAGGAGCGGAGAAGCTTCCTATATCTCGTACCAAAATCAACATGGTTGATTTACCGCTAGGTGCTACTGAGGACAGGGTGTGTGGCACAATCGAC
CHLI-B   AGCGCAGAAGTCCGTGACAAATTGAGGAGCGGAGAAGCTTCCTATATCTCGTACTAAAATCAACATGGTTGATTTACCGCTAGGTGCTACTGAGGACAGGGTATGTGGCACAATCGAC
         ** *************************************************** *********************************************** ***************

CHLI-A   ATTGAGAAAGCTCTTACTGAGGGTGTGAAGGCTTTCGAGCCTGGTCTTCTTGCTAAAGCTAACAGAGGAATTCTTTATGTCGATGAGGTTAATCTTTTGGACGACCATTTAGTAGATGTT
CHLI-B   ATTGAGAAAGCTCTTACTGAGGGTGTGAAGGCTTTCGAGCCTGGTCTTCTTGCTAAAGCTAACAGAGGAATACTTTACGTCGATGAGGTTAATCTTTTGGACGACCATTTAGTAGATGTT
         *********************************************************************** ***** ******************************************

CHLI-A   CTTTTGGATTCTGCAGCATCGGGATGGAACACTGTTGAAAGAGAGGGGATATCAATATCACACCCTGCCCGATTTATCCTTATTGGTTCGGGTAATCCTGAAGAAGGAGAACTTAGGCCA
CHLI-B   CTTTTGGATTCTGCAGCATCAGGATGGAACACTGTTGAAAGAGAGGGGATTTCAATCTCACATCCGGCCCGATTTATCCTAATTGGTTCGGGTAATCCTGAAGAAGGAGAACTTAGGCCA
         ******************** ***************************** ***** ***** ** ************** ***************************************

CHLI-A   CAACTTCTTGATCGATTTGGAATGCATGCCCAAGTGGGGACCGTGAGAGATGCAGAGCTGAGAGTGAAGATCGTTGAGGAAAGAGCTCGTTTTGATAAGAACCCCAAGGAATTCCGGGAG
CHLI-B   CAACTTCTTGATCGATTTGGAATGCATGCCCAAGTGGGGACCGTGAGAGATGCAGAGCTGAGAGTAAAGATCGTTGAGGAAAGAGCTCGTTTTGATAAGAACCCCAAGGAATTCCGGGAG
         ***************************************************************** ******************************************************

CHLI-A   TCATACAAGGCAGAGCAAGAAAAGCTCCAGAATCAAATCGACTCAGCTAGGAACGCTCTTTCTGCTGTTACAATAGATCATGATCTTCGAGTTAAAATCTCTAAGGTCTGTGCAGAACTG
CHLI-B   TCATACAAGGCAGAGCAAGAAAAGCTCCAGAATCAAATCGACTCAGCTAGGAACGCTCTTTCTGCTGTTACAACTGCTCATGATCTTCGAGTTAAAATCTCCAAGGTCTGTGCAGAACTA
         *************************************************************************  * ************************ ***************** 

CHLI-A   AACGTCGATGGATTGAGAGGTGATATAGTCACTAACAGGGCAGCAAGAGCGTTGGCTGCACTAAAAGGAAGAGATAAGGTCACTCCGGAGGATATCGCCACTGTCATTCCCAACTGCTTA
CHLI-B   AATGTCGATGGACTTAGAGGTGATATAGTCACTAACAGGGCAGCACGAGCGTTGGCTGCACTAAAAGGAAGAGATAAGGTAACTCCGGAGGATATTGCCACTGTCATTCCCAACTGCTTA
         ** ********* * ***************************** *********************************** ************** ************************

CHLI-A   AGACACAGACTGAGGAAGGATCCTTTGGAGTCTATTGACTCGGGTGTTCTTGTTGTTGAGAAATTTTATGAGGTTTTCGCCTAA
CHLI-B   AGACACAGACTGAGGAAGGATCCTTTGGAGTCTATTGACTCGGGTGTACTTGTTGTTGAGAAATTTTATGAGGTTTTCGCCTAA
         *********************************************** ************************************
```

Targets of crRNA-1, crRNA-2 and crRNA-3 are highlighted on blue, green and yellow, backgrounds, respectively. LbCas12a PAMs are underlined.

Figure S2. Nucleotide sequences of the TRV RNA2 (GenBank accession number OM372496)-derived clones that constitute, along with a wild-type TRV1 (OM372495), the different TRV vectors.

>TRV2-crRNA-1 (segment 2 of TRV-crRNA-1)
ATAAAACATTGCACCTATGGTGTTGCCCTGGCTGGGGTATGTCAGTGATCGCAGTAGAATGTACTAATTGACAAG
TTGGAGAATACGGTAGAACGTCCTTATCCAACACAGCCTTTATCCCTCTCCCTGACGAGGTTTTTGTCAGTGTAA
TATTTCTTTTTGAACTATCCAGCTTAGTACCGTACGGGAAAGTGACTGGTGTGCTTATCTTTGAAATGTTACTTT
GGGTTTCGGTTCTTTAGGTTAGTAAGAAAGCACTTGTCTTCTCATACAAAGGAAAACCTGAGACGTATCGCTTAC
GAAAGTAGCAATGAAAGAAAGGTGGTGGTTTTAATCGCTACCGCAAAAACGATGGGGTCGTTTTAATTAACTTCT
CCTACGCAAGCGTCTAAACGGACGTTGGGGTTTTGCTAGTTTCTTTAGAGAAAACTAGCTAAGTCTTTAATGTTA
TCATTAGAGATGGCATAAATATAATACTTGTGTCTGCTGATAAGATCATTTTAATTTGGACGATTAGACTTGTTG
AACTACAGGTTACTGAATCACTTGCGCTAATCAAC**ATG**GGAGATATGTACGATGAATCATTTGACAAGTCGGGCG
GTCCTGCTGACTTGATGGACGATTCTTGGGTGGAATCAGTTTCGTGGAAAGATCTGTTGAAGAAGTTACACAGCA
TAAAATTTGCACTACAGTCTGGTAGAGATGAGATCACTGGGTTACTAGCGGCACTGAATAGACAGTGTCCTTATT
CACCATATGAGCAGTTTCCAGATAAGAAGGTGTATTTCCTTTTAGACTCACGGGCTAACAGTGCTCTTGGTGTGA
TTCAGAACGCTTCAGCGTTCAAGAGACGAGCTGATGAGAAGAATGCAGTGGCGGGTGTTACAAATATTCCTGCGA
ATCCAAACACAACGGTTACGACGAACCAAGGGAGTACTACTACTACCAAGGCGAACACTGGCTCGACTTTGGAAG
AAGACTTGTACACTTATTACAAATTCGATGATGCCTCTACAGCTTTCCACAAATCTCTAACTTCGTTAGAGAACA
TGGAGTTGAAGAGTTATTACCGAAGGAACTTTGAGAAAGTATTCGGGATTAAGTTTGGTGGAGCAGCTGCTAGTT
CATCTGCACCGCCTCCAGCGAGTGGAGGTCCGATACGTCCTAATCCC**TAG**GGATTTAAGGACGTGAACTCTGTTG
AGATCTCTGTGAAATTCAGAGGGTGGGTGATACCATATTCACTGATGCCATTAGCGACATCTAAATAGGGCTAAT
TGTGACTAATTTGAGGGAATTTCCTTTACCATTGACGTCAGTGTCGTTGGTAGCATTTGAGTTTCGCAATGCACG
AATTACTTAGGAAGTGGCTTGACGACACTAATGTGTTATTGTTAGATAATGGTTTGGTGGTCAAGGTACGTAGTA
GAGTCCCACATATTCGCACGTATGAAGTAATTGGAAAGTTGTCAGTTTTTGATAATTCACTGGGAGATGATACGC
TGTTTGAGGGAAAAGTAGAGAACGTATTTGTTTTTATGTTCAGGCGGTTCTTGTGTGTCAACAAAGATGGACATT
GTTACTCAAGGAAGCACGATGAGCTTTATTATTACGGACGAGTGGACTTAGATTCTGTGAGTAAGGTTACCGAAT
TC**TGCACACAAAGTTAAAAACGCTGTAGTAATACATGCGCAAGAACAGGCTGAGCATCTTGTCCTGGGGTTTCAC**
ACTATCTTTAGAGAAAGTGTTAAGTTAATTAAGTTATCTTAATTAAGAGCATAATTATACTGATTTGTCTCTCGT
TGATAGAGTCTATCATTCTGTTACTAAAAAATTTGACAACTCGGTTTGCTGACCTACTGGTTACTGTATCACTTA
CCCGAGTTAACGCCTAATTTCTACTAAGTGTAGATTGGTGATCCGTTCAATTCAGATCATGTCCCGAAGACATTA
AACTACGGTTCTTTAAGTAGATCCGTGTCTGAAGTTTTAGGTTCAATTTAAACCTACGAGATTGACATTCTCGAC
TGATCTTGATTGATCGGTAAGTCTTTTGTAATTTAATTTTCTTTTTGATTTTATTTTAAATTGTTATCTGTTTCT
GTGTATAGACTGTTTGAGATCGGCGTTTGGCCGACTCATTGTCTTACCATAGGGGAACGGACTTTGTTTGTGTTG
TTATTTTATTTGTATTTTATTAAAATTCTCAACGATCTGAAAAAGCCTCGCGGCTAAGAGATTGTTGGGGGGTGA
GTAAGTACTTTTAAAGTGATGATGGTTACAAAGGCAAAAGGGGTAAAACCCCTCGCCTACGTAAGCGTTATTACG
CCC

>TRV2-crRNA-2 (segment 2 of TRV-crRNA-2)
Insert between positions 1889 and 1934 of TRV2-crRNA-1
TAATTTCTACTAAGTGTAGATCCGCTAGGTGCTACTGAGGACAG

>TRV2-crRNA-3 (segment 2 of TRV-crRNA-3)
Insert between positions 1889 and 1934 of TRV2-crRNA-1
TAATTTCTACTAAGTGTAGATGCAAGAAGACCAGGCTCGAAAGC

>TRV2-crRNA-2(DR1-CHLI2-DR2) (segment 2 of TRV-crRNA-2(DR1-CHLI2-DR2))
Insert between positions 1889 and 1934 of TRV2-crRNA-1
TAATTTCTACTAAGTGTAGATCCGCTAGGTGCTACTGAGGACAGTAATTTCTACTAAGTGTAGAT

>TRV2-crRNA-2(DR1-CHLI2-5FT) (segment 2 of TRV-crRNA-2(DR1-CHLI2-5FT))
Insert between positions 1889 and 1934 of TRV2-crRNA-1
TAATTTCTACTAAGTGTAGATCCGCTAGGTGCTACTGAGGACAGATGTCTATAAATATAAGAGACCCTCTTATAG
TAAGCAGAGTTGTTGGAGACGTTCTTGATCCGTTTAATAGATCAATCACTCTAAAGGTTACTTATGGCCAA

>TRV2-crRNA-2(DR1-CHLI2-DR2-5FT) (segment 2 of TRV-crRNA-2(DR1-CHLI2-DR2-5FT))
Insert between positions 1889 and 1934 of TRV2-crRNA-1

TAATTTCTACTAAGTGTAGATCCGCTAGGTGCTACTGAGGACAGTAATTTCTACTAAGTGTAGATATGTCTATAA ATATAAGAGACCCTCTTATAGTAAGCAGAGTTGTTGGAGACGTTCTTGATCCGTTTAATAGATCAATCACTCTAA AGGTTACTTATGGCCAA

Start and **stop** codons of TRV CP are underlined. Heterologous **pea early-browning virus (PEBV) subgenomic promoter** is indicated in blue. **LbCas12a direct repeats (DR)** are highlighted on yellow background, **protospacers** on green and the *A. thaliana* **5’ fragment of *Flowering locus T* (5FT)** mRNA on blue.

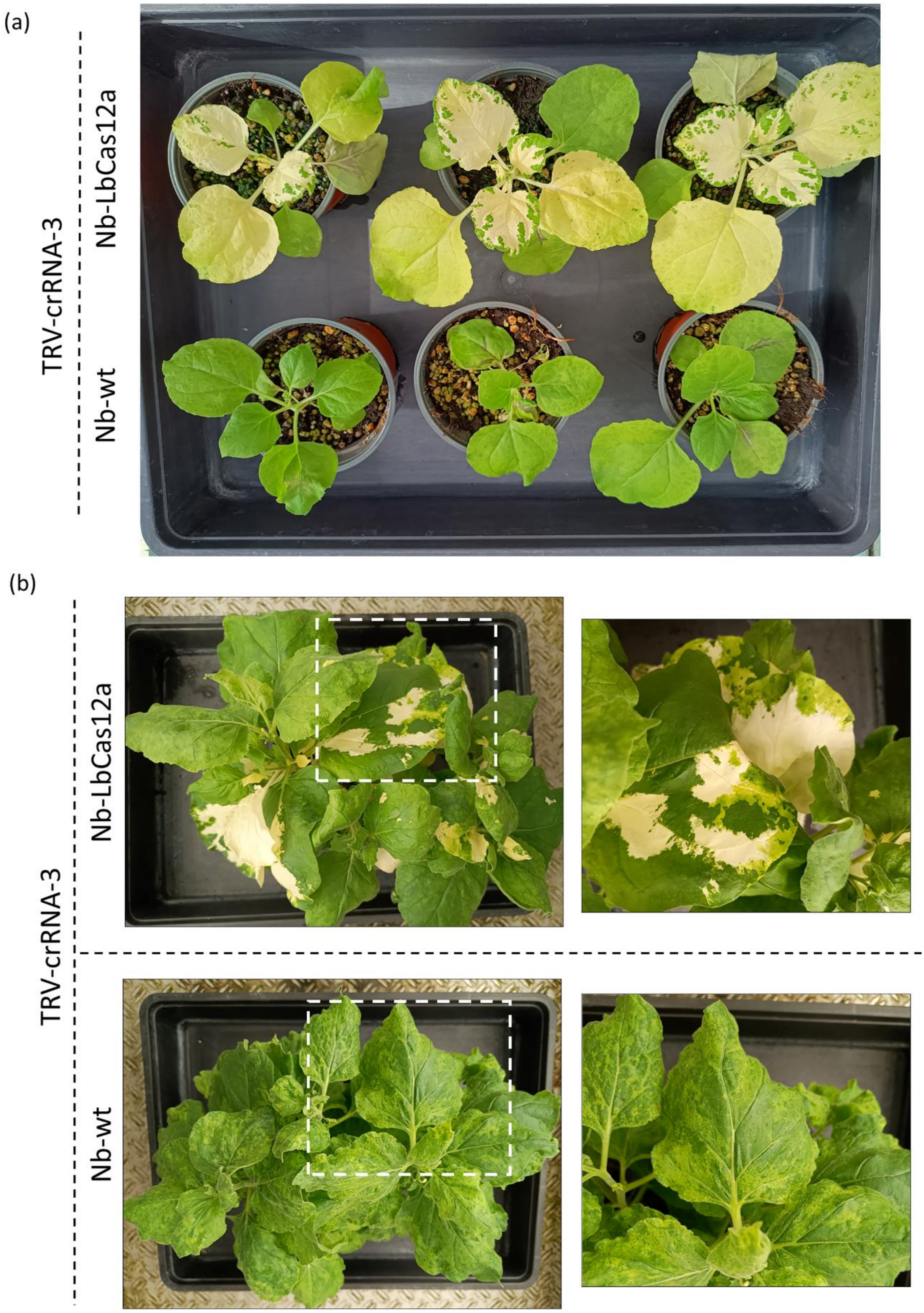

(a)
TRV-crRNA-3
Nb-LbCas12a
Nb-wt
(b)
TRV-crRNA-3
Nb-LbCas12a
Nb-wt

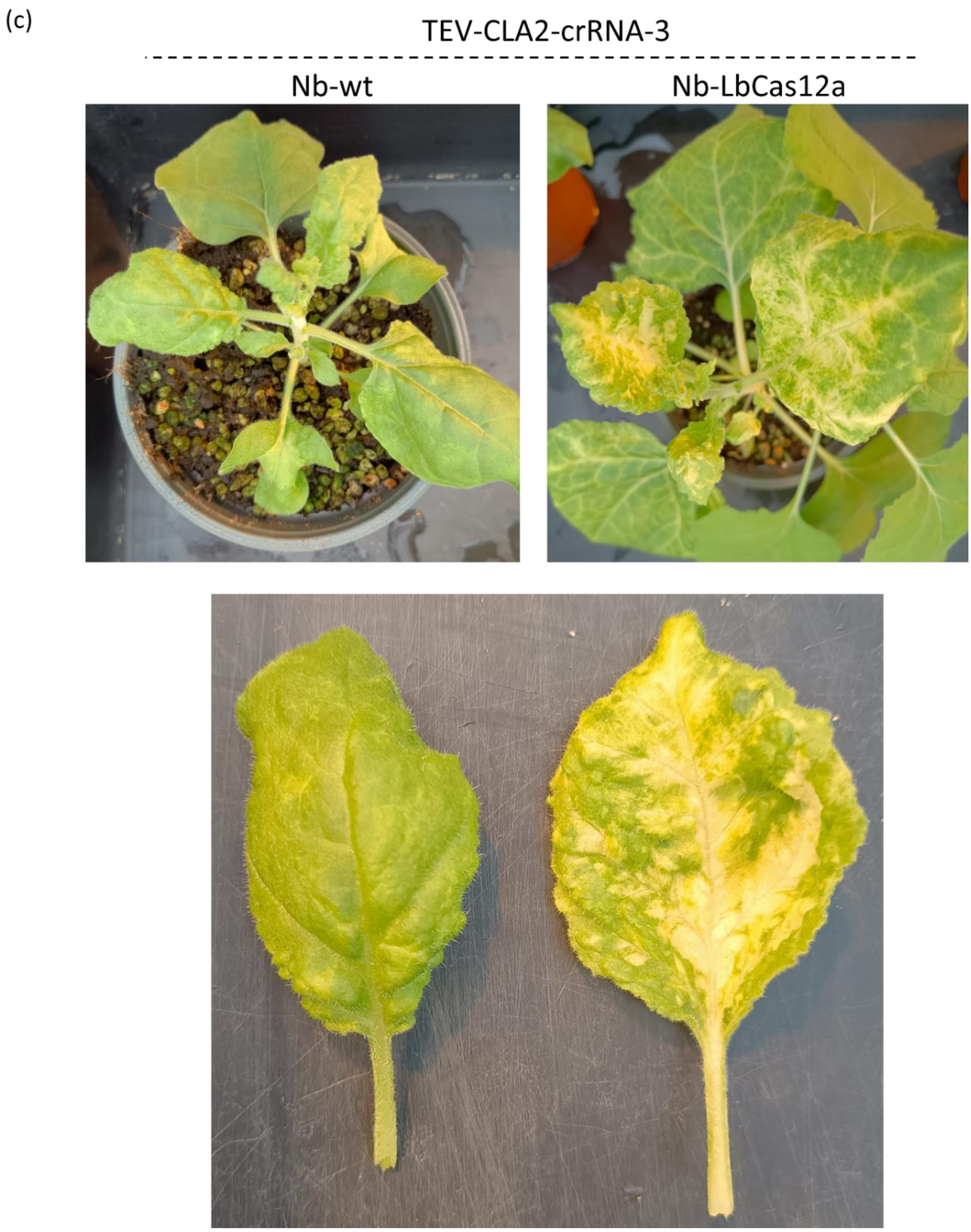

Figure S3. Phenotypical differences between *N. benthamiana* wild-type (Nb-wt) and LbCas12a-expressing plants (Nb-LbCas12a) infected with the (a and b) TRV-crRNA-3 and (c) TEV-CLA2-crRNA-3 vectors targeting both homeologs of *NbCHLI*. Pictures taken at (a) 12, (b) 30 and (c) 18 dpi. (b) Details of the boxed areas are shown on the right.

Figure S4. Nucleotide sequence of potyviral vectors expressing crRNA-3.

>TEV-crRNA-3

GAAAATAACAAATCTCAACACAACATATACAAAACAAACGAATCTCAAGCAATCAAGCATTCTACTTCTATTGCA
GCAATTTAAATCATTTCTTTTAAAGCAAAAGCAATTTTCTGAAAATTTTCACCATTTACGAACGATAGCCATGGC
ACTCATCTTTGGCACAGTCAACGCTAACATCCTGAAGGAAGTGTTCGGTGGAGCTCGTATGGCTTGCGTTACCAG
CGCACATATGGCTGGAGCGAATGGAAGCATTTTGAAGAAGGCAGAAGAAACCTCTCGTGCAATCATGCACAAACC
AGTGATCTTCGGAGAAGACTACATTACCGAGGCAGACTTGCCTTACACACCACTCCATTTAGAGGTCGATGCTGA
AATGGAGCGGATGTATTATCTTGGTCGTCGCGCGCTCACCCATGGCAAGAGACGCAAAGTTTCTGTGAATAACAA
GAGGAACAGGAGAAGGAAAGTGGCCAAAACGTACGTGGGGCGTGATTCCATTGTTGAGAAGATTGTAGTGCCCCA
CACCGAGAGAAAGGTTGATACCACAGCAGCAGTGGAAGACATTTGCAATGAAGCTACCACTCAACTTGTGCATAA
TAGTATGCCAAAGCGTAAGAAGCAGAAAAACTTCTTGCCCGCCACTTCACTAAGTAACGTGTATGCCCAAACTTG
GAGCATAGTGCGCAAACGCCATATGCAGGTGGAGATCATTAGCAAGAAGAGCGTCCGAGCGAGGGTCAAGAGATT
TGAGGGCTCGGTGCAATTGTTCGCAAGTGTGCGTCACATGTATGGCGAGAGGAAAAGGGTGGACTTACGTATTGA
CAACTGGCAGCAAGAGACACTTCTAGACCTTGCTAAAAGATTTAAGAATGAGAGAGTGGATCAATCGAAGCTCAC
TTTTGGTTCAAGTGGCCTAGTTTTGAGGCAAGGCTCGTACGGACCTGCGCATTGGTATCGACATGGTATGTTCAT
TGTACGCGGTCGGTCGGATGGGATGTTGGTGGATGCTCGTGCGAAGGTAACGTTCGCTGTTTGTCACTCAATGAC
ACATTATAGCGACAAATCAATCTCTGAGGCATTCTTCATACCATACTCTAAGAAATTCTTGGAGTTGAGGCCAGA
TGGAATCTCCCATGAGTGTACAAGAGGAGTATCAGTTGAGCGGTGCGGTGAGGTGGCTGCAATCCTGACACAAGC
ACTTTCACCGTGTGGTAAGATCACATGCAAACGTTGCATGGTTGAAACACCTGACATTGTTGAGGGTGAGTCGGG
AGACAGTGTCACCAACCAAGGTAAGCTCCTAGCAATGCTGAAAGAACAGTATCCAGATTTCCCAATGGCCGAGAA
ACTACTCACAAGGTTTTTGCAACAGAAATCACTAGTAAATACAAATTTGACAGCCTGCGTGAGCGTCAAACAACT
CATTGGTGACCGCAAACAAGCTCCATTCACACACGTACTGGCTGTCAGCGAAATTCTGTTTAAAGGCAATAAACT
AACAGGGGCCGATCTCGAAGAGGCAAGCACACATATGCTTGAAATAGCAAGGTTCTTGAACAATCGCACTGAAAA
TATGCGCATTGGCCACCTTGGTTCTTTCAGAAATAAAATCTCATCGAAGGCCCATGTGAATAACGCACTCATGTG
TGATAATCAACTTGATCAGAATGGGAATTTTATTTGGGGACTAAGGGGTGCACACGCAAAGAGGTTTCTTAAAGG
ATTTTTCACTGAGATTGACCCAAATGAAGGATACGATAAGTATGTTATCAGGAAACATATCAGGGGTAGCAGAAA
GCTAGCAATTGGCAATTTGATAATGTCAACTGACTTCCAGACGCTCAGGCAACAAATTCAAGGCGAAACTATTGA
GCGTAAAGAAATTGGGAATCACTGCATTTCAATGCGGAATGGTAATTACGTGTACCCATGTTGTTGTGTTACTCT
TGAAGATGGTAAGGCTCAATATTCGGATCTAAAGCATCCAACGAAGAGACATCTGGTCATTGGCAACTCTGGCGA
TTCAAAGTACCTAGACCTTCCAGTTCTCAATGAAGAGAAAATGTATATAGCTAATGAAGGTTATTGCTACATGAA
CATTTTCTTTGCTCTACTAGTGAATGTCAAGGAAGAGGATGCAAAGGACTTCACCAAGTTTATAAGGGACACAAT
TGTTCCAAAGCTTGGAGCGTGGCCAACAATGCAAGATGTTGCAACTGCATGCTACTTACTTTCCATTCTTTACCC
AGATGTCCTGAGTGCTGAATTACCCAGAATTTTGGTTGATCATGACAACAAAACAATGCATGTTTTGGATTCGTA
TGGGTCTAGAACGACAGGATACCACATGTTGAAAATGAACACAACATCCCAGCTAATTGAATTCGTTCATTCAGG
TTTGGAATCCGAAATGAAAACTTACAATGTTGGAGGGATGAACCGAGATATGGTCACACAAGGTGCAATTGAGAT
GTTGATCAAGTCCATATACAAACCACATCTCATGAAGCAGTTACTTGAGGAGGAGCCATACATAATTGTCCTGGC
AATAGTCTCCCCTTCAATTTTAATTGCCATGTACAACTCTGGAACTTTTGAGCAGGCGTTACAAATGTGGTTGCC
AAATACAATGAGGTTAGCTAACCTCGCTGCCATCTTGTCAGCCTTGGCGCAAAAGTTAACTTTGGCAGACTTGTT
CGTCCAGCAGCGTAATTTGATTAATGAGTATGCGCAGGTAATTTTGGACAATCTGATTGACGGTGTCAGGGTTAA
CCATTCGCTATCCCTAGCAATGGAAATTGTTACTATTAAGCTGGCCACCCAAGAGATGGACATGGCGTTGAGGGA
AGGTGGCTATGCTGTGACCTCTGAAAAGGTGCATGAAATGTTGGAAAAAAACTATGTAAAGGCTTTGAAGGATGC
ATGGGACGAATTAACTTGGTTGGAAAAATTCTCCGCAATCAGGCATTCAAGAAAGCTCTTGAAATTTGGGCGAAA
GCCTTTAATCATGAAAAACACCGTAGATTGCGGCGGACATATAGACTTGTCTGTGAAATCGCTTTTCAAGTTCCA
CTTGGAACTCCTGAAGGGAACCATCTCAAGAGCCGTAAATGGTGGTGCAAGAAAGGTAAGAGTAGCGAAGAATGC
CATGACAAAAGGGGTTTTTCTCAAAATCTACAGCATGCTTCCTGACGTCTACAAGTTTATCACAGTCTCGAGTGT
CCTTTCCTTGTTGTTGACATTCTTATTTCAAATTGACTGCATGATAAGGGCACACCGAGAGGCGAAGGTTGCTGC
ACAGTTGCAGAAAGAGAGCGAGTGGGACAATATCATCAATAGAACTTTCCAGTATTCTAAGCTTGAAAATCCTAT
TGGCTATCGCTCTACAGCGGAGGAAAGACTCCAATCAGAACACCCCGAGGCTTTCGAGTACTACAAGTTTTGCAT
TGGAAAGGAAGACCTCGTTGAACAGGCAAAACAACCGGAGATAGCATACTTTGAAAAGATTATAGCTTTCATCAC
ACTTGTATTAATGGCTTTTGACGCTGAGCGGAGTGATGGAGTGTTCAAGATACTCAATAAGTTCAAAGGAATACT
GAGCTCAACGGAGAGGGAGATCATCTACACGCAGAGTTTGGATGATTACGTTACAACCTTTGATGACAATATGAC
AATCAACCTCGAGTTGAATATGGATGAACTCCACAAGACGAGCCTTCCTGGAGTCACTTTTAAGCAATGGTGGAA
CAACCAAATCAGCCGAGGCAACGTGAAGCCACATTATAGAACTGAGGGGCACTTCATGGAGTTTACCAGAGATAC
TGCGGCATCGGTTGCCAGCGAGATATCACACTCACCCGCAAGAGATTTTCTTGTGAGAGGTGCTGTTGGATCTGG
AAAATCCACAGGACTTCCATACCATTTATCAAAGAGAGGGAGAGTGTTAATGCTTGAGCCTACCAGACCACTCAC
AGATAACGTGCACAAGCAACTGAGAAGTGAACCATTTAACTGCTTCCCAACTTTGAGGATGAGAGGGAAGTCAAC
TTTTGGGTCATCACCGATTACAGTCATGACTAGTGGATTCGCTTTACACCATTTTGCACGAAACATAGCTGAGGT
AAAAACATACGATTTTGTCATAATTGATGAATGTCATGTGAATGATGCTTCTGCTATAGCGTTTAGGAATCTACT
GTTTGAACATGAATTTGAAGGAAAAGTCCTCAAAGTGTCAGCCACACCACCAGGTAGAGAAGTTGAATTCACAAC
TCAGTTTCCCGTGAAACTCAAGATAGAAGAGGCTCTTAGCTTTCAGGAATTTGTAAGTTTACAAGGGACAGGTGC

CAACGCCGATGTGATTAGTTGTGGCGACAACATACTAGTATATGTTGCTAGCTACAATGATGTTGATAGTCTTGG
CAAGCTCCTTGTGCAAAAGGGATACAAAGTGTCGAAGATTGATGGAAGAACAATGAAGAGTGGAGGAACTGAAAT
AATCACTGAAGGTACTTCAGTGAAAAAGCATTTCATAGTCGCAACTAATATTATTGAGAATGGTGTAACCATTGA
CATTGATGTAGTTGTGGATTTTGGGACTAAGGTTGTACCAGTTTTGGATGTGGACAATAGAGCGGTGCAGTACAA
CAAAACTGTGGTGAGTTATGGGGAGCGCATCCAAAGACTCGGTAGAGTTGGGCGACACAAGGAAGGAGTAGCACT
TCGAATTGGCCAAACAAATAAAACACTGGTTGAAATTCCAGAAATGGTTGCCACTGAAGCTGCCTTTCTATGCTT
CATGTACAATTTGCCAGTGACAACACAGAGTGTTTCAACCACACTGCTGGAAAATGCCACATTATTACAAGCTAG
AACTATGGCACAGTTTGAGCTATCATATTTTTACACAATTAATTTTGTGCGATTTGATGGTAGTATGCATCCAGT
CATACATGACAAGCTGAAGCGCTTTAAGCTACACACTTGTGAGACATTCCTCAATAAGTTGGCGATCCCAAATAA
AGGCTTATCCTCTTGGCTTACGAGTGGAGAGTATAAGCGACTTGGTTACATAGCAGAGGATGCTGGCATAAGAAT
CCCATTCGTGTGCAAAGAAATTCCAGACTCCTTGCATGAGGAAATTTGGCACATTGTAGTCGCCCATAAAGGTGA
CTCGGGTATTGGGAGGCTCACTAGCGTACAGGCAGCAAAGGTTGTTTATACTCTGCAAACGGATGTGCACTCAAT
TGCGAGGACTCTAGCATGCATCAATAGACTCATAGCACATGAACAAATGAAGCAGAGTCATTTTGAAGCCGCAAC
TGGGAGAGCATTTTCCTTCACAAATTACTCAATACAAAGCATATTTGACACGCTGAAAGCAAATTATGCTACAAA
GCATACGAAAGAAAATATTGCAGTGCTTCAGCAGGCAAAAGATCAATTGCTAGAGTTTTCGAACCTAGCAAAGGA
TCAAGATGTCACGGGTATCATCCAAGACTTCAATCACCTGGAAACTATCTATCTCCAATCAGATAGCGAAGTGGC
TAAGCATCTGAAGCTTAAAAGTCACTGGAATAAAAGCCAAATCACTAGGGACATCATAATAGCTTTGTCTGTGTT
AATTGGTGGTGGATGGATGCTTGCAACGTACTTCAAGGACAAGTTCAATGAACCAGTCTATTTCCAAGGGAAGAA
GAATCAGAAGCACAAGCTTAAGATGAGAGAGGCGCGTGGGGCTAGAGGGCAATATGAGGTTGCAGCGGAGCCAGA
GGCGCTAGAACATTACTTTGGAAGCGCATATAATAACAAAGGAAAGCGCAAGGGCACCACGAGAGGAATGGGTGC
AAAGTCTCGGAAATTCATAAACATGTATGGGTTTGATCCAACTGATTTTTCATACATTAGGTTTGTGGATCCATT
GACAGGTCACACTATTGATGAGTCCACAAACGCACCTATTGATTTAGTGCAGCATGAGTTTGGAAAGGTTAGAAC
ACGCATGTTAATTGACGATGAGATAGAGCCTCAAAGTCTTAGCACCCACACCACAATCCATGCTTATTTGGTGAA
TAGTGGCACGAAGAAAGTTCTTAAGGTTGATTTAACACCACACTCGTCGCTACGTGCGAGTGAGAAATCAACAGC
AATAATGGGATTTCCTGAAAGGGAGAATGAATTGCGTCAAACCGGCATGGCAGTGCCAGTGGCTTATGATCAATT
GCCACCAAAGAGTGAGGACTTGACGTTTGAAGGAGAAAGCTTGTTTAAGGGACCACGTGATTACAACCCGATATC
GAGCACCATTTGTCACTTGACGAATGAATCTGATGGGCACACAACATCGTTGTATGGTATTGGATTTGGTCCCTT
CATCATTACAAACAAGCACTTGTTTAGAAGAAATAATGGAACACTGTTGGTCCAATCACTACATGGTGTATTCAA
GGTCAAGAACACCACGACTTTGCAACAACACCTCATTGATGGGAGGGACATGATAATTATTCGCATGCCTAAGGA
TTTCCCACCATTTCCTCAAAAGCTGAAATTTAGAGAGCCACAAAGGGAAGAGCGCATATGTCTTGTGACAACCAA
CTTCCAAACTAAGAGCATGTCTAGCATGGTGTCAGACACTAGTTGCACATTCCCTTCATCTGATGGCATATTCTG
GAAGCATTGGATTCAAACCAAGGATGGGCAGTGTGGCAGTCCATTAGTATCAACTAGAGATGGGTTCATTGTTGG
TATACACTCAGCATCGAATTTCACCAACACAAACAATTATTTCACAAGCGTGCCGAAAAACTTCATGGAATTGTT
GACAAATCAGGAGGCGCAGCAGTGGGTTAGTGGTTGGCGATTAAATGCTGACTCAGTATTGTGGGGGGGCCATAA
AGTTTTCATGAGCAAACCTGAAGAGCCTTTTCAGCCAGTTAAGGAAGCGACTCAACTCATGAGTGAATTGGTGTA
CTCGCAAGGGGAGAAGAGGAAATGGGTCGTGGAAGCACTGTCAGGGAACTTGAGGCCAGTGGCTGAGTGTCCCAG
TCAGTTAGTCACAAAGCATGTGGTTAAAGGAAAGTGTCCCCTCTTTGAGCTCTACTTGCAGTTGAATCCAGAAAA
GGAAGCATATTTTAAACCGATGATGGGAGCATATAAGCCAAGTCGACTTAATAGAGAGGCGTTCCTCAAGGACAT
TCTAAAATATGCTAGTGAAATTGAGATTGGGAATGTGGATTGTGACTTGCTGGAGCTTGCAATAAGCATGCTCAT
CACAAAGCTCAAGGCGTTAGGATTCCCAACTGTGAACTACATCACTGACCCAGAGGAAATTTTTAGTGCATTGAA
TATGAAAGCAGCTATGGGAGCACTATACAAAGGCAAGAAGAAAGAAGCTCTCAGCGAGCTCACACTAGATGAGCA
GGAGGCAATGCTCAAAGCAAGTTGCCTGCGACTGTATACGGGAAAGCTGGGAATTTGGAATGGCTCATTGAAAGC
AGAGTTGCGTCCAATTGAGAAGGTTGAAAACAACAAAACGCGAACTTTCACAGCAGCACCAATAGACACTCTTCT
TGCTGGTAAAGTTTGCGTGGATGATTTCAACAATCAATTTTATGATCTCAACATAAAGGCACCATGGACAGTTGG
TATGACTAAGTTTTATCAGGGGTGGAATGAATTGATGGAGGCTTTACCAAGTGGGTGGGTGTATTGTGACGCTGA
TGGTTCGCAATTCGACAGTTCCTTGACTCCATTCCTCATTAATGCTGTATTGAAAGTGCGACTTGCCTTCATGGA
GGAATGGGATATTGGTGAGCAAATGCTGCGAAATTTGTACACTGAGATAGTGTATACACCAATCCTCACACCGGA
TGGTACTATCATTAAGAAGCATAAAGGCAACAATAGCGGGCAACCTTCAACAGTGGTGGACAACACACTCATGGT
CATTATTGCAATGTTATACACATGTGAGAAGTGTGGAATCAACAAGGAAGAGATTGTGTATTACGTCAATGGCGA
TGACCTATTGATTGCCATTCACCCAGATAAAGCTGAGAGGTTGAGTGGATTCAAAGAATCTTTCGGAGAGTTGGG
CCTGAAATATGAATTTGACTGCACCACCAGGGACAAGACACAGTTGTGGTTCATGTCACACAGGGCTTTGGAGAG
GGATGGCATGTATATACCAAAGCTAGAAGAAGAAAGGATTGTTTCTATTTTGGAATGGGACAGATCCAAAGAGCC
GTCACATAGGCTTGAAGCCATCTGTGCATCAATGATCGAAGCATGGGGTTATGACAAGCTGGTTGAAGAAATCCG
CAATTTCTATGCATGGGTTTTGGAACAAGCGCCGTATTCACAGCTTGCAGAAGAAGGAAAGGCGCCATATCTGGC
TGAGACTGCGCTTAAGTTTTTGTACACATCTCAGCACGGAACAAACTCTGAGATAGAAGAGTATTTAAAAGTGTT
GTATGATTACGATATTCCAACGACTGAGAATCTTTATTTTCAG**TCAGGTACAGTAATTTCTACTAAGTGTAGATG**
CAAGAAGACCAGGCTCGAAAGCACTACAGAGAACCTCTACTTTCAAAGTGGCACTGTGGGTGCTGGTGTTGACGC
TGGTAAGAAGAAAGATCAAAAGGATGATAAAGTCGCTGAGCAGGCTTCAAAGGATAGGGATGTTAATGCTGGAAC
TTCAGGAACATTCTCAGTTCCACGAATAAATGCTATGGCCACAAAACTTCAATATCCAAGGATGAGGGGAGAGGT
GGTTGTAAACTTGAATCACCTTTTAGGATACAAGCCACAGCAAATTGATTTGTCAAATGCTCGAGCCACACATGA
GCAGTTTGCCGCGTGGCATCAGGCAGTGATGACAGCCTATGGAGTGAATGAAGAGCAAATGAAAATATTGCTAAA

TGGATTTATGGTGTGGTGCATAGAAAATGGGACTTCCCCAAATTTGAACGGAACTTGGGTTATGATGGATGGTGA
GGAGCAAGTTTCATACCCGCTGAAACCAATGGTTGAAAACGCGCAGCCAACACTGAGGCAAATTATGACACACTT
CAGTGACCTGGCTGAAGCGTATATTGAGATGAGGAATAGGGAGCGACCATACATGCCTAGGTATGGTCTACAGAG
AAACATTACAGACATGAGTTTGTCACGCTATGCGTTCGACTTCTATGAGCTAACTTCAAAAACACCTGTTAGAGC
GAGGGAGGCGCATATGCAAATGAAAGCTGCTGCAGTACGAAACAGTGGAACTAGGTTATTTGGTCTTGATGGCAA
CGTGGGTACTGCAGAGGAAGACACTGAACGGCACACAGCGCACGATGTGAACCGTAACATGCACACACTATTAGG
GGTCCGCCAGTGATAGTTTCTGCGTGTCTTTGCTTTCCGCTTTTAAGCTTATTGTAATATATATGAATAGCTATT
CACAGTGGGACTTGGTCTTGTGTTGAATGGTATCTTATATGTTTTAATATGTCTTATTAGTCTCATTACTTAGGC
GAACGACAAAGTGAGGTCACCTCGGTCTAATTCTCCTATGTAGTGCGAGAAAAAAAAAAAAAAAAAAAAAAAAAA
AAAAAAAAAAAAAAAAAA

TEV-DQ986288 (G273A, A1119G) with a cDNA corresponding to crRNA-3 inserted between the NIb and CP cistrons. crRNA-3 cDNA is flanked by sequences (green) to complement the split NIb/CP **NIaPro-mediated cleavage site**. A **G** extra nucleotide (blue background) was added to maintain the open reading frame. In the crRNA-3 cDNA, **LbCas12a direct repeat** and **protospacer** are highlighted on yellow and green backgrounds, respectively.

>TuMV-crRNA-3
AAAAAATATAAAAACTCAACACAACATACACAAAACGATCAAAGCAAACACAAATCTCTCGAAGCATTCAAGCAA
CCAAAGATTTTCAAATCTTCCATCGTTTTCAAAGCAACCAACAACAACGCAAGCAATGGCAGCAGTTACATTTGC
AACCGCAATCACTAACACCACCGCAAGCAAACCAGCACTCACCGGAATGATACAGTTTGGGAATTTCCCACCAGT
GCCATTGCGATCCACCACCGTTACCACAGTCGCCACTTCAGTGGCGCAACCTAAACTGCACACAGTGCAGTTTGG
AAGCCTTGACCCAGTAGTCGTCAAGAGTGGAGCAGGGTCCTTTGCTAAGGCAACACGCCAGCAGCCTAACGTTGA
AATAGACGTTAGCCTCAGTGAAGCCGCAGCTCTGGAGGTTGCGAAACCTAGACCAAACGCCGTGTTGAGGATGCA
CGAGGAAGCAAACAAGGAGAGGGCACTCTTTTTGGACTGGGAGGCTAGTTTGAAGAGAAGCTCATATGGAATTGC
TGAGAACGAGAAAGTTGTAATGACAACTCGTGGCGTCAGCAAGATAGTGCCCAGAAGTTCAAGGGCAATGAAGCA
AAAGCGCGCAAGGGAAAGACGTAGAGCGCAACAACCAATCATACTAAAGTGGGAGCCTAAATTGAGCGGGATCTC
AATTGGAGGAGGGCTCTCCGCGAGCGCGATCGAAGTGGAAGAAGCCCGCACAAAGTGGCCGCTTCACAAGACACC
GTCAATGAAGAGGAAGACGGTGCACAGAAGATGCAAGATGAATGATCAAGGAATTGACATGTTAATGCGTTCTTT
GATTAAAATCTTTAAGGCGAAGAGTGCGAATATTGAGTTCATTGGAAGGAAGTCCATTAAAGTGGATTTCGTAAA
GAAAGAGCAAACGAAGTTTGCCAGAGTACAAGTAGTGCATTTGCTCGGAAAGCGAGCACAACGCGACCTGTTGAC
TGGTGCAGAAGAGAACCACTTCATTGACACCCTAAGCAACTACTCAGGAAATAGGAAAATCATAAACCCAGGAGT
GGTGTGCGCTGGTTGGAGTGGCATTGTTATCAGAAACGGCATTTTGACTCAGAAGCAGAGCAGAAGTCCTTCGCA
AGCCTTTGTGATTCGAGGGGAACATGAAGGGAAGTTGTATGACGCCAGAGTTAAGGTCACAAAGGTGATGAGCCA
CAAAATCATACACTATAGTGCGGCAGGAGCCAATTTTTGGAAAGGATTTGACGGGTGTTTCCTCGCATATCGAGG
TGACAACCGTGAACATACATGTTATACGGGATTGGATGTCACCGAGTGTGGGGAGGTTGCAGCGCTAATGTGTTT
AGCCATGTTCCCATGTGGAAAAATAACTTGTCCTGACTGCGTAACAGACAGCGAGTTGTCTCAAGGGCAGGCAAG
TGGACCATCCATAAAACACAAGTTAGCACAACTACGTGAGGTTATCAAGTCCAGTTATCCACGGTTTAAACATGC
AGTGCAAATACTCGACAGATATGAGCAGTCATTGAGCAGTGCAAATGAGAACTATCAGGATTTTGCGGAAATTCA
AAGCATAAGTGATGGAACAGACAAAGCAGCGTTTCCGCACATTAATAAACTAAATGCAATATTAATTAAGGGAGC
GACAGCAACTGGAGAGGAATTCTCGCAAGCAACAAAGTACTTGCTTGAGATAGCCCGGTATCTGAAGAATAGAAC
CGAGAATATTGAAAAGGGTTCGCTTAAATCCTTCCGCAATAAGATCTCTCAAAAGGCGCACATAAACCCAACATT
GATGTGTGACAATCAGCTCGACAGGAACGGCAACTTCATATGGGGTGAGAGAGGTTATCACGCGAAGCGGTTCTT
TAGTAACTACTTCGAAATAATCGATCCAAAGAAAGGCTACACTCAATACGAGACGAGAATAGTGCCAAACGGATC
ACGAAAGCTCGCAATTGGCAAACTCATAGTCCCAACGAACTTCGAAGTCCTGAGGGAGCAGATGAAAGGCGAGCC
CATAGAACCACATCCAATCACAGTTGAGTGCGTAAGCAAGCTACAGGGCGACTTCGTTCATGCGTGCTGTTGTGT
TACAACGGAGTCAGGCGACCCAGTCTTGTCTGAAATTAAGATGCCAACTAAGCACCACCTGGTAATTGGTAATAG
CGGCGACCCAAAGTACATCGATCTCCCTGAAATTGAGGAAAACAAGATGTACATAGCGAAAGAAGGCTATTGCTA
CATTAATATCTTCTTAGCTATGCTAGTTAATGTTAAAGAGTCGCAAGCAAAAGAATTTACAAAAGTGGTCAGAGA
CAAACTGGTTGGAGAACTTGGCAAATGGCCCACTTTGTTGGATGTAGCGACTGCTTGTTACTTCCTAAAAGTGTT
TTACCCTGACGTCGCCAACGCTGAATTGCCACGCATGCTAGTGGACCACAAAACGAAGATTATCCACGTAGTCGA
CTCGTATGGGTCACTGTCAACAGGATATCACATCCTTAAGACGAACACCGTGGAACAACTTATCAAATTCACTAG
GTGCAATTTAGAATCGAGTTTGAAGCACTACCGTGTTGGAGGCACCGCGTGGGAAGGAGCCCATGGGTTTAATAA
CATAGACGACCCGCAATGGTGCATTAAAAGGCTCATTCAAGGAGTGTACAGGCCAAAGAAGTTGAGGGAAGACAT
GCTGACAAACCCTTTTCTAACACTGTACGCCTTGTTATCACCAGGAGTGATTCTAGCATTCTATAACAGTGGCTC
ACTGGAGTACCTCATGAATCACTACATTAGAGCAGATAGCAACGTAGCTGTTCTGTTAGTAGTGCTAAAATCTCT
AGCAAAGAAAGTGTCAACCAGCCAGAGTGTGTTAGCTCAACTCCAAATAATCGAGCGAAGTCTGCCGGAACTCGT
GGAAGCCAGGGCTAATATAACTGGACCAGATGAAGCAGCCTCCCAGGCATGCAATAGATTCCTGGGCATGCTCAT
CCATATGGCAGAACCAAATAACGAACTAGCAGATGGCGGATATACAATCCTGAGGGATCATAGTATCTCCATCTT
AGAAAAAAGTTATCTGCAAATCTTGGACGAAGCATGGAGCGAATTAAGCTGGTCGGAGCGTTGTGCTATAAAATA

CTACTCGTCAAAGCAAGCAATCTTTTCACAGAAAGATTTGCAAATGCGAAGCGACGTCGATTTAGGCGGCAGATA
CAGCGAGTCAGTCACGTCCTCCTACGAGTGGGGTAAACAACGCGTGAAAAGCGTGTATTCTAATGCATGTAATAA
AGTACGTAGTAGTGTATCTTGGACTAGTAGTAAAATTTCAAGTAGTGTTTGTAAAACAATCAACTATTTAGTACC
AGATGTGTTCAAATTCATTAATGTATTAGTTTGCATTAGTTTATTAGTCACAATAGCTGCTGAAGCAAATCGTAT
TGTCACCACACAGAGGAGACTCAAACTGGACATCGAGGAAACAGAGCGCAAGAAGATTGAATGGGAACTCGCATT
CCATCATGCTATTCTAACGCAAAGTGCAGGGCAGCACCCAACACTAGATGAGTTCACAGCATACATTGGTGAGAA
AGCACCTCATCTTAATGAGCATATTGAACCTGAGGAGAAGGCAGTAGTGCACCAAGCGAAAAGACAATCTGAGCA
GGAGCTCGAACGAGTGATAGCTTTTATCGCCTTAGTTCTCATGATGTTTGACGCAGAACGCAGTGATTGCGTCAC
GAAGATCCTCAACAAGCTTAAGGGGTTAGTGTCAACTGTGGAACCTACAGTTTACCACCAAACACTTAATGATAT
CGAAGATGACTTGAGTGAGAGGAATCTCTTTGTCGACTTTGAACTTAGTAGTGATGGTGAAATTCTCCAGCAGCT
CCCAGCTGAGAAAACGTTTGCTTCGTGGTGGAATCATCAATTGAGTAGAGGATTCACAATCCCGCACTACAGAAC
AGAAGGAAAGTTTATGACCTTCACCAGGGCAACCGCCACAGAAGTTGCGGGTAAGATAGCACATGAGAGTGATAG
AGATATACTCCTCATGGGAGCGGTGGGATCAGGCAAGTCAACTGGTTTACCATATCACCTTTCCAGAAAGGGGAA
TGTATTGCTTCTCGAACCCACTCGACCACTCGCAGAAAACGTGCACAAGCAATTGTCGCAGGCACCATTTCATCA
GAACACGACTCTTCGAATGCGTGGGTTGACATCGTTTGGATCGGCACCGATCTCGGTGATGACTAGTGGTTTCGC
ACTTAACTATTTCGCAAACAATCGAACGAGGATTGAAGAGTTTGACTTTGTCATATTCGATGAATGTCATGTTCA
CGACGCTAATGCAATGGCGATGAGATGTTTGCTACATGAGTGCGACTACTCCGGCAAGGTCATAAAGGTTTCCGC
CACACCACCAGGTCGCGAGGTTGAATTCTCCACTCAATATCCTGTGACAATTAGCACAGAAGACACATTGTCATT
TCAAGACTTTGTGAATGCACAGGGTAGTGGAAGTAACTGCGACGTGATCTCAAAAGGAGACAACATCCTCGTATA
CGTAGCAAGCTACAACGAGGTGGACACACTTTCAAAGCTTCTAGTCGAGCGTGATTTCAAAGTTACGAAAGTTGA
CGGAAGAACAATGAAAGTCGGAAATATTGAAATCACCACGAGCGGGACACCTAGTAGGAAACACTTCATAGTCGC
AACCAACATTATTGAGAACGGGGTCACCCTGGATATTGATGTTGTTGCCGACTTTGGAACAAAAGTACTCCCGTA
TCTTGACACGGATAATAGAATGCTTAGTACAACGAAAACAAGCATTAACTATGGGGAGCGAATCCAAAGGCTAGG
GAGAGTTGGAAGACACAAGCCAGGCCACGCTCTGAGAATTGGCCACACGGAGAAAGGGCTGAGTGAAGTTCCAAG
CTGCATTGCAACAGAAGCAGCCCTAAAGTGTTTTACGTATGGACTTCCAGTTATAACCAACAACGTGTCTACAAG
CATTCTAGGCAATGTAACGGTGAAACAGGCACGAACAATGTCTGTGTTTGAAATAACACCGTTCTACACAAGCCA
AGTGGTTAGATATGATGGCTCGATGCATCCTCAGGTCCATGCGCTTTTGAAGAGGTTCAAGCTCAGAGATTCTGA
AATTGTCTTGAACAAATTAGCCATACCTAACCGGGGAGTAAATGCTTGGCTCACAGCTAGTGAGTATGCACGACT
TGGTGCAAGCGTTGAGGACAGGCGTGACGTACGGATCCCTTTCATGTGTCGAGACATCCCAGAGAAACTCCATCT
AGAGATGTGGGATGTGATTGTCAAGTTCAAAGGCGATGCAGGTTTCGGGCGACTTTCAAGCGCCAGCGCAAGCAA
GGTAGCTTACACCTTGCAAACAGACGTCAACTCCATCCAGCGAACAGTCACTATTATAGATACACTAATCGCTGA
GGAGAGAAGGAAGCAGGAGTACTTCAAAACAGTAACCTCCAACTGTGTCTCTTCTTCAAACTTCTCACTACAGAG
CATCACAAATGCCATAAAATCTCGTATAATGAAAGATCACACGTGCGAGAATATATCAGTGCTTGAAGGAGCAAA
GTCACAGTTACTCGAGTTCAGAAACTTAAACGCTGATCACTCGTTCACTACTAAGTCTGACGGGATTTCTCGCCA
TTTTATGAGTGAATATGGAGCACTCGAGGCAGTTCACCACCAAAACACCAACGACATGAGCAAGTTCTTAAAGCT
GAAGGGCAAATGGAACAAAACGCTTATCACGCGTGATGTGTTAGTGATTTGTGGAGTTCTTGGAGGTGGAATATG
GATGATCATCCAACACCTGCGTGCGAAGATCTCCGAACCCGTGACCCACGAGGCAAAAGGCAAGAGGCAGAGACA
GAAGTTGAAGTTTCGCAATGCCCGCGACAACAAAATGGGTAGGGAGGTTTACGGAGAAGACGACGTCATTGAACA
CTTCTTTGGAGATGCCTACACAAAGAAGGGAAAGAGTAAAGGCAGAACTCGTGGCCTCGGCCATAAAAACAGGAA
ATTCATCAATATGTATGGATTCGATCCTGAAGACTTCTCTGCAGTTAGATTCGTAGATCCACTCACTGGGGCAAC
GATAGATGAAAGTCCAATCATGGACATAGCCCTTGTGCAGGAACATTTTGGAAAAATACGGATGAATTTACTTGG
AGAAGACGAACTGGAACCCGATGAGTTGCGAATGAATAAGACGATTCAGGCTTACTACATGAATAACAAAACAGG
TAAAGCCCTGAAAGTGGATTTGACACCACACATACCACTAAAGGTATGTGACCTTCATGCAACAATTGCTGGTTT
CCCGGAACGAGAACACGAACTGAGGCAAACTGGAAAAGCTCAGCCTATAGACTTAAGTGAAGTACCTAAAGCTAA
CACTGAGCTAATTCCAGTTGACCATGAAAGTAGCTCCATGTTTAGAGGGTTGCGCGACTACAACCCAATATCCAA
TAACATTTGTCATCTTACAAATGTGTCAGATGGAGCATCAAATTCGTTGTATGGGGTTGGTTTTGGACCACTCAT
ATTAACGAACCGGCATCTCTTTGAAAGGAATAATGGTGAACTCGTGATAAAGTCAAGACATGGTGAGTTCGTGAT
TAAGAACACGACTCAGCTACACTTGCTACCGATTCCGGACAGGGACCTTCTGCTAATCCGACTACCTAAAGACAT
TCCACCCTTCCCGCAAAAACTGGGATTTAGACAACCTGAGAGGGGTGAGAGGATCTGTATGGTAGGTTCCAACTT
TCAAACAAAGAGCATCACGAGTGTAGTTTCTGAGACTAGCACAATAATGCCAGTGGAGAATAGCCAATTTTGGAA
GCACTGGATTAGCACCAAGGATGGCCAATGTGGAAGCCCGATGGTGAGCACAAAGGATGGAAAAATACTTGGGCT
GCACAGCCTGGCAAATTTCCAAAATTCCATCAACTACTTTGCTGCCTTCCCAGATGATTTTGCTGAGAAGTACCT
CTACACTATTGAGGCACACGAGTGGGTCAAGCATTGGAAGTATAACACCAGCGCAATTAGTTGGGGCTCCCTAAA
TATACAAGCATCGCAGCCAGCGGGCTTGTTTAAAGTGAGCAAACTAATATCAGACCTTGACAGCACAGCGGTTTA
TGCGCAAACACAGCAGAATCGCTGGATGTATGAACAGCTGAATGGGAACTTAAAGGCAATAGCACACTGCCCTAG
TCAACTTGTGACGAAGCACACGGTTAAAGGGAAGTGCCAGATGTTCGACTTGTACCTCAAGTTGCATGATGAAGC
GCGAGAATACTTCCAGCCAATGTTAGGGCAGTACCAGAAGAGTAGACTCAATCGAGAGGCATACGCAAAGGATCT
TCTCAAATATGCAACGCCAATCGAAGCGGGAAATATTGATTGTGATCTGTTTGAAAAGACAGTTGAGACAGTCAT
ATCAGACTTGCGAGGTTATGGTTTTGAGACGTGTAATTACGTCACTGATGAGATCGACGTATTCGAAGCCCTGAA
TATGAAATCCGCAGTCGGAGCATTGTATAAAGGCAAGAAGAAAGATTATTTTGCTGAGTTCACGCCCGAGATGAA
AGAAGAGATACTCAAACAGAGTTGTGAACGCCTCTTCCTTGGGAGGATGGGAGTCTGGAATGGCTCACTAAAGGC

AGAATTGCGACCACTAGAGAAAGTGGAAGCAAACAAAACACGGACGTTTACAGCCGCGCCACTAGATACACTGTT
AGGTGGGAAAGTCTGTGTGGATGACTTCAACAATCAGTTCTATGACCACAACCTTAGAGCTCCGTGGAGCGTAGG
TATGACAAAGTTCTATTGTGGTTGGGATCGCTTGCTAGAGTCACTACCAGATGGTTGGATTTACTGCGATGCTGA
TGGGTCACAGTTCGATAGTTCACTATCGCCATACTTGATTAACGCAGTGCTCAACATCCGCTTGGAATTTATGGA
AGAGTGGGACATAGGGGAAGTAATGCTAAGAAACTTGTATACTGAGATTGTGTATACCCCTATCTCAACGCCAGA
TGGCACACTCGTCAAGAAGTTCAAAGGGAACAATAGCGGACAACCGTCAACAGTCGTGGACAACACGCTCATGGT
CATATTAGCAGTCAACTACTCACTCAGGAAGAGTGGAATTCCAAATGAATTGCGTGATAGCCTCATCAGGTTTTT
CGTCAATGGAGATGACTTGCTGCTAGGCGTACATCCAAAGTACGAGTATGTCCTTGACACTATGGCGGATAATTT
CCGTGAACTGGGCTTGAAGTATACCTTCGATTCAAGAACCAGAGAAAAAGGTGACCTTTGGTTTATGTCGCACCA
AGGACACAAAAGAGAAGGGATCTGGATCCCCAAGCTGGAACCAGAGCGAATAGTATCGATCCTAGAGTGGGATCG
ATCAAAAGAGCCATGCCACCGACTTGAGGCAATCTGCGCAGCAATGATTGAATCGTGGGGATATGATAAGTTGAC
CCATGAGATACGCAAGTTTTATGCATGGATGATCGAACAGGCTCCATATAGCTCTCTAGCACAAGAGGGAAAAGC
TCCTTACATAGCGGAAACGGCACTAAGGAAACTTTACCTTGACAAAGAACCAGCTCAAGAAGATCTTGCTCAATA
CTTACAAGCAATCTTCGAAGATTACGAAGATAGTGCTGAAGTGTGTGTTTATCACCAG**GCAGGAGAGGTAATTTC**
TACTAAGTGTAGATGCAAGAAGACCAGGCTCGAAAGCGCTGAGGTGTGCGTTTACCACCAAGCAGGTGAGACGCT
TGATGCCGAGCTAACAGAAGAGCAGAAGCAGGCTGAAAAGGAGAGAAAGGAGAGAGAGAGATCAGAGAAAGAGCG
AGAAGGGCAAAGACAGCTAGCGCTTAAGAAAGGCAAAAACGCAGCACAAGAAGAGGGCGAACGTGACAATGAAGT
AAACGCCGGAACTTCCGGAACTTTTAGCGTGCCTAGACTCAAAAGTCTTACAAGCAAAATGCGTGTGCCGAAGTA
TGAGAAAAGAGTCGCTCTCAACCTCGATCATTTGATCTTATACACGCCGGAGCAGACAGATTTATCCAACACGCG
TTCAACGCGGAAGCAGTTTGACACTTGGTTTGAAGGTGTCATGGCTGACTATGAGCTAACGGAGGATAAAATGCA
AATAATTCTCAATGGTTTAATGGTCTGGTGCATTGAGAATGGAACCTCCCCGAACATAAACGGAATGTGGGTGAT
GATGGACGGCGATGATCAGGTGGAATTCCCGATCAAACCGCTCATTGACCACGCCAAACCCACATTTAGGCAGAT
AATGGCCCATTTCAGTGACGTAGCTGAAGCGTACATTGAAAAGCGCAACCAAGACCGACCATACATGCCACGATA
TGGTCTTCAGCGCAATTTAACCGACATGAGCTTAGCTCGATACGCGTTTGATTTCTATGAAATGACTTCTAGAAC
TCCAATACGTGCGAGAGAAGCACACATCCAGATGAAAGCAGCAGCACTGCGTGGCGCAAATAACAATTTGTTCGG
CTTGGATGGAAACGTTGGTACAACGGTAGAGAACACGGAGAGGCACACGACCGAGGACGTTAATCGGAACATGCA
TAACTTACTTGGCGTTAAGGGGTTATGAAGTTGTATGCTGGTAGACTATAAGTATTTAAGTTTACTCGTTAGTAT
TCTCGCTTATGGGAAATATGTAAGTTTGTTAAAGCAGCCAGTGTGACTTTGTCATGTGTGTTGTTGTTACTTTCT
ATATTTTCGCCGAACATTTTATTGGTGTTAGCGCATGGGGTGAGGTTCGTCCTCGATTGCCTTAACATTTGATAG
GATGCAAGGGACAAAAAAAAAAAAAAAAAAAAAAAAAAAAAAAAAAAAAAAAAAAAAAAAA

TuMV-JPN1 with a cDNA corresponding to crRNA-3 inserted between the NIb and CP cistrons. crRNA-3 cDNA is flanked by sequences (green) to complement the split NIb/CP **NIaPro-mediated cleavage site**. A **G** extra nucleotide (blue background) was added to maintain the open reading frame. In the crRNA-3 cDNA, **LbCas12a direct repeat** and **protospacer** are highlighted on yellow and green backgrounds, respectively.

>LMV-crRNA-3
AAAATAAAACAACCCAACACAACTCAAGAAAATTCATACAAACAAACAAATTCGTATTTTCAAGCAATTCACTTT
CAAGCAATTACAACATTTTCAATCACAAATGGCAACTCTAGATAACTGCACTCAAGTACACCACATGTTCGCCTA
CAATCGTGAACACGGAACGAACTACACGAGAAACCATTTCAGAAGATACTTAGCAGCCCAGCGAATAGGTTTCTA
TTACGATTGGGACGATGATGTCTATGAATGTCCAACGTGTGAAGCTATATACCATTCTTTGGACGAAATAAAGAA
CTGGCACGAGTGCGATCCACCAGCATTCGATCTTAACGATTTCATAACTGATGCTAGGCTGAAATCAGCACCAGT
TCCAGATCTCGGACCGGTGATCATCGAAACCCCACAAGCGGAGGAAAAACAAGAGCTTAATTTCTTCGCTGCAAC
CCCGGCACCTGAAGTTTCACAATGGAAATGTAGAGGGTTGCAATTTGGTTCATTTACTGAACTTGAAACATCTGA
ACCAGTTGTTTCGGTACCAGAACCCAAGTGTGAAGAGCCGGTTAGGACCATCGCAAAGCCAGAAGAGTCAATCGA
ACAAGAGACCTGTGGAGACGGAAAAAGACTTCTTCAGGCGCAGATGGAAGTCGACAAAGCCGAACAAGATCTTGC
ATTCGCTTGCCTGAACGTTAGCCTAAAACCTAGGTTGGAGGGCAGAACTACTGCAACTATTGCAAGACGCAGGGA
TGGATGTTTGGTTTATAAAACCAAATCAAGCTGGTCGCAGAGGAAGAGAACAAAGAAGATCCTTAAGGGTGATAC
CCTAGCTTGTAAAAACCCATACACTCCAGCGGTTGTTGACAAAATCTCAATCGCAGGAGGCTCATCAGCCAGTGT
GATGCACGAACAACAGAAACCTAAAATTCTGCACACTACACCATCAAGAAAGGTGGCCACGCATTATAAACGGAC
GGTGATGAACCAACAGACACTCGCAGCTCTTATAGATCAAGTTGGCACGATTCTACTAAACGCAGGAAAAGAGTT
TGAAGTGGTGGGCCGCAGGAAGCAGAAAGTAACTGGCAAGGGAACTAGACATAACGGTGTGAGGCTGGTAAAGCT
TAAAACTGCACATGAGGAAGGGCATCGGAGAAGAGTAGACATACGCATCCCCAATAGCCTGCGCTCAATTGTTAT
GCGTATTTCAGCTCGGGGCGGTTGGCACAGAACATGGAAAGATTCAGAACTATCCCCAGGATCAAGCGGTTACGT
ATTGAACTCGTCAAAAATCATAGGAAAATTTGGTTTAAGGCGACACAGCATATTCATTGTTAGGGGGAGGGTAGA
TGGTGAAGTTATAGACTCGCAAAGTAAGGTCACACATTCCATCACGCACCGTATGGTTCAATACAGCGACGTCGC
ACGAAACTTCTGGAACGGGTACTCAACTTGTTTCATGCACAATACCCCAAAGGACATACTCCATACCTGCACATC
AGATTTTGATGTTAAAGAATGTGGCACTGTCGCAGCACTTTTAACTCAAACACTGTTTCAATTTGGGAAAATCAC
CTGTGAAAAGTGCGCAATCGAGTACAAAAATCTAACGCGAGACGAGCTCGCTACGCGTGTTAATAAGGAGATTGA

TGGAACCATAATCAGCATTCAAACTCAGCATCCACGCTTCGTGCATGTACTCAATTTTCTCAGGTTAATTAAACA
AGTACTCAATGCTAAGAATGAGAACTTTGGAGCATTTCAAGAGACGGAGAGGATAATTGGGGATCGAATGGATGC
ACCTTTCTCACACGTAAATAAGCTGAATGCTATCGTCATTAAAGGTAATCAAGCAACATCTGATGAGATGGCACA
AGCATCGAACCATGTCCTCGAAATCGCACGATATCTCAAGAACCGAACTGAGAACATCCAAAAGGGCTCACTAAA
GTCATTCAGGAACAAAATCTCCGGTAAGGCACACTTAAATCCGAGTCTTATGTGTGACAATCAACTTGATAAGAA
CGGCGGGTTTGAATGGGGGCAGCGAAGTTACCATGCTAAAAGGTTTTTCGACGGATACTTCGAAACCATTGACCC
ATCTGATGGCTATAGCAAATACACCATAAGACGCAATCCAAATGGACATCGAAAGTTGGCAATTGGTAATTTGAT
CGTCTCCACGAACTTTGAATCACATAGAAGAAGCATGATTGGAGAATCAATCGAAGACCCTGGTCTCACTAACCA
GTGCGTGAGCAAAGAGGGAGATGCCTTCATCTATCCATGCTGCTGTGTAACAGATGAATATGGTAAACCAACATT
ATCTGAGATTAAAATGCCTACAAAGCATCATCTAGTCCTAGGAAATGCCGGTGACCCCAAATATGTGGATTTACC
AAAGGAAGCGGAAGGAAAGATGTTCGTAGCAAAAGACGGATATTGTTACATAAACATCTTCTTGGCTATGCTTGT
TGACGTCCCAGAGGATCAAGCTAAAGATTTCACGAAGATGGCACGCGAGATAGCAGTGAAACAGCTCGGGGAGTG
GCCCTCAATGATGGATGTAGCAACGGCTTGTAATATATTAGCTACATTTCATCCAGACACTCGAAGATCGGAGTT
ACCTCGAATCTTAGTCGACCACGCAACGAAAACATTCCATGTAATTGATTCATATGGTTCAATCACGACTGGATT
CCATATTCTGAAAGCCAACACCGTGACGCAACTCGTCAAGTTCGCGCATGAGTCACTAGAATCTGAGATGCAACA
CTACAGAGTAGGGGGGGAACCAGATAAAGCACCCAGGAAACCAGCTGGCAGTGTCCCAACTCTAGGAATTTCAGA
CCTCAGGAACCTTGGAGTGGAATCAGAAAACGAAGAGCACTCAATCCGGCCAAATCTCCAAAGGTTGATCAAGGC
GATTTACAGACCCCGAATGATGCGCAGCCTCTTAACAGAGGAACCATACTTGTTAATCCTAAGTATTGTGTCCCC
TGGCGTTCTGATGGCACTCTACAACAGTGGTTCCTTGGAGCGGACAATGCATGAATTCTTGCAAACTGACCAGAG
ACTGAGCGCCACCGCCCAAATCCTAAAACATCTAGCAAAGAAAGTTTCACTCGCAAAGACACTTACGATTCAGAA
TGCTATCTTGGAGGGTGGAGCAGGATCACTGAATGAAATTCTTGACGCGCCCGCGGGACGATCCCTATCGTATAG
ACTAGCAAAGCAGACGGTGGAGGTGATGATGGCGCGGAGCGACATGGATAAGGAACTGGTGGACGTTGGATTTAG
CGTTCTTAGGGATCAGAAGAATGAACTCATAGAAAAAAGTTATCTCATGGATTTGGAGGACTCGTGGCACGCACT
ACCATTGTGTGGAAAATTATCGGCAATGCGAGCCTTGCGGCGATGGCGGGACACCTCTACTCCCGAAGCAATCCC
GACAGGTGCCGCAGATTTGAAAGGCAGATACAGTATCTCGGTTGGATCTGTTTCCAAAAGCGCGATCTTACACCT
AAAGGGAATTTGTTCAGGCGCAGTAAAGAGAGTTAGAGACAAGTGGGTCGGAGTGCAAGTGCAAGGCGTAAAATG
GTTAGCTAAATCAGTACACTACATGATACCAGAACTAATGAACGTACTGAATGTTGGAACTCTCCTCCTAACGCT
GATATCGCTAGGCGTAGCATTCCGGAATTTGACTGGCCAGTTCAAAGAGATGAAACACAAAGAAACGCTGGCAAA
GGAGGAGGAACTACGTAAGCGCATACGCACCTACAATAGCACTTACTACGAAATCCATGGAAAGCACGCTGATGC
CAAACAAATCACTAAATTCATAACACACCATGATCCAAAACTACTGGAAGTAGTTGAGTTTTACGAAGGTCCTGA
AGAAGAAGAAGTGGAACATCAAGCAAAGCGAGAGGATCAAGCTAATCTCGAACGCATTATCGCTTTTACAGCCTT
AGTGATGATGATGTTTGACAGCGAGAGAAGTGATTGTGTGTACAGGAGTCTATCAAAGCTTAAATCATTAGTATC
AACGTGTGAGGATAATGTACGCCACCAGAGCGTTGATGAGATCATTGACCTGTTCGACGAGAAGAAAGAAACTAT
AGATTTCGAGATTGAAGGAAAGGAACTTTACTCTTCTCGCGTGGTGGATTCGACATTCAGCAAGTGGTGGGACAA
TCAATTGGCACGGGGCAACACGATGGCACATTACAGAACAGAGGGACACTTCATGACGTTCACTCGTGAAACAGC
TGCGAGCGTGGCAGCCGAAATAGCACATAACGAGTATAGAGATATTCTCTTGCAAGGTGGTGTCGGCTCCGGCAA
GTCTACAGGTCTCCCGTTCCATTTGCACAAGAAGGGAGGTGTACTACTCATCGAACCAACTCGTCCGTTAGCTCA
AAATGTATACAAGCAGCTTGGAAGTAGCCCTTTTCACCTGTCACCAAATTTGCGTATGCGAGGTTCTTGCAAGTT
TGGATCTAGTCAGGTGACTGTGTCCACGAGTGGTTATGCCTTACATTTCATAGCGAACAATGCGCAAAGTCTTAA
GGCATATGACTTCATCATTTTTGATGAATGTCACGTATTAGATGCCAGTGCAATGGCGTTTAGATGCTTACTGCA
GGAGTTCGAGTATCAAGGGAAGATCATAAAGGTATCAGCCACGCCACCGGGAAGAAAGCTTGACTTCAAACCAAT
GCACATGGTCGATATTGCTACAGAAAATGAACTATCGATACAGCAATTCGTCCAAGGTCAAGGAACTGGAGTAAA
CTGTGATGCAACAAAGAAAGGAGACAATATCTTGGTCTACGTCTCAAGTTACAACGAAGTGGACATGTTGAGCAA
AATGTTAAACGACAAAGGTTACAAAGTGACAAAGGTTGACGGTAGGACGATGAAGTTAGGGAGTGTGGAAGTAGA
AACAGTAGGCACCCCACAGCGGAAACACTTTGTAGTGGCAACCAACATCATAGAAAATGGCGTTACATTGGATGT
CGATGTCGTGGTGGATTTCGGGCAGAAGGTAGTTCCCATTCTTGACAGCGAGCACCGAATGATCCGGTATACAAA
GAAAAGTATCACATATGGTGAGCGAATCCAAAGAGTGGGAAGGGTCGGGCGAAACAAAGCTGGTTCTGCCATCCG
GATAGGGAGCACTGAGATGGGGACGGAAGAAATACCGGCATCAATCGCAACAGAAGCAGCCTTTTTGTGTTTCAC
ATACGGATTTCCTGTCATGACAAGCAACGTGAGCACAAGTGTACTTGGCAATTGCACAGTTAGACAAGCACGGAC
GATGCAAAAGTTTGAGCTGTCCCCCTTCTTTATGGTTGACTTGGTACACCACGACGGTACAATGCACCCAGCAAT
TAACAGCCTCTTGAAACAGTTCAAATTGAAGGAATCAGACATCACACTAAGCACGCTGGCAATACCGAATGCAGT
AACCACATTCTGGAAAAGCGCTCGAGAGTATAACTCCTTAGGTGCTCGCACAACAATCGATGACGCAGCTAAAAT
ACCATTTATGATCAAGGATGTTCCAGAACACTTGCAGGAAAAGCTTTGGGAGACAATCCAGCAGTATAAGGGTGA
TGCAGGTTTTGGAAGATGCACATCAGCTAACGCGTGCAAAATAGCATACACACTCTCTGTGAGTCCCTTTATGAT
CCCAGCAACAATCAACAAGATTGATGCTCTGATGGCCGAAGAACGACAGAAGATGGAATATTTCCAAACAGTCAC
AGCTAATACATGTACAATCTCAAACTTCTCCATTAGTAGCATTGGCGACATGATCAGGTCGAGATACTCAACAAA
CCATTCTAGAGAAAACCTGCAGAAATTACAAACGGTCAGAGACACAATCATCAACTTTGAGTGCCAGGCTGGAAC
TAGTGATGGGGGTACCTTCGACATGGAGACAGCACAGAAATTGGCAGAGGAGTATGGGTGCATCGATGTCATTTA
TCACCAATCGAAAGGAGCTCTTAGCAAACGACTCGGTCTCAAAGGCAGGTGGAATCAGAGCCTTATATGCAAAGA
CCTATTGATTTTCTGCGGCGTAGCCATTGGCGGCACATGGATGATGTTTCAGAGCTTCAAGGACGGGATGGCTGA
CGTAATTCGACACCAAGGCAAAGGTAAACGGCAAAGACAGAAGCTTCGTTATCGACAAGCAAGGGATAATAAGAT

GGGCATTGAAGTTTATGGTGACGATGCGACGATGGAACACTATTTTGGAGCTGCGTACACAGAGAAAGGAAAGAA
ATCCGGAAAGACGAAAGGAATGGGGACGAAAAATCGAAGATTTGTTAACATGTATGGGTACAACCCAGAAGATTA
CTCGTTCATCCGATTTCTGGACCCACTCACAGGGAAAACAATGGATGAACAAGTATTCACTGACATAAGTCTCGT
CCAAGATGCCTTTGGTAAAGAAAGACTCAAACTCCTGTCCGAAGGGGAAATTGAGTCAGAGCACATGCGAAATGG
GATTAGAGCTTATCTTGTCAAGAATCTTACCACAGCAGCTCTCGAAATAGACATGACCCCTCACAACTCTTGCCA
GCTCGGAACCAAGACAAACAACATAGCAGGATTTGTAGACAGGGAGTACGAATTGCGTCAAACCGGGGAAGCCAG
GGTTGTTGCTCCAGCACTGATTCCAAAAGACAATCCAATCACGGACGAGGATATTCCCGTAAAGCATGAAAGCAA
GACATTGTTCAGGGGCTTAAGAGATTATAACCCTATAGCATCAGCAATATGCTTGCTCACTAACGAATCAGACGG
AATGAAAGAGACAATGTACGGCATTGGTTTTGGCAACACAATTATTACAAACCAACACCTATTCAGACGCAACAA
TGGCGTACTAAGGGTTCAGTCGAGACATGGTGAATACGTTCTTCCAAATACAACGCAACTCAAAGTACTTCCTTG
CGAAGGAAGGGACATAATGGTCATCATTCTTACACCAGACTTCCCTCCGTTCCCACAAAAACTGAAATTCCGTCC
ACCGATCAAAGGAGAGAAGATTTGCCTTGTTGGATCCTTATTTCAAGATAAGAGCATAACCAGCACCGTATCCGA
GACGAGCGTGACAACACCCGTGGACAACAGCTTCTTGTGGAAACACTGGATCACTACAAAAGACGGACATTGCGG
GCTTCCACTAGTATCATCAAATGATGGATACATAGTCGGAATCCACAGCGCCACAAGCTCACGGCAAACACAGAA
CTACCATGCAGCGATGCCCGAAGATTTTCATCAAACACATCTTATTGATCCAGTTTCGAAATCGTGGGTGAAACA
TTGGAAGTATAATCCAGATAACATGGTTTGGGGAGGCATAAATCTCATTAATAGCACGCCAAGGGAACCCTTCAA
GATAAACAAATTAGTGACAGACTTATTCGGGGATGCAGTACAGTTTCAGTCCAAACAGGATGAGTGGTTCGCAAG
TCAGTTGAAAGGCAACTTGAAAGCAGTGGGGAAAAGCACAAGCCAACTCGTGACAAAGCACACAGTCAAGGGTAA
GTGCATGATGTTCGAGTTATACCTGCAAACACACGAAGAGGAAAAGGAATTCTTCAAACCACTGATGGGAGCCTA
CCAGAAGAGCCGCTTAAACAGAGAAGCATTTACAAAAGACATCATGAAATACTCCACACCGATAACAGTAGGCAT
CGTCGATTGTGACACGTTTCTGAAAGCCGAAAAAGGAGTCATAAAACGCTTAGAGAAACTTGGGTTCAGTGGTTG
CGAATATGTCACAGATGAAGAGGCAATATTTCAAGCCCTAAATATGAAGGCGGCTGTTGGTGCACTATATAGTGG
AAAGAAGAGAGATTACTTTGAGAGCTACGGTCCAGAAGAAAAAGAGAATATCTTGAGAGAAAGCTGTAAGCGACT
ATACACAGGTAAGTTTGGAGTGTGGAATGGGTCACTCAAGTCGGAACTGAGACCTATGGAGAAAGTTATGGCAAA
TAAGACACGTGTCTTTACAGCAGCGCCGCTCGACACTCTGCTTGCTGGAAAAGTCTGTGTTGATGACTTCAACAA
TTACTTCTACAGCAAAAACATCGAGGCACCTTGGACAGTTGGCATGACGAAGTTTTATGGTGGGTGGAACGAACT
CCTTACGAAGTTACCAGACGGCTGGGTTTATTGCGATGCAGATGGATCACAATTCGATAGTTCTTTGTCACCATT
TCTCATCAACTCAGTTCTCAGAATACGTTTAAAGTTCATGGAAGATTGGGATCTTGGTGAACAAATGCTCAAAAA
TTTGTACACAGAAATTGTATATACAGCGATCCTCACTCCAGATTCAACCATAGTGAAGAAATTCAAGGGGAATAA
CAGCGGACAGCCATCCACGGTAGTAGATAACACATTGATGGTCGTGCTAGCAATGACATACACGCTGCACAAGTT
AGGCTTCGAGGACGAGGAACAAGACTCTATGTGCAAATACTTCGTCAATGGAGATGACCTGATCATCGCGATAAA
ACCAGAATATGAATCACTACTGGATCAGTTCCAACACTGTTTCAAAAGTTTGGGCTTAAATTACGATTTCAACTC
ACGAACGAGGAAGAGGGAGGAATTGTGGTTCATGTCACATTGCGGCATCAAGAAGGATGGAATCTTCATCCCAAA
ACTCGAACCAGAGCGCATTGTGTCAATCCTAGAGTGGGATAGATCAGACCAGCCAGTACATCGCTTAGAGGCCAT
ATGCGCAGCAATGATCGAATCATGGGGTTACGACAAGTTAACCCATGAAATTCGAAAATTTTACAAGTGGTGTCT
GGACGAAGCACCATATGCTGATTTGGCAAAAGCGGGAAAAGCACCGTACATAGCAGAGTGTGCTCTTAAACGATT
GTATACCAGCAAGGAAGCCAGCGAAGCAGAATTAGAAAAATACATGGAAGCCATACGTAGTCTTGTCAATGATGA
AGATGACGACGATATGGATGAAGTCTATCATCAA**GTGGACACAGTAATTTCTACTAAGTGTAGATGCAAGAAGAC**
CAGGCTCGAAAGCGACATGGACGAAGTTTATCACCAAGTAGACACGAAGCTTGATGCAGGCCAAGGCAGTAAAAA
TGATGATAAACAGAAGAGCTCAGCGGATTCAAAAGATAATGTCATCACGGAGAAAGGAAGTGGTTCTGGGCAGGT
GAGGAAGGATGACGACATCAACGCAGGGCTACATGGCAAACACACCATACCTCGTACAAAGGCAATCACACAGAA
AATGAAGTTACCAATGATCCGAGGTAAAGTGGCTTTGAACCTTGATCATTTGCTGGAGTACGAACCAAACCAGAG
AGACATATCAAACACACGCGCGACTCAAAAACAATACGAGTCATGGTACGACGGAGTTAAGAATGACTATGATGT
GGATGATAATGGCATGCAATTAATTCTGAACGGATTGATGGTTTGGTGTATAGAAAACGGGACATCCCCGAATAT
AAATGGAACATGGGTGATGATGGACAGTGAAGAACAAGTAGAATATGCTCTGAAACCCATCATCGAACACGCGAA
ACCCACGTTTCGCCAGATAATGGCCCATTTTAGTGACGCAGCCGAGGCGTACATTGAGATGAGAAACAAGAAGAA
ACCGTATATGCCACGATACGGACGGCTACGAGGCTTGAACGATATGGGGTTAGCTCGCTACGCTTTCGACTTTTA
CGAAACAACATCAGCGACCCCAAATCGGGCGAGAGAGGCGCACAATCAAATGAAGGCAGCTGCTCTAGTGGGAAC
ACAGAACAGACTGTTTGGAATGGATGGAGGCGGTTCAACCCAGGAAGAGAACACGGAGAGGCACACAGCCGCAGA
TGTAAATCAGAATATGCACACTCTCTTAGGCGTGAGAGGGTTGCACTAAAGGCGTGTGTTGGCATTTAAAGACTG
TAGTATAAATCTATAATATAGTGAGTGTTTCACTCCTTTTATGTTTATGTATGCACTTATTTGTCCGTTAGTATT
CTCTCCCTGTACTTCGCTCGTAAGGGCGTAGGTTCAGCTGAGGGCTTTTCTAGTTTCCGCTGTGAGGTTTTACCT
CGAAGGTTGCTAGTCTGGTTTTCAGTCGGAGACAAAAAAAAAAAAAAAAAAAAAAAAAAAAAAAAAAAAAAAAAAA
AAAAAAAA

LMV (recombinant between LMV-E [X97705.1] and LMV-AF199 [AJ278854.1]) with a cDNA corresponding to crRNA-3 inserted between the NIb and CP cistrons. crRNA-3 cDNA is flanked by sequences (green) to complement the split NIb/CP **NIaPro-mediated cleavage site**. A **G** extra nucleotide (blue background) was added to maintain the open reading frame. In

the crRNA-3 cDNA, **LbCas12a direct repeat** and **protospacer** are highlighted on yellow and green backgrounds, respectively.

Figure S5. Nucleotide sequences of the plasmids used for *Nicotiana benthamiana* (pGBKan-LbCas12att), and cultivated tabacum (*Nicotiana tabacum* cv. Ottawa) and tomato (*Solanum lycopersicum* cv. Moneymaker) (pLBHig-LbCas12att) stable transformation.

>pGBKan-LbCas12att
TGGCAGGATATATTGTGGTGTAAACATAACGAATTCGTCTCAGGAGAGCGATCAGCTTGCATGCCGGTCGATCTA
GTAACATAGATGACACCGCGCGCGATAATTTATCCTAGTTTGCGCGCTATATTTTGTTTTCTATCGCGTATTAAA
TGTATAATTGCGGGACTCTAATCATAAAAACCCATCTCATAAATAACGTCATGCATTACATGTTAATTATTACAT
GCTTAACGTAATTCAACAGAAATTATATGATAATCATGGCAAGACCGGCAACAGGATTCAATCTTAAGAAACTTT
ATTGCCAAATGTTTGAACGATCTGCTTGACTCTAGCTAGAGTCCGAACCCCAGAGTCCCGCTCAGAAGAACTCGT
CAAGAAGGCGATAGAAGGCTATGCGCTGCGAATCGGGAGCGGCGATACCGTAAAGCACGAGGAAGCGGTCAGCCC
ATTCGCCGCCAAGCTCTTCAGCAATATCACGGGTAGCCAACGCTATGTCCTGATAGCGGTCCGCCACACCCAGCC
GGCCACAGTCGATGAATCCAGAAAAGCGGCCATTTTCCACCATGATATTCGGCAAGCAGGCGTCGCCGTGGGTCA
CGACGAGATCCTCGCCGTCGGGCATCCGCGCCTTGAGCCTGGCGAACAGTTCGGCTGGCGCGAGCCCCTGATGCT
CTTCGTCCAGATCATCCTGATCGACAAGACCGGCTTCCATCCGAGTACGTGCTCGCTCGATGCGGTGTTTCGCTT
GGTGGTCGAATGGGCAGGTAGCCGGATCAAGCGTATGCAGCCGCCGCATTGCATCAGCCATGATGGATACTTTCT
CGGCAGGAGCAAGGTGAGATGACAGGAGATCCTGCCCCGGCACTTCGCCCAATAGCAGCCAGTCCCTTCCCGCTT
CAGTGACAACGTCGAGCACAGCTGCGCAAGGAACGCCCGTCGTGGCCAGCCACGATAGCCGCGCTGCCTCGTCTT
GGAGTTCATTCAGGGCACCGGACAGGTCGGTCTTGACAAAAAGAACCGGGCGCCCCTGCGCTGACAGCCGGAACA
CGGCGGCATCAGAGCAGCCGATTGTCTGTTGTGCCCAGTCATAGCCGAATAGCCTCTCCACCCAAGCGGCCGGAG
AACCTGCGTGCAATCCATCTTGTTCAATCATGCCTCGATCGAGTTGAGAGTGAATATGAGACTCTAATTGGATAC
CGAGGGGAATTTATGGAACGTCAGTGGAGCATTTTTGACAAGAAATATTTGCTAGCTGATAGTGACCTTAGGCGA
CTTTTGAACGCGCAATAATGGTTTCTGACGTATGTGCTTAGCTCATTAAACTCCAGAAACCCGCGGCTGAGTGGC
TCCTTCAACGTTGCGGTTCTGTCAGTTCCAAACGTAAAACGGCTTGTCCCGCGTCATCGGCGGGGGTCATAACGT
GACTCCCTTAATTCTCATGTATCTCCGTCAGGAGACTAGAGCCAAGCTGATCTCCTTTGCCCCGGAGATCACCAT
GGACGACTTTCTCTATCTCTACGATCTAGGAAGAAAGTTCGACGGAGAAGGTGACGATACCATGTTCACCACCGA
TAATGAGAAGATTAGCCTCTTCAATTTCAGAAAGAATGCTGACCCACAGATGGTTAGAGAGGCCTACGCGGCAGG
TCTGATCAAGACGATCTACCCGAGTAATAATCTCCAGGAGATCAAATACCTTCCCAAGAAGGTTAAAGATGCAGT
CAAAAGATTCAGGACTAACTGCATCAAGAACACAGAGAAAGATATATTTCTCAAGATCAGAAGTACTATTCCAGT
ATGGACGATTCAAGGCTTGCTTCATAAACCAAGGCAAGTAATAGAGATTGGAGTCTCTAAGAAAGTAGTTCCTAC
TGAATCAAAGGCCATGGAGTCAAAAATTCAGATCGAGGATCTAACAGAACTCGCCGTGAAGACTGGCGAACAGTT
CATACAGAGTCTTTTACGACTCAATGACAAGAAGAAAATCTTCGTCAACATGGTGGAGCACGACACTCTCGTCTA
CTCCAAGAATATCAAAGATACAGTCTCAGAAGACCAAAGGGCTATTGAGACTTTTCAACAAAGGGTAATATCGGG
AAACCTCCTCGGATTCCATTGCCCAGCTATCTGTCACTTCATCAAAAGGACAGTAGAAAAGGAAGGTGGCACCTA
CAAATGCCATCATTGCGATAAAGGAAAGGCTATCGTTCAAGATGCCTCTGCCGACAGTGGTCCCAAAGATGGACC
CCCACCCACGAGGAGCATCGTGGAAAAAGAAGACGTTCCAACCACGTCTTCAAAGCAAGTGGATTGATGTGATAT
CTCCACTGACGTAAGGGATGACGCACAATCCCACTATCCTTCGCAAGACCCTTCCTCTATATAAGGAAGTTCATT
TCATTTGGAGAGGACTCCGGTATTTTTACAACAATACCACAACAAAACAAACAACAAACAACATTACAATTTACT
ATTCTAGTCGAAATGAGCAAGCTCGAGAAGTTTACCAACTGCTACAGCCTCTCTAAGACCCTCAGGTTCAAGGCT
ATCCCTGTGGGAAAGACCCAAGAGAATATCGACAACAAGAGGCTCCTCGTCGAGGATGAGAAGAGAGCTGAAGAT
TACAAGGGCGTGAAGAAGCTCCTCGACAGGTACTACCTCAGCTTCATCAACGATGTGCTCCACAGCATCAAGCTC
AAGAACCTCAACAACTACATCAGCCTCTTCCGTAAGAAAACCAGGACCGAGAAAGAGAACAAAGAGCTTGAGAAC
CTCGAGATCAACCTCCGTAAAGAGATCGCCAAGGCTTTCAAGGGAAACGAGGGATACAAGAGCCTCTTCAAGAAG
GATATTATCGAGACAATCCTGCCTGAGTTCCTGGACGATAAGGATGAGATCGCTCTCGTGAACAGCTTCAACGGA
TTCACTACTGCCTTCACCGGATTCTTCAGAAACAGGGAAAACATGTTCAGCGAAGAGGCCAAGAGCACCTCTATC
GCTTTCAGATGCATCAACGAGAACCTCACGCGTTACATCAGCAACATGGACATCTTCGAGAAGGTGGACGCCATC
TTCGATAAGCACGAGGTGCAAGAAATCAAAGAGAAGATCCTCAACAGCGACTACGACGTCGAGGACTTTTTTGAA
GGGGAGTTCTTCAACTTCGTTCTCACCCAAGAGGGCATCGACGTGTACAACGCTATTATCGGAGGATTCGTGACC
GAGTCTGGGGAGAAGATTAAGGGACTCAACGAGTACATCAACCTGTACAACCAGAAAACGAAGCAGAAGCTCCCG
AAGTTCAAGCCGCTCTACAAGCAGGTTCTCTCTGATCGTGAGAGCCTCTCATTTTACGGTGAGGGTTACACCTCT
GACGAGGAAGTGCTTGAGGTTTTCCGTAACACCCTCAACAAGAACAGCGAGATCTTCTCGTCCATCAAGAAGTTG
GAGAAGCTTTTCAAGAACTTCGACGAGTACAGCAGCGCTGGGATCTTCGTTAAGAACGGACCTGCTATCAGCACC
ATCAGCAAGGATATTTTCGGCGAGTGGAACGTGATCAGGGACAAGTGGAATGCTGAGTACGATGACATCCACCTC
AAGAAGAAGGCTGTCGTCACTGAGAAGTACGAGGATGACAGGCGTAAGTCGTTCAAGAAGATCGGCTCTTTCAGC
CTCGAGCAGCTTCAAGAATACGCTGATGCTGATCTCAGCGTGGTCGAGAAGCTCAAAGAGATCATCATCCAGAAG
GTCGACGAGATCTACAAGGTGTACGGGTCCTCTGAGAAGTTGTTCGATGCTGATTTCGTCCTCGAGAAGAGTCTG
AAGAAGAACGACGCTGTCGTCGCGATCATGAAGGATTTGCTCGACAGCGTGAAGTCCTTCGAGAACTATATCAAG
GCCTTCTTCGGAGAGGGCAAAGAGACTAATAGGGACGAGTCTTTCTACGGGGATTTCGTGCTCGCTTACGATATC
CTCCTCAAGGTGGACCATATCTACGACGCCATCAGAAACTACGTGACCCAGAAGCCTTACAGCAAGGACAAGTTC
AAGTTGTACTTTCAGAACCCGCAGTTCATGGGCGGATGGGACAAAGACAAAGAGACAGATTACAGGGCCACCATC

CTCAGGTACGGGTCTAAGTACTACCTGGCCATCATGGACAAGAAATACGCCAAGTGCCTCCAAAAGATCGACAAG
GATGACGTGAACGGGAACTATGAGAAGATCAACTACAAGCTCCTTCCGGGACCGAACAAGATGCTTCCTAAGGTG
TTCTTCAGCAAGAAATGGATGGCCTACTACAACCCGTCTGAGGACATCCAGAAAATCTACAAGAACGGGACCTTC
AAGAAAGGCGACATGTTCAACCTCAACGACTGCCACAAGCTCATCGATTTCTTCAAGGACAGCATCTCGCGTTAC
CCGAAGTGGTCTAACGCTTACGACTTTAACTTCAGCGAGACAGAAAAGTACAAGGATATCGCCGGGTTCTACCGT
GAGGTTGAGGAACAGGGTTACAAGGTTAGCTTCGAGAGCGCCTCCAAGAAAGAGGTTGACAAGTTGGTCGAAGAG
GGCAAGCTCTACATGTTCCAGATCTATAACAAGGACTTCTCCGACAAGAGCCACGGAACTCCTAACCTCCATACG
ATGTACTTCAAGCTGCTTTTCGACGAGAACAACCACGGGCAGATCAGACTTTCTGGTGGTGCTGAACTCTTCATG
CGTAGGGCCTCACTCAAGAAAGAAGAGTTGGTTGTTCACCCGGCCAACTCTCCAATCGCTAACAAGAATCCTGAC
AACCCGAAAAAGACCACCACGCTGTCTTACGACGTCTACAAGGACAAAAGGTTCAGCGAGGACCAGTACGAGCTT
CATATCCCGATCGCTATCAACAAGTGCCCGAAGAACATCTTCAAGATCAATACCGAGGTGAGGGTGCTGCTCAAG
CACGATGATAACCCTTACGTGATCGGAATCGATCGTGGTGAGAGAAACCTCCTCTACATCGTTGTGGTGGACGGA
AAGGGAAACATCGTCGAGCAGTACAGCCTGAACGAGATTATCAACAATTTCAACGGCATCAGGATCAAGACCGAC
TACCACTCACTCCTCGATAAGAAAGAAAAAGAGCGTTTCGAGGCCAGGCAGAACTGGACTTCTATCGAAAACATC
AAAGAGTTGAAGGCCGGCTACATCTCTCAGGTGGTGCATAAGATCTGCGAGCTGGTGGAAAAGTACGATGCTGTG
ATCGCTCTTGAGGACCTCAACTCTGGGTTCAAGAACAGTAGAGTGAAGGTTGAGAAGCAGGTCTACCAAAAGTTC
GAGAAGATGCTCATCGACAAGCTCAACTACATGGTGGACAAAAAGAGCAACCCTTGCGCTACCGGTGGTGCTCTT
AAGGGATACCAGATCACGAACAAGTTCGAGTCCTTCAAGAGCATGAGCACCCAGAACGGCTTCATCTTCTATATC
CCTGCTTGGCTCACCAGCAAGATCGATCCTTCTACTGGTTTCGTGAACCTGCTCAAGACCAAGTACACCTCGATC
GCCGACAGCAAGAAGTTCATCTCGTCTTTCGACAGGATCATGTACGTGCCGGAAGAGGATCTTTTCGAGTTCGCT
CTCGACTATAAGAACTTCAGCAGGACCGACGCCGACTACATTAAGAAGTGGAAGCTCTACTCCTACGGGAACCGT
ATCAGGATCTTCCGAAATCCGAAGAAAAACAACGTGTTCGACTGGGAAGAAGTGTGCCTCACCTCTGCCTACAAA
GAACTGTTCAACAAGTACGGCATCAACTACCAGCAGGGTGATATCAGGGCTCTTTTGTGCGAGCAGAGCGACAAG
GCATTCTACAGCTCATTCATGGCCCTCATGTCTCTCATGCTCCAGATGAGGAACTCTATCACCGGAAGGACCGAT
GTGGACTTCCTTATCTCTCCGGTCAAGAACTCTGACGGGATCTTCTACGACAGCCGTAACTATGAGGCTCAAGAG
AACGCTATCCTGCCGAAGAATGCTGATGCAAACGGGGCTTACAACATTGCGAGAAAGGTTCTCTGGGCTATCGGG
CAGTTTAAGAAAGCGGAAGATGAGAAGCTGGACAAGGTGAAGATCGCCATCTCCAACAAAGAGTGGCTTGAGTAC
GCTCAGACCTCCGTTAAGCACAAGAGGCCTGCTGCTACTAAGAAAGCTGGTCAGGCTAAGAAGAAGAAATGAGCT
TCGGCCATGCTAGAGTCCGCAAAAATCACCAGTCTCTCTCTACAAATCTATCTCTCTCTATTTTTCTCCAGAATA
ATGTGTGAGTAGTTCCCAGATAAGGGAATTAGGGTTCTTATAGGGTTTCGCTCATGTGTTGAGCATATAAGAAAC
CCTTAGTATGTATTTGTATTTGTAAAATACTTCTATCAATAAAATTTCTAATTCCTAAAACCAAAATCCAGTGAC
CTCGCTGTCAGGAGACTAGAGCCAAGCTGATCTCCTTTGCCCCGGAGATCACCATGGACGACTTTCTCTATCTCT
ACGATCTAGGAAGAAAGTTCGACGGAGAAGGTGACGATACCATGTTCACCACCGATAATGAGAAGATTAGCCTCT
TCAATTTCAGAAAGAATGCTGACCCACAGATGGTTAGAGAGGCCTACGCGGCAGGTCTGATCAAGACGATCTACC
CGAGTAATAATCTCCAGGAGATCAAATACCTTCCCAAGAAGGTTAAAGATGCAGTCAAAAGATTCAGGACTAACT
GCATCAAGAACACAGAGAAAGATATATTTCTCAAGATCAGAAGTACTATTCCAGTATGGACGATTCAAGGCTTGC
TTCATAAACCAAGGCAAGTAATAGAGATTGGAGTCTCTAAGAAAGTAGTTCCTACTGAATCAAAGGCCATGGAGT
CAAAAATTCAGATCGAGGATCTAACAGAACTCGCCGTGAAGACTGGCGAACAGTTCATACAGAGTCTTTTACGAC
TCAATGACAAGAAGAAAATCTTCGTCAACATGGTGGAGCACGACACTCTCGTCTACTCCAAGAATATCAAAGATA
CAGTCTCAGAAGACCAAAGGGCTATTGAGACTTTTCAACAAAGGGTAATATCGGGAAACCTCCTCGGATTCCATT
GCCCAGCTATCTGTCACTTCATCAAAAGGACAGTAGAAAAGGAAGGTGGCACCTACAAATGCCATCATTGCGATA
AAGGAAAGGCTATCGTTCAAGATGCCTCTGCCGACAGTGGTCCCAAAGATGGACCCCCACCCACGAGGAGCATCG
TGGAAAAAGAAGACGTTCCAACCACGTCTTCAAAGCAAGTGGATTGATGTGATATCTCCACTGACGTAAGGGATG
ACGCACAATCCCACTATCCTTCGCAAGACCCTTCCTCTATATAAGGAAGTTCATTTCATTTGGAGAGGACTCCGG
TATTTTTACAACAATACCACAACAAAACAAACAACAAACAACATTACAATTTACTATTCTAGTCGAAATGGCCTC
CTCCGAGAACGTCATCACCGAGTTCATGCGCTTCAAGGTGCGCATGGAGGGCACCGTGAACGGCCACGAGTTCGA
GATCGAGGGCGAGGGCGAGGGCCGCCCCTACGAGGGCCACAACACCGTGAAGCTGAAGGTGACCAAGGGCGGCCC
CCTGCCCTTCGCCTGGGACATCCTGTCCCCCCAGTTCCAGTACGGCTCCAAGGTGTACGTGAAGCACCCCGCCGA
CATCCCCGACTACAAGAAGCTGTCCTTCCCCGAGGGCTTCAAGTGGGAGCGCGTGATGAACTTCGAGGACGGCGG
CGTGGCGACCGTGACCCAGGACTCCTCCCTGCAGGACGGCTGCTTCATCTACAAGGTGAAGTTCATCGGCGTGAA
CTTCCCCTCCGACGGCCCCGTGATGCAGAAGAAGACGATGGGCTGGGAGGCCTCCACCGAGCGCCTGTACCCCCG
CGACGGCGTGCTGAAGGGCGAGACACACAAGGCCCTGAAGCTGAAGGACGGCGGCCACTACCTGGTGGAGTTCAA
GTCCATCTACATGGCCAAGAAGCCCGTGCAGCTGCCCGGCTACTACTACGTGGACGCCAAGCTGGACATCACCTC
CCACAACGAGGACTACACCATCGTGGAGCAGTACGAGCGCACCGAGGGCCGCCACCACCTGTTCCTGTAGGCTTC
GGCCATGCTAGAGTCCGCAAAAATCACCAGTCTCTCTCTACAAATCTATCTCTCTCTATTTTTCTCCAGAATAAT
GTGTGAGTAGTTCCCAGATAAGGGAATTAGGGTTCTTATAGGGTTTCGCTCATGTGTTGAGCATATAAGAAACCC
TTAGTATGTATTTGTATTTGTAAAATACTTCTATCAATAAAATTTCTAATTCCTAAAACCAAAATCCAGTGACCT
CGCTGTCAGGAGTCTCAATGGTAACTTTACTCTTTATTTAACCATACATTTTTTTTTATTTTTTTCACTTTGTTC
TTCATCCACTATTGTTCTTTGTTCATCTTGAACAAAAGCTCCCTCCTTCTTTGTTCTTCATCCACCATTGTTCTT
CATCAATCATTTCGCTGTCATGAGACGAATTCTGACAGGATATATTGGCGGGTAAACCTAAGAGAAAAGAGCGTT
TATTAGAATAATCGGATATTTAAAAGGGCGTGAAAAGGTTTATCCGTTCGTCCATTTGTATGTGCATGCCAACCA

CAGGGTTCCCCTCGGGATCAAAGTACTTTGATCCAACCCCTCCGCTGCTATAGTGCAGTCGGCTTCTGACGTTCA
GTGCAGCCGTCATCTGAAAACGACATGTCGCACAAGTCCTAAGTTACGCGACAGGCTGCCGCCCTGCCCTTTTCC
TGGCGTTTTCTTGTCGCGTGTTTTAGTCGCATAAAGTAGAATACTTGCGACTAGAACCGGAGACATTACGCCATG
AACAAGAGCGCCGCCGCTGGCCTGCTGGGCTATGCCCGCGTCAGCACCGACGACCAGGACTTGACCAACCAACGG
GCCGAACTGCACGCGGCCGGCTGCACCAAGCTGTTTTCCGAGAAGATCACCGGCACCAGGCGCGACCGCCCGGAG
CTGGCCAGGATGCTTGACCACCTACGCCCTGGCGACGTTGTGACAGTGACCAGGCTAGACCGCCTGGCCCGCAGC
ACCCGCGACCTACTGGACATTGCCGAGCGCATCCAGGAGGCCGGCGCGGGCCTGCGTAGCCTGGCAGAGCCGTGG
GCCGACACCACCACGCCGGCCGGCCGCATGGTGTTGACCGTGTTCGCCGGCATTGCCGAGTTCGAGCGTTCCCTA
ATCATCGACCGCACCCGGAGCGGGCGCGAGGCCGCCAAGGCCCGAGGCGTGAAGTTTGGCCCCCGCCCTACCCTC
ACCCCGGCACAGATCGCGCACGCCCGCGAGCTGATCGACCAGGAAGGCCGCACCGTGAAAGAGGCGGCTGCACTG
CTTGGCGTGCATCGCTCGACCCTGTACCGCGCACTTGAGCGCAGCGAGGAAGTGACGCCCACCGAGGCCAGGCGG
CGCGGTGCCTTCCGTGAGGACGCATTGACCGAGGCCGACGCCCTGGCGGCCGCCGAGAATGAACGCCAAGAGGAA
CAAGCATGAAACCGCACCAGGACGGCCAGGACGAACCGTTTTTCATTACCGAAGAGATCGAGGCGGAGATGATCG
CGGCCGGGTACGTGTTCGAGCCGCCCGCGCACCTCTCAACCGTGCGGCTGCATGAAATCCTGGCCGGTTTGTCTG
ATGCCAAGCTGGCGGCCTGGCCGGCCAGCTTGGCCGCTGAAGAAACCGAGCGCCGCCGTCTAAAAAGGTGATGTG
TATTTGAGTAAAACAGCTTGCGTCATGCGGTCGCTGCGTATATGATCCGATGAGTAAATAAACAAATACGCAAGG
GGAACGCATGAAGGTTATCGCTGTACTTAACCAGAAAGGCGGGTCAGGCAAGACGACCATCGGAACCCATCTAGC
CCGCGCCCTGCAACTCGCCGGGGCCGATGTTCTGTTAGTCGATTCCGATCCCCAGGGCAGTGCCCGCGATTGGGC
GGCCGTGCGGGAAGATCAACCGCTAACCGTTGTCGGCATCGACCGCCCGACGATTGACCGCGACGTGAAGGCCAT
CGGCCGGCGCGACTTCGTAGTGATCGACGGAGCGCCCCAGGCGGCGGACTTGGCTGTGTCCGCGATCAAGGCAGC
CGACTTCGTGCTGATTCCGGTGCAGCCAAGCCCTTACGACATATGGGCCACCGCCGACCTGGTGGAGCTGGTTAA
GCAGCGCATTGAGGTCACGGATGGAAGGCTACAAGCGGCCTTTGTCGTGTCGCGGGCGATCAAAGGCACGCGCAT
CGGCGGTGAGGTTGCCGAGGCGCTGGCCGGGTACGAGCTGCCCATTCTTGAGTCCCGTATCACGCAGCGCGTGAG
CTACCCAGGCACTGCCGCCGCCGGCACAACCGTTCTTGAATCAGAACCCGAGGGCGACGCTGCCCGCGAGGTCCA
GGCGCTGGCCGCTGAAATTAAATCAAAACTCATTTGAGTTAATGAGGTAAAGAGAAAATGAGCAAAAGCACAAAC
ACGCTAAGTGCCGGCCGTCCGAGCGCACGCAGCAGCAAGGCTGCAACGTTGGCCAGCCTGGCAGACACGCCAGCC
ATGAAGCGGGTCAACTTTCAGTTGCCGGCGGAGGATCACACCAAGCTGAAGATGTACGCGGTACGCCAAGGCAAG
ACCATTACCGAGCTGCTATCTGAATAGATCGCGCAGCTACCAGAGTAAATGAGCAAATGAATAAATGAGTAGATG
AATTTTAGCGGCTAAAGGAGGCGGCATGGAAAATCAAGAACAACCAGGCACCGACGCCGTGGAATGCCCCATGTG
TGGAGGAACGGGCGGTTGGCCAGGCGTAAGCGGCTGGGTTGTCTGCCGGCCCTGCAATGGCACTGGAACCCCCAA
GCCCGAGGAATCGGCGTGACGGTCGCAAACCATCCGGCCCGGTACAAATCGGCGCGGCGCTGGGTGATGACCTGG
TGGAGAAGTTGAAGGCCGCGCAGGCCGCCCAGCGGCAACGCATCGAGGCAGAAGCACGCCCCGGTGAATCGTGGC
AAGCGGCCGCTGATCGAATCCGCAAAGAATCCCGGCAACCGCCGGCAGCCGGTGCGCCGTCGATTAGGAAGCCGC
CCAAGGGCGACGAGCAACCAGATTTTTTCGTTCCGATGCTCTATGACGTGGGCACCCGCGATAGTCGCAGCATCA
TGGACGTGGCCGTTTTCCGTCTGTCGAAGCGTGACCGACGAGCTGGCGAGGTGATCCGCTACGAGCTTCCAGACG
GGCACGTAGAGGTTTCCGCAGGGCCGGCCGGCATGGCCAGTGTGTGGGATTACGACCTGGTACTGATGGCGGTTT
CCCATCTAACCGAATCCATGAACCGATACCGGGAAGGGAAGGGAGACAAGCCCGGCCGCGTGTTCCGTCCACACG
TTGCGGACGTACTCAAGTTCTGCCGGCGAGCCGATGGCGGAAAGCAGAAAGACGACCTGGTAGAAACCTGCATTC
GGTTAAACACCACGCACGTTGCCATGCAGCGTACGAAGAAGGCCAAGAACGGCCGCCTGGTGACGGTATCCGAGG
GTGAAGCCTTGATTAGCCGCTACAAGATCGTAAAGAGCGAAACCGGGCGGCCGGAGTACATCGAGATCGAGCTAG
CTGATTGGATGTACCGCGAGATCACAGAAGGCAAGAACCCGGACGTGCTGACGGTTCACCCCGATTACTTTTTGA
TCGATCCCGGCATCGGCCGTTTTCTCTACCGCCTGGCACGCCGCGCCGCAGGCAAGGCAGAAGCCAGATGGTTGT
TCAAGACGATCTACGAACGCAGTGGCAGCGCCGGAGAGTTCAAGAAGTTCTGTTTCACCGTGCGCAAGCTGATCG
GGTCAAATGACCTGCCGGAGTACGATTTGAAGGAGGAGGCGGGGCAGGCTGGCCCGATCCTAGTCATGCGCTACC
GCAACCTGATCGAGGGCGAAGCATCCGCCGGTTCCTAATGTACGGAGCAGATGCTAGGGCAAATTGCCCTAGCAG
GGGAAAAAGGTCGAAAAGGACTCTTTCCTGTGGATAGCACGTACATTGGGAACCCAAAGCCGTACATTGGGAACC
GGAACCCGTACATTGGGAACCCAAAGCCGTACATTGGGAACCGGTCACACATGTAAGTGACTGATATAAAAGAGA
AAAAAGGCGATTTTTCCGCCTAAAACTCTTTAAAACTTATTAAAACTCTTAAAACCCGCCTGGCCTGTGCATAAC
TGTCTGGCCAGCGCACAGCCGAAGAGCTGCAAAAAGCGCCTACCCTTCGGTCGCTGCGCTCCCTACGCCCCGCCG
CTTCGCGTCGGCCTATCGCGGCCGCTGGCCGCTCAAAAATGGCTGGCCTACGGCCAGGCAATCTACCAGGGCGCG
GACAAGCCGCGCCGTCGCCACTCGACCGCCGGCGCCCACATCAAGGCACCCTGCCTCGCGCGTTTCGGTGATGAC
GGTGAAAACCTCTGACACATGCAGCTCCCGGTGACGGTCACAGCTTGTCTGTAAGCGGATGCCGGGAGCAGACAA
GCCCGTCAGGGCGCGTCAGCGGGTGTTGGCGGGTGTCGGGGCGCAGCCATGACCCAGTCACGTAGCGATAGCGGA
GTGTATACTGGCTTAACTATGCGGCATCAGAGCAGATTGTACTGAGAGTGCACCATATGCGGTGTGAAATACCGC
ACAGATGCGTAAGGAGAAAATACCGCATCAGGCGCTCTTCCGCTTCCTCGCTCACTGACTCGCTGCGCTCGGTCG
TTCGGCTGCGGCGAGCGGTATCAGCTCACTCAAAGGCGGTAATACGGTTATCCACAGAATCAGGGGATAACGCAG
GAAAGAACATGTGAGCAAAAGGCCAGCAAAAGGCCAGGAACCGTAAAAAGGCCGCGTTGCTGGCGTTTTTCCATA
GGCTCCGCCCCCCTGACGAGCATCACAAAAATCGACGCTCAAGTCAGAGGTGGCGAAACCCGACAGGACTATAAA
GATACCAGGCGTTTCCCCCTGGAAGCTCCCTCGTGCGCTCTCCTGTTCCGACCCTGCCGCTTACCGGATACCTGT
CCGCCTTTCTCCCTTCGGGAAGCGTGGCGCTTTCTCATAGCTCACGCTGTAGGTATCTCAGTTCGGTGTAGGTCG
TTCGCTCCAAGCTGGGCTGTGTGCACGAACCCCCCGTTCAGCCCGACCGCTGCGCCTTATCCGGTAACTATCGTC

TTGAGTCCAACCCGGTAAGACACGACTTATCGCCACTGGCAGCAGCCACTGGTAACAGGATTAGCAGAGCGAGGT
ATGTAGGCGGTGCTACAGAGTTCTTGAAGTGGTGGCCTAACTACGGCTACACTAGAAGGACAGTATTTGGTATCT
GCGCTCTGCTGAAGCCAGTTACCTTCGGAAAAAGAGTTGGTAGCTCTTGATCCGGCAAACAAACCACCGCTGGTA
GCGGTGGTTTTTTTGTTTGCAAGCAGCAGATTACGCGCAGAAAAAAAGGATCTCAAGAAGATCCTTTGATCTTTT
CTACGGGGTCTGACGCTCAGTGGAACGAAAACTCACGTTAAGGGATTTTGGTCATGCATTCTAGGTGATTAGAAA
AACTCATCGAGCATCAAATGAAACTGCAATTTATTCATATCAGGATTATCAATACCATATTTTTGAAAAAGCCGT
TTCTGTAATGAAGGAGAAAACTCACCGAGGCAGTTCCATAGGATGGCAAGATCCTGGTATCGGTCTGCGATTCCG
ACTCGTCCAACATCAATACAACCTATTAATTTCCCCTCGTCAAAAATAAGGTTATCAAGTGAGAAATCACCATGA
GTGACGACTGAATCCGGTGAGAATGGCAAAAGTTTATGCATTTCTTTCCAGACTTGTTCAACAGGCCAGCCATTA
CGCTCGTCATCAAAATCACTCGCATCAACCAAACCGTTATTCATTCGTGATTGCGCCTGAGCGAGTCGAAATACG
CGATCGCTGTTAAAAGGACAATTACAAACAGGAATCGAATGCAACCGGCGCAGGAACACTGCCAGCGCATCAACA
ATATTTTCACCTGAATCAGGATATTCTTCTAATACCTGGAATGCTGTTTTCCCTGGGATCGCAGTGGTGAGTAAC
CATGCATCATCAGGAGTACGGATAAAATGCTTGATGGTCGGAAGAGGCATAAATTCCGTCAGCCAGTTTAGTCTG
ACCATCTCATCTGTAACATCATTGGCAACGCTACCTTTGCCATGTTTCAGAAACAACTCTGGCGCATCGGGCTTC
CCATACAATCGGTAGATTGTCGCACCTGATTGCCCGACATTATCGCGAGCCCATTTATACCCATATAAATCAGCA
TCCATGTTGGAATTTAATCGCGGCCTTGAGCAAGACGTTTCCCGTTGAATATGGCTCATAACAGAACTTATTATT
TCCTTCCTCTTTTCTACAGTATTTAAAGATACCCCAAGAAGCTAATTATAACAAGACGAACTCCAATTCACTGTT
CCTTGCATTCTAAAACCTTAAATACCAGAAAACAGCTTTTTCAAAGTTGTTTTCAAAGTTGGCGTATAACATAGT
ATCGACGGAGCCGATTTTGAAACCGCGGTGATCACAGGCAGCAACGCTCTGTCATCGTTACAATCAACATGCTAC
CCTCCGCGAGATCATCCGTGTTTCAAACCCGGCAGCTTAGTTGCCGTTCTTCCGAATAGCATCGGTAACATGAGC
AAAGTCTGCCGCCTTACAACGGCTCTCCCGCTGACGCCGTCCCGGACTGATGGGCTGCCTGTATCGAGTGGTGAT
TTTGTGCCGAGCTGCCGGTCGGGGAGCTGTTGGCTGGCTGG

Plasmid from the GoldenBraid system. Between the T-DNA left and right borders (**LB** and **RB**, respectively), three different transcriptional units were inserted. First consisting in *A. tumefaciens Nopaline synthase* (*NoS*) **promoter** and **terminator** to express the **kanamycin resistance marker** (antisense orientation). Second to express **LbCas12att** including a nuclear localization signal NLS under the control of **CaMV 35S promoter** and **terminator**. Third to express fluorescent **DsRed** marker also under control of **CaMV 35S promoter** and **terminator**. **Start** and **stop** codons of the three proteins are underlined.

>pLBHig-LbCas12att
ACTAGTGCGGCG**GCGGCCGCGATTCCATTGCCCAGCTATCTGTCACTTTATTGTGAAGATAGTGGAAAAGGAAGG**
TGGCTCCTACAAATGCCATCATTGCGATAAAGGAAAGGCCATCGTTGAAGATGCCTCTGCCGACAGTGGTCCCAA
AGATGGACCCCCACCCACGAGGAGCATCGTGGAAAAAGAAGACGTTCCAACCACGTCTTCAAAGCAAGTGGATTG
ATGTGATAGATTCCATTGCCCAGCTATCTGTCACTTTATTGTGAAGATAGTGGAAAAGGAAGGTGGCTCCTACAA
ATGCCATCATTGCGATAAAGGAAAGGCCATCGTTGAAGATGCCTCTGCCGACAGTGGTCCCAAAGATGGACCCCC
ACCCACGAGGAGCATCGTGGAAAAAGAAGACGTTCCAACCACGTCTTCAAAGCAAGTGGATTGATGTGATATCTC
CACTGACGTAAGGGATGACGCACAATCCCACTATCCTTCGCAAGACCCTTCCTCTATATAAGGAAGTTCATTTCA
TTTGGAGAGGTATTAAAATCTTAATAGGTTTTGATAAAAGCGAACGTGGGGAAACCCGAACCAAACCTTCTTCTA
AACTCTCTCTCATCTCTCTTAAAGCAAACTTCTCTCTTGTCTTTCTTGCGTGAGCGATCTTCAACGTTGTCAGAT
CGTGCTTCGGCACCAGTACAACGTTTTCTTTCACTGAAGCGAAATCAAAGATCTCTTTGTGGACACGTAGTGCGG
CGCCATTAAATAACGTGTACTTGTCCTATTCTTGTCGGTGTGGTCTTGGGAAAAGAAAGCTTGCTGGAGGCTGCT
GTTCAGCCCCATACATTACTTGTTACGATTCTGCTGACTTTCGGCGGGTGCAATATCTCTACTTCTGCTTGACGA
GGTATTGTTGCCTGTACTTCTTTCTTCTTCTTCTTGCTGATTGGTTCTATAAGAAATCTAGTATTTTCTTTGAAA
CAGAGTTTTCCCGTGGTTTTCGAACTTGGAGAAAGATTGTTAAGCTTCTGTATATTCTGCCCAAATTTGAAATGA
GCAAGCTGGAGAAGTTTACAAACTGCTACTCCCTGTCTAAGACCCTGAGGTTCAAGGCCATCCCTGTGGGCAAGA
CCCAGGAGAACATCGACAATAAGCGGCTGCTGGTGGAGGACGAGAAGAGAGCCGAGGATTATAAGGGCGTGAAGA
AGCTGCTGGATCGCTACTATCTGTCTTTTATCAACGACGTGCTGCACAGCATCAAGCTGAAGAATCTGAACAATT
ACATCAGCCTGTTCCGGAAGAAAACCAGAACCGAGAAGGAGAATAAGGAGCTGGAGAACCTGGAGATCAATCTGC
GGAAGGAGATCGCCAAGGCCTTCAAGGGCAACGAGGGCTACAAGTCCCTGTTTAAGAAGGATATCATCGAGACAA
TCCTGCCAGAGTTCCTGGACGATAAGGACGAGATCGCCCTGGTGAACAGCTTCAATGGCTTTACCACAGCCTTCA
CCGGCTTCTTTCGTAACAGAGAGAATATGTTTTCCGAGGAGGCCAAGAGCACATCCATCGCCTTCAGGTGTATCA
ACGAGAATCTGACCCGCTACATCTCTAATATGGACATCTTCGAGAAGGTGGACGCCATCTTTGATAAGCACGAGG
TGCAGGAGATCAAGGAGAAGATCCTGAACAGCGACTATGATGTGGAGGATTTCTTTGAGGGCGAGTTCTTTAACT
TTGTGCTGACACAGGAGGGCATCGACGTGTATAACGCCATCATCGGCGGCTTCGTGACCGAGAGCGGCGAGAAGA
TCAAGGGCCTGAACGAGTACATCAACCTGTATAATCAGAAAACCAAGCAGAAGCTGCCTAAGTTTAAGCCACTGT
ATAAGCAGGTGCTGAGCGATCGGGAGTCTCTGAGCTTCTACGGCGAGGGCTATACATCCGATGAGGAGGTGCTGG
AGGTGTTTAGAAACACCCTGAACAAGAACAGCGAGATCTTCAGCTCCATCAAGAAGCTGGAGAAGCTGTTCAAGA
ATTTTGACGAGTACTCTAGCGCCGGCATCTTTGTGAAGAACGGCCCCGCCATCAGCACAATCTCCAAGGATATCT

**TCGGCGAGTGGAACGTGATCCGGGACAAGTGGAATGCCGAGTATGACGATATCCACCTGAAGAAGAAGGCCGTGG
TGACCGAGAAGTACGAGGACGATCGGAGAAAGTCCTTCAAGAAGATCGGCTCCTTTTCTCTGGAGCAGCTGCAGG
AGTACGCCGACGCCGATCTGTCTGTGGTGGAGAAGCTGAAGGAGATCATCATCCAGAAGGTGGATGAGATCTACA
AGGTGTATGGCTCCTCTGAGAAGCTGTTCGACGCCGATTTTGTGCTGGAGAAGAGCCTGAAGAAGAACGACGCCG
TGGTGGCCATCATGAAGGACCTGCTGGATTCTGTGAAGAGCTTCGAGAATTACATCAAGGCCTTCTTTGGCGAGG
GCAAGGAGACAAACAGGGACGAGTCCTTCTATGGCGATTTTGTGCTGGCCTACGACATCCTGCTGAAGGTGGACC
ACATCTACGATGCCATCCGCAATTATGTGACCCAGAAGCCCTACTCTAAGGATAAGTTCAAGCTGTATTTTCAGA
ACCCTCAGTTCATGGGCGGCTGGGACAAGGATAAGGAGACAGACTATCGGGCCACCATCCTGAGATACGGCTCCA
AGTACTATCTGGCCATCATGGATAAGAAGTACGCCAAGTGCCTGCAGAAGATCGACAAGGACGATGTGAACGGCA
ATTACGAGAAGATCAACTATAAGCTGCTGCCCGGCCCTAATAAGATGCTGCCAAAGGTGTTCTTTTCTAAGAAGT
GGATGGCCTACTATAACCCCAGCGAGGACATCCAGAAGATCTACAAGAATGGCACATTCAAGAAGGGCGATATGT
TTAACCTGAATGACTGTCACAAGCTGATCGACTTCTTTAAGGATAGCATCTCCCGGTATCCAAAGTGGTCCAATG
CCTACGATTTCAACTTTTCTGAGACAGAGAAGTATAAGGACATCGCCGGCTTTTACAGAGAGGTGGAGGAGCAGG
GCTATAAGGTGAGCTTCGAGTCTGCCAGCAAGAAGGAGGTGGATAAGCTGGTGGAGGAGGGCAAGCTGTATATGT
TCCAGATCTATAACAAGGACTTTTCCGATAAGTCTCACGGCACACCCAATCTGCACACCATGTACTTCAAGCTGC
TGTTTGACGAGAACAATCACGGACAGATCAGGCTGAGCGGAGGAGCAGAGCTGTTCATGAGGCGCGCCTCCCTGA
AGAAGGAGGAGCTGGTGGTGCACCCAGCCAACTCCCCTATCGCCAACAAGAATCCAGATAATCCCAAGAAAACCA
CAACCCTGTCCTACGACGTGTATAAGGATAAGAGGTTTTCTGAGGACCAGTACGAGCTGCACATCCCAATCGCCA
TCAATAAGTGCCCCAAGAACATCTTCAAGATCAATACAGAGGTGCGCGTGCTGCTGAAGCACGACGATAACCCCT
ATGTGATCGGCATCGATAGGGGCGAGCGCAATCTGCTGTATATCGTGGTGGTGGACGGCAAGGGCAACATCGTGG
AGCAGTATTCCCTGAACGAGATCATCAACAACTTCAACGGCATCAGGATCAAGACAGATTACCACTCTCTGCTGG
ACAAGAAGGAGAAGGAGAGGTTCGAGGCCCGCCAGAACTGGACCTCCATCGAGAATATCAAGGAGCTGAAGGCCG
GCTATATCTCTCAGGTGGTGCACAAGATCTGCGAGCTGGTGGAGAAGTACGATGCCGTGATCGCCCTGGAGGACC
TGAACTCTGGCTTTAAGAATAGCCGCGTGAAGGTGGAGAAGCAGGTGTATCAGAAGTTCGAGAAGATGCTGATCG
ATAAGCTGAACTACATGGTGGACAAGAAGTCTAATCCTTGTGCAACAGGCGGCGCCCTGAAGGGCTATCAGATCA
CCAATAAGTTCGAGAGCTTTAAGTCCATGTCTACCCAGAACGGCTTCATCTTTTACATCCCTGCCTGGCTGACAT
CCAAGATCGATCCATCTACCGGCTTTGTGAACCTGCTGAAAACCAAGTATACCAGCATCGCCGATTCCAAGAAGT
TCATCAGCTCCTTTGACAGGATCATGTACGTGCCCGAGGAGGATCTGTTCGAGTTTGCCCTGGACTATAAGAACT
TCTCTCGCACAGACGCCGATTACATCAAGAAGTGGAAGCTGTACTCCTACGGCAACCGGATCAGAATCTTCCGGA
ATCCTAAGAAGAACAACGTGTTCGACTGGGAGGAGGTGTGCCTGACCAGCGCCTATAAGGAGCTGTTCAACAAGT
ACGGCATCAATTATCAGCAGGGCGATATCAGAGCCCTGCTGTGCGAGCAGTCCGACAAGGCCTTCTACTCTAGCT
TTATGGCCCTGATGAGCCTGATGCTGCAGATGCGGAACAGCATCACAGGCCGCACCGACGTGGATTTTCTGATCA
GCCCTGTGAAGAACTCCGACGGCATCTTCTACGATAGCCGGAACTATGAGGCCCAGGAGAATGCCATCCTGCCAA
AGAACGCCGACGCCAATGGCGCCTATAACATCGCCAGAAAGGTGCTGTGGGCCATCGGCCAGTTCAAGAAGGCCG
AGGACGAGAAGCTGGATAAGGTGAAGATCGCCATCTCTAACAAGGAGTGGCTGGAGTACGCCCAGACCAGCGTGA
AGCACAAAAGGCCGGCGGCCACGAAAAAGGCCGGCCAGGCAAAAAAGAAAAAGGGATCCCCAAAGAAGAAGCGGA
AGGTCTAACTCTGGTTTCATTAAATTTTCTTTAGTTTGAATTTACTGTTATTCGGTGTGCATTTCTATGTTTGGT
GAGCGGTTTTCTGTGCTCAGAGTGTGTTTATTTTATGTAATTTAATTTCTTTGTGAGCTCCTGTTTAGCAGGTCG
TCCCTTCAGCAAGGACACAAAAAGATTTTAATTTTATTCGCTGAAATCACCAGTCTCTCTCTACAAATCTATCTC
TCTCTATTTTCTCCATAAATAATGTGTGAGTAGTTTCCCGATAAGGGAAATTAGGGTTCTTATAGGGTTTCGCTC
ATGTGTTGAGCATATAAGAAACCCTTAGTATGTATTTGTATTTGTAAAATACTTCTATCAATAAAATTTCTAATT
CCTAAAACCAAAATCCAGGGGCCCGATCATGAGCGGAGAATTAAGGGAGTCACGTTATGACCCCCGCCGATGACG
CGGGACAAGCCGTTTTACGTTTGGAACTGACAGAACCGCAACGTTGAAGGAGCCACTCAGCCGCGGGTTTCTGGA
GTTTAATGAGCTAAGCACATACGTCAGAAACCATTATTGCGCGTTCAAAAGTCGCCTAAGGTCACTATCAGCTAG
CAAATATTTCTTGTCAAAAATGCTCCACTGACGTTCCATAAATTCCCCTCGGTATCCAATTAGAGTCTCATATTC
ACTCTCAATCCAAATAATCTGCAATGAAAAAGCCTGAACTCACCGCGACGTCTGTCGAGAAGTTTCTGATCGAAA
AGTTCGACAGCGTCTCCGACCTGATGCAGCTCTCGGAGGGCGAAGAATCTCGTGCTTTCAGCTTCGATGTAGGAG
GGCGTGGATATGTCCTGCGGGTAAATAGCTGCGCCGATGGTTTCTACAAAGATCGTTATGTTTATCGGCACTTTG
CATCGGCCGCGCTCCCGATTCCGGAAGTGCTTGACATTGGGGAGTTTAGCGAGAGCCTGACCTATTGCATCTCCC
GCCGTTCACAGGGTGTCACGTTGCAAGACCTGCCTGAAACCGAACTGCCCGCTGTTCTACAACCGGTCGCGGAGG
CTATGGATGCGATCGCTGCGGCCGATCTTAGCCAGACGAGCGGGTTCGGCCCATTCGGACCGCAAGGAATCGGTC
AATACACTACATGGCGTGATTTCATATGCGCGATTGCTGATCCCCATGTGTATCACTGGCAAACTGTGATGGACG
ACACCGTCAGTGCGTCCGTCGCGCAGGCTCTCGATGAGCTGATGCTTTGGGCCGAGGACTGCCCCGAAGTCCGGC
ACCTCGTGCACGCGGATTTCGGCTCCAACAATGTCCTGACGGACAATGGCCGCATAACAGCGGTCATTGACTGGA
GCGAGGCGATGTTCGGGGATTCCCAATACGAGGTCGCCAACATCTTCTTCTGGAGGCCGTGGTTGGCTTGTATGG
AGCAGCAGACGCGCTACTTCGAGCGGAGGCATCCGGAGCTTGCAGGATCGCCACGACTCCGGGCGTATATGCTCC
GCATTGGTCTTGACCAACTCTATCAGAGCTTGGTTGACGGCAATTTCGATGATGCAGCTTGGGCGCAGGGTCGAT
GCGACGCAATCGTCCGATCCGGAGCCGGGACTGTCGGGCGTACACAAATCGCCCGCAGAAGCGCGGCCGTCTGGA
CCGATGGCTGTGTAGAAGTACTCGCCGATAGTGGAAACCGACGCCCCAGCACTCGTCCGAGGGCAAAGAAATAAA
GAAGGAGTGCGTCGAAGCAGATCGTTCAAACATTTGGCAATAAAGTTTCTTAAGATTGAATCCTGTTGCCGGTCT
TGCGATGATTATCATATAATTTCTGTTGAATTACGTTAAGCATGTAATAATTAACATGTAATGCATGACGTTATT**

TATGAGATGGGTTTTTATGATTAGAGTCCCGCAATTATACATTTAATACGCGATAGAAAACAAAATATAGCGCGC
AAACTAGGATAAATTATCGCGCGCGGTGTCATCTATGTTACTAGATCGACGCGTTGAGACGGTTTCGACCAGGCT
ACTAGAGTACTTAATTAATCTTTTTCTCCA**TTGTTTACACCACAATATATCCTGCCACCAG**TAGATTTCCCGGAC
ATGAAGCC**ATTTACAATTGAATATATCCTGCCG**CCGCTGCCGCTTTGCACCCGGTGGAGCTTGCATGTTGGTTTC
TACGCAGAACTGAGCCGGTTTGTTATCAATAAAAAGGCCGCGATTTGCGGCCTTATTGTTCGTCGGAAGACCA
CCGAACTGATGATGGCCCCCTACGGGCTTGCTCTCCGGGCTTCGCCCTGCGCGGTCGCTGCGCTCCCTTGCCAGC
CCGTGGATATGTGGACGATGGCCGCGAGCGGCCACCGGCTGGCTCGCTTCGCTCGGCCCGTGGACAACCCTGCTG
GACAAGCTGATGGACAGGCTGCGCCTGCCCACGAGCTTGACCACAGGGATTGCCCACCGGCTACCCAGCCTTCGA
CCACATACCCACCGGCTCCAACTGCGCGGCCTGCGGCCTTGCCCCATCAATTTTTTTAATTTTCTCTGGGGAAAA
GCCTCCGGCCTGCGGCCTGCGCGCTTCGCTTGCCGGTTGGACACCAAGTGGAAGGCGGGTCAAGGCTCGCGCAGC
GACCGCGCAGCGGCTTGGCCTTGACGCGCCTGGAACGACCCAAGCCTATGCGAGTGGGGGCAGTCGAAGGCGAA
GCCCGCCCGCCTGCCCCCCGAGCCTCACGGCGGCGAGTGCGGGGGTTCCAAGGGGGCAGCGCCACCTTGGGCAAG
GCCGAAGGCCGCGCAGTCGATCAACAAGCCCCGGAGGGGCCACTTTTTGCCGGAGGGGGAGCCGCGCCGAAGGCG
TGGGGGAACCCCGCAGGGGTGCCCTTCTTTGGGCACCAAAGAACTAGATATAGGGCGAAATGCGAAAGACTTAAA
AATCAACAACTTAAAAAAGGGGGGTACGCAACAGCTCATTGCGGCACCCCCCGCAATAGCTCATTGCGTAGGTTA
AAGAAAATCTGTAATTGACTGCCACTTTTACGCAACGCATAATTGTTGTCGCGCTGCCGAAAAGTTGCAGCTGAT
TGCGCATGGTGCCGCAACCGTGCGGCACCCCTACCGCATGGAGATAAGCATGGCCACGCAGTCCAGAGAAATCGG
CATTCAAGCCAAGAACAAGCCCGGTCACTGGGTGCAAACGGAACGCAAAGCGCATGAGGCGTGGGCCGGGCTTAT
TGCGAGGAAACCCACGGCGGCAATGCTGCTGCATCACCTCGTGGCGCAGATGGGCCACCAGAACGCCGTGGTGGT
CAGCCAGAAGACACTTTCCAAGCTCATCGGACGTTCTTTGCGGACGGTCCAATACGCAGTCAAGGACTTGGTGGC
CGAGCGCTGGATCTCCGTCGTGAAGCTCAACGGCCCCGGCACCGTGTCGGCCTACGTGGTCAATGACCGCGTGGC
GTGGGGCCAGCCCCGCGACCAGTTGCGCCTGTCGGTGTTCAGTGCCGCCGTGGTGGTTGATCACGACGACCAGGA
CGAATCGCTGTTGGGGCATGGCGACCTGCGCCGCATCCCGACCCTGTATCCGGGCGAGCAGCAACTACCGACCGG
CCCCGGCGAGGAGCCGCCCAGCCAGCCCGGCATTCCGGGCATGGAACCAGACCTGCCAGCCTTGACCGAAACGGA
GGAATGGGAACGGCGCGGGCAGCAGCGCCTGCCGATGCCCGATGAGCCGTGTTTTCTGGACGATGGCGAGCCGTT
GGAGCCGCCGACACGGGTAACGCTGCCGCGCCGGTAGAACCGCATAACCGCCAATCCGATCTTGTGTCTCAAAAT
CTCTGATGTTACATTGCACAAGATAAAAATATATCATCATGAACAATAAAACTGTCTGCTTACATAAACAGTAAT
ACAAGGGGTGTT**ATGAGCCATATTCAGCGTGAAACGAGCTGTAGCCGTCCGCGTCTGAACAGCAACATGGATGCG**
GATCTGTATGGCTATAAATGGGCGCGTGATAACGTGGGTCAGAGCGGCGCGACCATTTATCGTCTGTATGGCAAA
CCGGATGCGCCGGAACTGTTTCTGAAACATGGCAAAGGCAGCGTGGCGAACGATGTGACCGATGAAATGGTGCGT
CTGAACTGGCTGACCGAATTTATGCCGCTGCCGACCATTAAACATTTTATTCGCACCCCGGATGATGCGTGGCTG
CTGACCACCGCGATTCCGGGCAAAACCGCGTTTCAGGTGCTGGAAGAATATCCGGATAGCGGCGAAAACATTGTG
GATGCGCTGGCCGTGTTTCTGCGTCGTCTGCATAGCATTCCGGTGTGCAACTGCCCGTTTAACAGCGATCGTGTG
TTTCGTCTGGCCCAGGCGCAGAGCCGTATGAACAACGGCCTGGTGGATGCGAGCGATTTTGATGATGAACGTAAC
GGCTGGCCGGTGGAACAGGTGTGGAAAGAAATGCATAAACTGCTGCCGTTTAGCCCGGATAGCGTGGTGACCCAC
GGCGATTTTAGCCTGGATAACCTGATTTTCGATGAAGGCAAACTGATTGGCTGCATTGATGTGGGCCGTGTGGGC
ATTGCGGATCGTTATCAGGATCTGGCCATTCTGTGGAACTGCCTGGGCGAATTTAGCCCGAGCCTGCAAAAACGT
CTGTTTCAGAAATATGGCATTGATAATCCGGATATGAACAAACTGCAATTTCATCTGATGCTGGATGAATTTTTC
TAATAGTTGATTGCAAAGGACCGCATGTCGCAAGTCGGCGCGCCACACTAACTAAAAACACCCTAACGGGTGTTT
TTTCTTTTCTTGTTCTTCCAAAAAAATTTACAACCGAACAAACAAACAAATACAGAAATTTAGACAATAAAAATG
A**ATTACAACCGCATATATCCTGCCA**TCAGTCATCACAAAATCAACAATTCCTGCAGGCTAGCTCGCATCCTTGTC
CCGTCTCCTGAC

Plasmid derived from pLX-B2 (Pasin et al. 2017). **LbCas12a** coding sequence in black followed by double NLS (**nucleoplasmin**, **GS** spacers and **SV40**, in blue and purple, respectively). D156R (CG) thermotolerant mutation on fuchsia background. **Double CaMV 35S promoter** in garnet and red. Transcription **+1** nucleotide underlined. **CaMV 35S terminator** in fuchsia with the processing and polyadenylation site underlined. **5' and 3' UTR** derived from cowpea mosaic virus (CPMV) RNA2 in green. **Hygromycin** resistance cassette in green under the control of the *A. tumefaciens NoS* **promoter** and **terminator**. Unique 5' **SpeI**, **ApaI**, **NotI**, and 3' **MluI** sites in the T-DNA of pLX-B2 are indicated in blue on yellow background. Double **LB** and **RB** of the T-DNA are indicated on yellow and red backgrounds, respectively. **Kanamycin** resistance for bacterial selection is denoted in light grey.

Figure S6. Full nucleotide sequences of intermediate and final plasmids to express TEV-crRNA-3, TuMV-crRNA-3 and LMV-crRNA-3 vectors.

```
>pGTEV-G2 (12 812 bp)
GCGGCCGCGATTCCATTGCCCAGCTATCTGTCACTTTATTGTGAAGATAGTGGAAAAGGAAGGTGGCTCCTACAA
ATGCCATCATTGCGATAAAGGAAAGGCCATCGTTGAAGATGCCTCTGCCGACAGTGGTCCCAAAGATGGACCCCC
ACCCACGAGGAGCATCGTGGAAAAAGAAGACGTTCCAACCACGTCTTCAAAGCAAGTGGATTGATGTGATATCTC
CACTGACGTAAGGGATGACGCACAATCCCACTATCCTTCGCAAGACCCTTCCTCTATATAAGGAAGTTCATTTCA
TTTGGAGAGGAAAATAACAAATCTCAACACAACATATACAAAACAAACGAATCTCAAGCAATCAAGCATTCTACT
TCTATTGCAGCAATTTAAATCATTTCTTTTAAAGCAAAAGCAATTTTCTGAAAATTTTCACCATTTACGAACGAT
AGCCATGGCACTCATCTTTGGCACAGTCAACGCTAACATCCTGAAGGAAGTGTTCGGTGGAGCTCGTATGGCTTG
CGTTACCAGCGCACATATGGCTGGAGCGAATGGAAGCATTTTGAAGAAGGCAGAAGAAACCTCTCGTGCAATCAT
GCACAAACCAGTGATCTTCGGAGAAGACTACATTACCGAGGCAGACTTGCCTTACACACCACTCCATTTAGAGGT
CGATGCTGAAATGGAGCGGATGTATTATCTTGGTCGTCGCGCGCTCACCCATGGCAAGAGACGCAAAGTTTCTGT
GAATAACAAGAGGAACAGGAGAAGGAAAGTGGCCAAAACGTACGTGGGGCGTGATTCCATTGTTGAGAAGATTGT
AGTGCCCCACACCGAGAGAAAGGTTGATACCACAGCAGCAGTGGAAGACATTTGCAATGAAGCTACCACTCAACT
TGTGCATAATAGTATGCCAAAGCGTAAGAAGCAGAAAAACTTCTTGCCCGCCACTTCACTAAGTAACGTGTATGC
CCAAACTTGGAGCATAGTGCGCAAACGCCATATGCAGGTGGAGATCATTAGCAAGAAGAGCGTCCGAGCGAGGGT
CAAGAGATTTGAGGGCTCGGTGCAATTGTTCGCAAGTGTGCGTCACATGTATGGCGAGAGGAAAAGGGTGGACTT
ACGTATTGACAACTGGCAGCAAGAGACACTTCTAGACCTTGCTAAAAGATTTAAGAATGAGAGAGTGGATCAATC
GAAGCTCACTTTTGGTTCAAGTGGCCTAGTTTTGAGGCAAGGCTCGTACGGACCTGCGCATTGGTATCGACATGG
TATGTTCATTGTACGCGGTCGGTCGGATGGGATGTTGGTGGATGCTCGTGCGAAGGTAACGTTCGCTGTTTGTCA
CTCAATGACACATTATAGCGACAAATCAATCTCTGAGGCATTCTTCATACCATACTCTAAGAAATTCTTGGAGTT
GAGGCCAGATGGAATCTCCCATGAGTGTACAAGAGGAGTATCAGTTGAGCGGTGCGGTGAGGTGGCTGCAATCCT
GACACAAGCACTTTCACCGTGTGGTAAGATCACATGCAAACGTTGCATGGTTGAAACACCTGACATTGTTGAGGG
TGAGTCGGGAGACAGTGTCACCAACCAAGGTAAGCTCCTAGCAATGCTGAAAGAACAGTATCCAGATTTCCCAAT
GGCCGAGAAACTACTCACAAGGTTTTTGCAACAGAAATCACTAGTAAATACAAATTTGACAGCCTGCGTGAGCGT
CAAACAACTCATTGGTGACCGCAAACAAGCTCCATTCACACACGTACTGGCTGTCAGCGAAATTCTGTTTAAAGG
CAATAAACTAACAGGGGCCGATCTCGAAGAGGCAAGCACACATATGCTTGAAATAGCAAGGTTCTTGAACAATCG
CACTGAAAATATGCGCATTGGCCACCTTGGTTCTTTCAGAAATAAAATCTCATCGAAGGCCCATGTGAATAACGC
ACTCATGTGTGATAATCAACTTGATCAGAATGGGAATTTTATTTGGGGACTAAGGGGTGCACACGCAAAGAGGTT
TCTTAAAGGATTTTTCACTGAGATTGACCCAAATGAAGGATACGATAAGTATGTTATCAGGAAACATATCAGGGG
TAGCAGAAAGCTAGCAATTGGCAATTTGATAATGTCAACTGACTTCCAGACGCTCAGGCAACAAATTCAAGGCGA
AACTATTGAGCGTAAAGAAATTGGGAATCACTGCATTTCAATGCGGAATGGTAATTACGTGTACCCATGTTGTTG
TGTTACTCTTGAAGATGGTAAGGCTCAATATTCGGATCTAAAGCATCCAACGAAGAGACATCTGGTCATTGGCAA
CTCTGGCGATTCAAAGTACCTAGACCTTCCAGTTCTCAATGAAGAGAAAATGTATATAGCTAATGAAGGTTATTG
CTACATGAACATTTTCTTTGCTCTACTAGTGAATGTCAAGGAAGAGGATGCAAAGGACTTCACCAAGTTTATAAG
GGACACAATTGTTCCAAAGCTTGGAGCGTGGCCAACAATGCAAGATGTTGCAACTGCATGCTACTTACTTTCCAT
TCTTTACCCAGATGTCCTGAGTGCTGAATTACCCAGAATTTTGGTTGATCATGACAACAAAACAATGCATGTTTT
GGATTCGTATGGGTCTAGAACGACAGGATACCACATGTTGAAAATGAACACAACATCCCAGCTAATTGAATTCGT
TCATTCAGGTTTGGAATCCGAAATGAAAACTTACAATGTTGGAGGGATGAACCGAGATATGGTCACACAAGGTGC
AATTGAGATGTTGATCAAGTCCATATACAAACCACATCTCATGAAGCAGTTACTTGAGGAGGAGCCATACATAAT
TGTCCTGGCAATAGTCTCCCCTTCAATTTTAATTGCCATGTACAACTCTGGAACTTTTGAGCAGGCGTTACAAAT
GTGGTTGCCAAATACAATGAGGTTAGCTAACCTCGCTGCCATCTTGTCAGCCTTGGCGCAAAAGTTAACTTTGGC
AGACTTGTTCGTCCAGCAGCGTAATTTGATTAATGAGTATGCGCAGGTAATTTTGGACAATCTGATTGACGGTGT
CAGGGTTAACCATTCGCTATCCCTAGCAATGGAAATTGTTACTATTAAGCTGGCCACCCAAGAGATGGACATGGC
GTTGAGGGAAGGTGGCTATGCTGTGACCTCTGAAAAGGTGCATGAAATGTTGGAAAAAAACTATGTAAAGGCTTT
GAAGGATGCATGGGACGAATTAACTTGGTTGGAAAAATTCTCCGCAATCAGGCATTCAAGAAAGCTCTTGAAATT
TGGGCGAAAGCCTTTAATCATGAAAAACACCGTAGATTGCGGCGGACATATAGACTTGTCTGTGAAATCGCTTTT
CAAGTTCCACTTGGAACTCCTGAAGGGAACCATCTCAAGAGCCGTAAATGGTGGTGCAAGAAAGGTAAGAGTAGC
GAAGAATGCCATGACAAAAGGGGTTTTTCTCAAAATCTACAGCATGCTTCCTGACGTCTACAAGTTTATCACAGT
CTCGAGTGTCCTTTCCTTGTTGTTGACATTCTTATTTCAAATTGACTGCATGATAAGGGCACACCGAGAGGCGAA
GGTTGCTGCACAGTTGCAGAAAGAGAGCGAGTGGGACAATATCATCAATAGAACTTTCCAGTATTCTAAGCTTGA
AAATCCTATTGGCTATCGCTCTACAGCGGAGGAAAGACTCCAATCAGAACACCCCGAGGCTTTCGAGTACTACAA
GTTTTGCATTGGAAAGGAAGACCTCGTTGAACAGGCAAAACAACCGGAGATAGCATACTTTGAAAAGATTATAGC
TTTCATCACACTTGTATTAATGGCTTTTGACGCTGAGCGGAGTGATGGAGTGTTCAAGATACTCAATAAGTTCAA
AGGAATACTGAGCTCAACGGAGAGGGAGATCATCTACACGCAGAGTTTGGATGATTACGTTACAACCTTTGATGA
CAATATGACAATCAACCTCGAGTTGAATATGGATGAACTCCACAAGACGAGCCTTCCTGGAGTCACTTTTAAGCA
ATGGTGGAACAACCAAATCAGCCGAGGCAACGTGAAGCCACATTATAGAACTGAGGGGCACTTCATGGAGTTTAC
CAGAGATACTGCGGCATCGGTTGCCAGCGAGATATCACACTCACCCGCAAGAGATTTTCTTGTGAGAGGTGCTGT
TGGATCTGGAAAATCCACAGGACTTCCATACCATTTATCAAAGAGAGGGAGAGTGTTAATGCTTGAGCCTACCAG
```

ACCACTCACAGATAACGTGCACAAGCAACTGAGAAGTGAACCATTTAACTGCTTCCCAACTTTGAGGATGAGAGG
GAAGTCAACTTTTGGGTCATCACCGATTACAGTCATGACTAGTGGATTCGCTTTACACCATTTTGCACGAAACAT
AGCTGAGGTAAAAACATACGATTTTGTCATAATTGATGAATGTCATGTGAATGATGCTTCTGCTATAGCGTTTAG
GAATCTACTGTTTGAACATGAATTTGAAGGAAAAGTCCTCAAAGTGTCAGCCACACCACCAGGTAGAGAAGTTGA
ATTCACAACTCAGTTTCCCGTGAAACTCAAGATAGAAGAGGCTCTTAGCTTTCAGGAATTTGTAAGTTTACAAGG
GACAGGTGCCAACGCCGATGTGATTAGTTGTGGCGACAACATACTAGTATATGTTGCTAGCTACAATGATGTTGA
TAGTCTTGGCAAGCTCCTTGTGCAAAAGGGATACAAAGTGTCGAAGATTGATGGAAGAACAATGAAGAGTGGAGG
AACTGAAATAATCACTGAAGGTACTTCAGTGAAAAAGCATTTCATAGTCGCAACTAATATTATTGAGAATGGTGT
AACCATTGACATTGATGTAGTTGTGGATTTTGGGACTAAGGTTGTACCAGTTTTGGATGTGGACAATAGAGCGGT
GCAGTACAACAAAACTGTGGTGAGTTATGGGGAGCGCATCCAAAGACTCGGTAGAGTTGGGCGACACAAGGAAGG
AGTAGCACTTCGAATTGGCCAAACAAATAAAACACTGGTTGAAATTCCAGAAATGGTTGCCACTGAAGCTGCCTT
TCTATGCTTCATGTACAATTTGCCAGTGACAACACAGAGTGTTTCAACCACACTGCTGGAAAATGCCACATTATT
ACAAGCTAGAACTATGGCACAGTTTGAGCTATCATATTTTTACACAATTAATTTTGTGCGATTTGATGGTAGTAT
GCATCCAGTCATACATGACAAGCTGAAGCGCTTTAAGCTACACACTTGTGAGACATTCCTCAATAAGTTGGCGAT
CCCAAATAAAGGCTTATCCTCTTGGCTTACGAGTGGAGAGTATAAGCGACTTGGTTACATAGCAGAGGATGCTGG
CATAAGAATCCCATTCGTGTGCAAAGAAATTCCAGACTCCTTGCATGAGGAAATTTGGCACATTGTAGTCGCCCA
TAAAGGTGACTCGGGTATTGGGAGGCTCACTAGCGTACAGGCAGCAAAGGTTGTTTATACTCTGCAAACGGATGT
GCACTCAATTGCGAGGACTCTAGCATGCATCAATAGACTCATAGCACATGAACAAATGAAGCAGAGTCATTTTGA
AGCCGCAACTGGGAGAGCATTTTCCTTCACAAATTACTCAATACAAAGCATATTTGACACGCTGAAAGCAAATTA
TGCTACAAAGCATACGAAAGAAAATATTGCAGTGCTTCAGCAGGCAAAAGATCAATTGCTAGAGTTTTCGAACCT
AGCAAAGGATCAAGATGTCACGGGTATCATCCAAGACTTCAATCACCTGGAAACTATCTATCTCCAATCAGATAG
CGAAGTGGCTAAGCATCTGAAGCTTAAAAGTCACTGGAATAAAAGCCAAATCACTAGGGACATCATAATAGCTTT
GTCTGTGTTAATTGGTGGTGGATGGATGCTTGCAACGTACTTCAAGGACAAGTTCAATGAACCAGTCTATTTCCA
AGGGAAGAAGAATCAGAAGCACAAGCTTAAGATGAGAGAGGCGCGTGGGGCTAGAGGGCAATATGAGGTTGCAGC
GGAGCCAGAGGCGCTAGAACATTACTTTGGAAGCGCATATAATAACAAAGGAAAGCGCAAGGGCACCACGAGAGG
AATGGGTGCAAAGTCTCGGAAATTCATAAACATGTATGGGTTTGATCCAACTGATTTTTCATACATTAGGTTTGT
GGATCCATTGACAGGTCACACTATTGATGAGTCCACAAACGCACCTATTGATTTAGTGCAGCATGAGTTTGGAAA
GGTTAGAACACGCATGTTAATTGACGATGAGATAGAGCCTCAAAGTCTTAGCACCCACACCACAATCCATGCTTA
TTTGGTGAATAGTGGCACGAAGAAAGTTCTTAAGGTTGATTTAACACCACACTCGTCGCTACGTGCGAGTGAGAA
ATCAACAGCAATAATGGGATTTCCTGAAAGGGAGAATGAATTGCGTCAAACCGGCATGGCAGTGCCAGTGGCTTA
TGATCAATTGCCACCAAAGAGTGAGGACTTGACGTTTGAAGGAGAAAGCTTGTTTAAGGGACCACGTGATTACAA
CCCGATATCGAGCACCATTTGTCACTTGACGAATGAATCTGATGGGCACACAACATCGTTGTATGGTATTGGATT
TGGTCCCTTCATCATTACAAACAAGCACTTGTTTAGAAGAAATAATGGAACACTGTTGGTCCAATCACTACATGG
TGTATTCAAGGTCAAGAACACCACGACTTTGCAACAACACCTCATTGATGGGAGGGACATGATAATTATTCGCAT
GCCTAAGGATTTCCCACCATTTCCTCAAAAGCTGAAATTTAGAGAGCCACAAAGGGAAGAGCGCATATGTCTTGT
GACAACCAACTTCCAAACTAAGAGCATGTCTAGCATGGTGTCAGACACTAGTTGCACATTCCCTTCATCTGATGG
CATATTCTGGAAGCATTGGATTCAAACCAAGGATGGGCAGTGTGGCAGTCCATTAGTATCAACTAGAGATGGGTT
CATTGTTGGTATACACTCAGCATCGAATTTCACCAACACAAACAATTATTTCACAAGCGTGCCGAAAAACTTCAT
GGAATTGTTGACAAATCAGGAGGCGCAGCAGTGGGTTAGTGGTTGGCGATTAAATGCTGACTCAGTATTGTGGGG
GGGCCATAAAGTTTTCATGAGCAAACCTGAAGAGCCTTTTCAGCCAGTTAAGGAAGCGACTCAACTCATGAGTGA
ATTGGTGTACTCGCAAGGGGAGAAGAGGAAATGGGTCGTGGAAGCACTGTCAGGGAACTTGAGGCCAGTGGCTGA
GTGTCCCAGTCAGTTAGTCACAAAGCATGTGGTTAAAGGAAAGTGTCCCCTCTTTGAGCTCTACTTGCAGTTGAA
TCCAGAAAAGGAAGCATATTTTAAACCGATGATGGGAGCATATAAGCCAAGTCGACTTAATAGAGAGGCGTTCCT
CAAGGACATTCTAAAATATGCTAGTGAAATTGAGATTGGGAATGTGGATTGTGACTTGCTGGAGCTTGCAATAAG
CATGCTCATCACAAAGCTCAAGGCGTTAGGATTCCCAACTGTGAACTACATCACTGACCCAGAGGAAATTTTTAG
TGCATTGAATATGAAAGCAGCTATGGGAGCACTATACAAAGGCAAGAAGAAAGAAGCTCTCAGCGAGCTCACACT
AGATGAGCAGGAGGCAATGCTCAAAGCAAGTTGCCTGCGACTGTATACGGGAAAGCTGGGAATTTGGAATGGCTC
ATTGAAAGCAGAGTTGCGTCCAATTGAGAAGGTTGAAAACAACAAAACGCGAACTTTCACAGCAGCACCAATAGA
CACTCTTCTTGCTGGTAAAGTTTGCGTGGATGATTTCAACAATCAATTTTATGATCTCAACATAAAGGCACCATG
GACAGTTGGTATGACTAAGTTTTATCAGGGGTGGAATGAATTGATGGAGGCTTTACCAAGTGGGTGGGTGTATTG
TGACGCTGATGGTTCGCAATTCGACAGTTCCTTGACTCCATTCCTCATTAATGCTGTATTGAAAGTGCGACTTGC
CTTCATGGAGGAATGGGATATTGGTGAGCAAATGCTGCGAAATTTGTACACTGAGATAGTGTATACACCAATCCT
CACACCGGATGGTACTATCATTAAGAAGCATAAAGGCAACAATAGCGGGCAACCTTCAACAGTGGTGGACAACAC
ACTCATGGTCATTATTGCAATGTTATACACATGTGAGAAGTGTGGAATCAACAAGGAAGAGATTGTGTATTACGT
CAATGGCGATGACCTATTGATTGCCATTCACCCAGATAAAGCTGAGAGGTTGAGTGGATTCAAAGAATCTTTCGG
AGAGTTGGGCCTGAAATATGAATTTGACTGCACCACCAGGGACAAGACACAGTTGTGGTTCATGTCACACAGGGC
TTTGGAGAGGGATGGCATGTATATACCAAAGCTAGAAGAAGAAAGGATTGTTTCTATTTTGGAATGGGACAGATC
CAAAGAGCCGTCACATAGGCTTGAAGCCATCTGTGCATCAATGATCGAAGCATGGGGTTATGACAAGCTGGTTGA
AGAAATCCGCAATTTCTATGCATGGGTTTTGGAACAAGCGCCGTATTCACAGCTTGCAGAAGAAGGAAAGGCGCC
ATATCTGGCTGAGACTGCGCTTAAGTTTTTGTACACATCTCAGCACGGAACAAACTCTGAGATAGAAGAGTATTT
AAAAGTGTTGTATGATTACGATATTCCAACGACTGAGAATCTTTATTTTCAGTCAGGTACACGCGTGCGGTTAAT

TAAGGCGTCCGGACTACAGAGAACCTCTACTTTCAAAGTGGCACTGTGGGTGCTGGTGTTGACGCTGGTAAGAAG
AAAGATCAAAAGGATGATAAAGTCGCTGAGCAGGCTTCAAAGGATAGGGATGTTAATGCTGGAACTTCAGGAACA
TTCTCAGTTCCACGAATAAATGCTATGGCCACAAAACTTCAATATCCAAGGATGAGGGGAGAGGTGGTTGTAAAC
TTGAATCACCTTTTAGGATACAAGCCACAGCAAATTGATTTGTCAAATGCTCGAGCCACACATGAGCAGTTTGCC
GCGTGGCATCAGGCAGTGATGACAGCCTATGGAGTGAATGAAGAGCAAATGAAAATATTGCTAAATGGATTTATG
GTGTGGTGCATAGAAAATGGGACTTCCCCAAATTTGAACGGAACTTGGGTTATGATGGATGGTGAGGAGCAAGTT
TCATACCCGCTGAAACCAATGGTTGAAAACGCGCAGCCAACACTGAGGCAAATTATGACACACTTCAGTGACCTG
GCTGAAGCGTATATTGAGATGAGGAATAGGGAGCGACCATACATGCCTAGGTATGGTCTACAGAGAAACATTACA
GACATGAGTTTGTCACGCTATGCGTTCGACTTCTATGAGCTAACTTCAAAAACACCTGTTAGAGCGAGGGAGGCG
CATATGCAAATGAAAGCTGCTGCAGTACGAAACAGTGGAACTAGGTTATTTGGTCTTGATGGCAACGTGGGTACT
GCAGAGGAAGACACTGAACGGCACACAGCGCACGATGTGAACCGTAACATGCACACACTATTAGGGGTCCGCCAG
TGATAGTTTCTGCGTGTCTTTGCTTTCCGCTTTTAAGCTTATTGTAATATATATGAATAGCTATTCACAGTGGGA
CTTGGTCTTGTGTTGAATGGTATCTTATATGTTTTAATATGTCTTATTAGTCTCATTACTTAGGCGAACGACAAA
GTGAGGTCACCTCGGTCTAATTCTCCTATGTAGTGCGAAAAAAAAAAAAAAAAAAAAAAAAAAAAAAAAAAAAAA
AAAAAAAAAGATCTCGCTGAAATCACCAGTCTCTCTCTACAAATCTATCTCTCTCTATTTTCTCCATAAATAATG
TGTGAGTAGTTTCCCGATAAGGGAAATTAGGGTTCTTATAGGGTTTCGCTCATGTGTTGAGCATATAAGAAACCC
TTAGTATGTATTTGTATTTGTAAAATACTTCTATCAATAAAATTTCTAATTCCTAAAACCAAAATCCAGGGGCCC
TCGACGTTCCTTGACAGGATATATTGGCGGGTAAACTAAGTCGCTGTATGTGTTTGTTTGAGATCCTCTAGGGCA
TGCAAGCTGATCTGGATCTCATGTGAGCAAAAGGCCAGCAAAAGGCCAGGAACCGTAAAAAGGCCGCGTTGCTGG
CGTTTTTCCATAGGCTCCGCCCCCCTGACGAGCATCACAAAAATCGACGCTCAAGTCAGAGGTGGCGAAACCCGA
CAGGACTATAAAGATACCAGGCGTTTCCCCCTGGAAGCTCCCTCGTGCGCTCTCCTGTTCCGACCCTGCCGCTTA
CCGGATACCTGTCCGCCTTTCTCCCTTCGGGAAGCGTGGCGCTTTCTCATAGCTCACGCTGTAGGTATCTCAGTT
CGGTGTAGGTCGTTCGCTCCAAGCTGGGCTGTGTGCACGAACCCCCCGTTCAGCCCGACCGCTGCGCCTTATCCG
GTAACTATCGTCTTGAGTCCAACCCGGTAAGACACGACTTATCGCCACTGGCAGCAGCCACTGGTAACAGGATTA
GCAGAGCGAGGTATGTAGGCGGTGCTACAGAGTTCTTGAAGTGGTGGCCTAACTACGGCTACACTAGAAGAACAG
TATTTGGTATCTGCGCTCTGCTGAAGCCAGTTACCTTCGGAAGAAGAGTTGGTAGCTCTTGATCCGGCAAACAAA
CCACCGCTGGTAGCGGTGGTTTTTTTGTTTGCAAGCAGCAGATTACGCGCAGAAAAAAAGGATCTCAAGAAGATC
CTTTGATCTTTTCTACGGGGTCTGACGCTCAGTGGAACGAAAACTCACGTTAAGGGATTTTGGTCATGAGATTAT
CAAAAAGGATCTTCACCTAGATCCTTTTAAATTAAAAATGAAGTTTTAAATCAATCTAAAGTATATATGTGTAAC
ATTGGTCTAGTGATTAGAAAAACTCATCGAGCATCAAATGAAACTGCAATTTATTCATATCAGGATTATCAATAC
CATATTTTTGAAAAAGCCGTTTCTGTAATGAAGGAGAAAACTCACCGAGGCAGTTCCATAGGATGGCAAGATCCT
GGTATCGGTCTGCGATTCCGACTCGTCCAACATCAATACAACCTATTAATTTCCCCTCGTCAAAAATAAGGTTAT
CAAGTGAGAAATCACCATGAGTGACGACTGAATCCGGTGAGAATGGCAAAAGTTTATGCATTTCTTTCCAGACTT
GTTCAACAGGCCAGCCATTACGCTCGTCATCAAAATCACTCGCATCAACCAAACCGTTATTCATTCGTGATTGCG
CCTGAGCGAGACGAAATACGCGATCGCTGTTAAAAGGACAATTACAAACAGGAATCGAATGCAACCGGCGCAGGA
ACACTGCCAGCGCATCAACAATATTTTCACCTGAATCAGGATATTCTTCTAATACCTGGAATGCTGTTTTCCCTG
GGATCGCAGTGGTGAGTAACCATGCATCATCAGGAGTACGGATAAAATGCTTGATGGTCGGAAGAGGCATAAATT
CCGTCAGCCAGTTTAGTCTGACCATCTCATCTGTAACAACATTGGCAACGCTACCTTTGCCATGTTTCAGAAACA
ACTCTGGCGCATCGGGCTTCCCATACAATCGGTAGATTGTCGCACCTGATTGCCCGACATTATCGCGAGCCCATT
TATACCCATATAAATCAGCATCCATGTTGGAATTTAATCGCGGCCTTGAGCAAGACGTTTCCCGTTGAATATGGC
TCATAACACCCCTTGTATTACTGTTTATGTAAGCAGACAGTTTTATTGTTCATGATGATATATTTTTATCTTGTG
CAATGTAACATCAGAGATTTTGAGACACAACGTGGCTTTGTTGAATAAATCGAACTTTTGCTGAGTTGAAGGATC
AGATCACGCATCTTCCCGACAACGCAGACCGTTCCGTGGCAAAGCAAAAGTTCAAAATCACCAACTGGTCCACCT
ACAACAAAGCTCTCATCAACCGTGGCTCCCTCACTTTCTGGCTGGATGATGGGGCGATTCAGGCGATCCCCATCC
AACAGCCCGCCGTCGAGCGGGCTTTTTTATCCCCGGAAGCCTGTGGATAGAGGGTAGTTATCCACGTGAAACCGC
TAATGCCCCGCAAAGCCTTGATTCACGGGGCTTTCCGGCCCGCTCCAAAAACTATCCACGTGAAATCGCTAATCA
GGGTACGTGAAATCGCTAATCGGAGTACGTGAAATCGCTAATAAGGTCACGTGAAATCGCTAATCAAAAAGGCAC
GTGAGAACGCTAATAGCCCTTTCAGATCAACAGCTTGCAAACACCCCTCGCTCCGGCAAGTAGTTACAGCAAGTA
GTATGTTCAATTAGCTTTTCAATTATGAATATATATATCAATTATTGGTCGCCCTTGGCTTGTGGACAATGCGCT
ACGCGCACCGGCTCCGCCCGTGGACAACCGCAAGCGGTTGCCCACCGTCGAGCGCCTTTGCCCACAACCCGGCGG
CCGGCCGCAACAGATCGTTTTATAAATTTTTTTTTTTGAAAAAGAAAAAGCCCGAAAGGCGGCAACCTCTCGGGC
TTCTGGATTTCCGATCCCCGGAATTAGATCCGTTTAAACTACGTAAGATCGATCTTGGCAGGATATATTGTGGTG
TAAACGTTCCTGCGGCGGTCGAGATGGATCTTGGCAGGATATATTGTGGTGTAAACGTTCCT

TEV-DQ986288 (G273A, A1119G) in black with a polylinker with sites **MluI**, **Kpn2I** (also **PacI**) to insert cDNAs of interest by Gibson assembly between NIb and CP cistrons. The heterologous protein will be flanked by an artificial **NIaPro** proteolytic site (+3/-8, purple with silent mutations with respect to the native NIb/CP site underlined). **Silent mutations** (G273A, A1119G) in red on yellow background. Cistron **borders** on blue background. **Plasmid backone** in blue. Sites **NotI**, **BglII** and **ApaI** on yellow background. CaMV 35S **promoter**

(transcription start underlined) and **terminator** (processing and polyadenylation site underlined) in red and fuchsia, respectively. pUC replication origin on gray background. Marcador de selección *Kanamycin* selection marker (complementary) on dark gray background. pSa replication origin on gray background.T-DNA **RB** with **overdrive** (underlined) on yellow background and **double LB** on read background.

>pGTEV-crRNA-3
Insert between positions 8836 and 8863 of pGTEV-G2
GTAATTTCTACTAAGTGTAGATGCAAGAAGACCAGGCTCGAAAGC

>pGTuMVJPN1-K
GCGGCCGCGATTCCATTGCCCAGCTATCTGTCACTTTATTGTGAAGATAGTGGAAAAGGAAGGTGGCTCCTACAA
ATGCCATCATTGCGATAAAGGAAAGGCCATCGTTGAAGATGCCTCTGCCGACAGTGGTCCCAAAGATGGACCCCC
ACCCACGAGGAGCATCGTGGAAAAAGAAGACGTTCCAACCACGTCTTCAAAGCAAGTGGATTGATGTGATATCTC
CACTGACGTAAGGGATGACGCACAATCCCACTATCCTTCGCAAGACCCTTCCTCTATATAAGGAAGTTCATTTCA
TTTGGAGAGGAAAAAATATAAAAACTCAACACAACATACACAAAACGATCAAAGCAAACACAAATCTCTCGAAGC
ATTCAAGCAACCAAAGATTTTCAAATCTTCCATCGTTTTCAAAGCAACCAACAACAACGCAAGCAATGGCAGCAG
TTACATTTGCAACCGCAATCACTAACACCACCGCAAGCAAACCAGCACTCACCGGAATGATACAGTTTGGGAATT
TCCCACCAGTGCCATTGCGATCCACCACCGTTACCACAGTCGCCACTTCAGTGGCGCAACCTAAACTGCACACAG
TGCAGTTTGGAAGCCTTGACCCAGTAGTCGTCAAGAGTGGAGCAGGGTCCTTTGCTAAGGCAACACGCCAGCAGC
CTAACGTTGAAATAGACGTTAGCCTCAGTGAAGCCGCAGCTCTGGAGGTTGCGAAACCTAGACCAAACGCCGTGT
TGAGGATGCACGAGGAAGCAAACAAGGAGAGGGCACTCTTTTTGGACTGGGAGGCTAGTTTGAAGAGAAGCTCAT
ATGGAATTGCTGAGAACGAGAAAGTTGTAATGACAACTCGTGGCGTCAGCAAGATAGTGCCCAGAAGTTCAAGGG
CAATGAAGCAAAAGCGCGCAAGGGAAAGACGTAGAGCGCAACAACCAATCATACTAAAGTGGGAGCCTAAATTGA
GCGGGATCTCAATTGGAGGAGGGCTCTCCGCGAGCGCGATCGAAGTGGAAGAAGCCCGCACAAAGTGGCCGCTTC
ACAAGACACCGTCAATGAAGAGGAAGACGGTGCACAGAAGATGCAAGATGAATGATCAAGGAATTGACATGTTAA
TGCGTTCTTTGATTAAAATCTTTAAGGCGAAGAGTGCGAATATTGAGTTCATTGGAAGGAAGTCCATTAAAGTGG
ATTTCGTAAAGAAAGAGCAAACGAAGTTTGCCAGAGTACAAGTAGTGCATTTGCTCGGAAAGCGAGCACAACGCG
ACCTGTTGACTGGTGCAGAAGAGAACCACTTCATTGACACCCTAAGCAACTACTCAGGAAATAGGAAAATCATAA
ACCCAGGAGTGGTGTGCGCTGGTTGGAGTGGCATTGTTATCAGAAACGGCATTTTGACTCAGAAGCAGAGCAGAA
GTCCTTCGCAAGCCTTTGTGATTCGAGGGGAACATGAAGGGAAGTTGTATGACGCCAGAGTTAAGGTCACAAAGG
TGATGAGCCACAAAATCATACACTATAGTGCGGCAGGAGCCAATTTTTGGAAAGGATTTGACGGGTGTTTCCTCG
CATATCGAGGTGACAACCGTGAACATACATGTTATACGGGATTGGATGTCACCGAGTGTGGGGAGGTTGCAGCGC
TAATGTGTTTAGCCATGTTCCCATGTGGAAAAATAACTTGTCCTGACTGCGTAACAGACAGCGAGTTGTCTCAAG
GGCAGGCAAGTGGACCATCCATAAAACACAAGTTAGCACAACTACGTGAGGTTATCAAGTCCAGTTATCCACGGT
TTAAACATGCAGTGCAAATACTCGACAGATATGAGCAGTCATTGAGCAGTGCAAATGAGAACTATCAGGATTTTG
CGGAAATTCAAAGCATAAGTGATGGAACAGACAAAGCAGCGTTTCCGCACATTAATAAACTAAATGCAATATTAA
TTAAGGGAGCGACAGCAACTGGAGAGGAATTCTCGCAAGCAACAAAGTACTTGCTTGAGATAGCCCGGTATCTGA
AGAATAGAACCGAGAATATTGAAAAGGGTTCGCTTAAATCCTTCCGCAATAAGATCTCTCAAAAGGCGCACATAA
ACCCAACATTGATGTGTGACAATCAGCTCGACAGGAACGGCAACTTCATATGGGGTGAGAGAGGTTATCACGCGA
AGCGGTTCTTTAGTAACTACTTCGAAATAATCGATCCAAAGAAAGGCTACACTCAATACGAGACGAGAATAGTGC
CAAACGGATCACGAAAGCTCGCAATTGGCAAACTCATAGTCCCAACGAACTTCGAAGTCCTGAGGGAGCAGATGA
AAGGCGAGCCCATAGAACCACATCCAATCACAGTTGAGTGCGTAAGCAAGCTACAGGGCGACTTCGTTCATGCGT
GCTGTTGTGTTACAACGGAGTCAGGCGACCCAGTCTTGTCTGAAATTAAGATGCCAACTAAGCACCACCTGGTAA
TTGGTAATAGCGGCGACCCAAAGTACATCGATCTCCCTGAAATTGAGGAAAACAAGATGTACATAGCGAAAGAAG
GCTATTGCTACATTAATATCTTCTTAGCTATGCTAGTTAATGTTAAAGAGTCGCAAGCAAAAGAATTTACAAAAG
TGGTCAGAGACAAACTGGTTGGAGAACTTGGCAAATGGCCCACTTTGTTGGATGTAGCGACTGCTTGTTACTTCC
TAAAAGTGTTTTACCCTGACGTCGCCAACGCTGAATTGCCACGCATGCTAGTGGACCACAAAACGAAGATTATCC
ACGTAGTCGACTCGTATGGGTCACTGTCAACAGGATATCACATCCTTAAGACGAACACCGTGGAACAACTTATCA
AATTCACTAGGTGCAATTTAGAATCGAGTTTGAAGCACTACCGTGTTGGAGGCACCGCGTGGGAAGGAGCCCATG
GGTTTAATAACATAGACGACCCGCAATGGTGCATTAAAAGGCTCATTCAAGGAGTGTACAGGCCAAAGAAGTTGA
GGGAAGACATGCTGACAAACCCTTTTCTAACACTGTACGCCTTGTTATCACCAGGAGTGATTCTAGCATTCTATA
ACAGTGGCTCACTGGAGTACCTCATGAATCACTACATTAGAGCAGATAGCAACGTAGCTGTTCTGTTAGTAGTGC
TAAAATCTCTAGCAAAGAAAGTGTCAACCAGCCAGAGTGTGTTAGCTCAACTCCAAATAATCGAGCGAAGTCTGC
CGGAACTCGTGGAAGCCAGGGCTAATATAACTGGACCAGATGAAGCAGCCTCCCAGGCATGCAATAGATTCCTGG
GCATGCTCATCCATATGGCAGAACCAAATAACGAACTAGCAGATGGCGGATATACAATCCTGAGGGATCATAGTA
TCTCCATCTTAGAAAAAAGTTATCTGCAAATCTTGGACGAAGCATGGAGCGAATTAAGCTGGTCGGAGCGTTGTG
CTATAAAATACTACTCGTCAAAGCAAGCAATCTTTTCACAGAAAGATTTGCAAATGCGAAGCGACGTCGATTTAG
GCGGCAGATACAGCGAGTCAGTCACGTCCTCCTACGAGTGGGGTAAACAACGCGTGAAAAGCGTGTATTCTAATG
CATGTAATAAAGTACGTAGTAGTGTATCTTGGACTAGTAGTAAAATTTCAAGTAGTGTTTGTAAAACAATCAACT

ATTTAGTACCAGATGTGTTCAAATTCATTAATGTATTAGTTTGCATTAGTTTATTAGTCACAATAGCTGCTGAAG
CAAATCGTATTGTCACCACACAGAGGAGACTCAAACTGGACATCGAGGAAACAGAGCGCAAGAAGATTGAATGGG
AACTCGCATTCCATCATGCTATTCTAACGCAAAGTGCAGGGCAGCACCCAACACTAGATGAGTTCACAGCATACA
TTGGTGAGAAAGCACCTCATCTTAATGAGCATATTGAACCTGAGGAGAAGGCAGTAGTGCACCAAGCGAAAAGAC
AATCTGAGCAGGAGCTCGAACGAGTGATAGCTTTTATCGCCTTAGTTCTCATGATGTTTGACGCAGAACGCAGTG
ATTGCGTCACGAAGATCCTCAACAAGCTTAAGGGGTTAGTGTCAACTGTGGAACCTACAGTTTACCACCAAACAC
TTAATGATATCGAAGATGACTTGAGTGAGAGGAATCTCTTTGTCGACTTTGAACTTAGTAGTGATGGTGAAATTC
TCCAGCAGCTCCCAGCTGAGAAAACGTTTGCTTCGTGGTGGAATCATCAATTGAGTAGAGGATTCACAATCCCGC
ACTACAGAACAGAAGGAAAGTTTATGACCTTCACCAGGGCAACCGCCACAGAAGTTGCGGGTAAGATAGCACATG
AGAGTGATAGAGATATACTCCTCATGGGAGCGGTGGGATCAGGCAAGTCAACTGGTTTACCATATCACCTTTCCA
GAAAGGGGAATGTATTGCTTCTCGAACCCACTCGACCACTCGCAGAAAACGTGCACAAGCAATTGTCGCAGGCAC
CATTTCATCAGAACACGACTCTTCGAATGCGTGGGTTGACATCGTTTGGATCGGCACCGATCTCGGTGATGACTA
GTGGTTTCGCACTTAACTATTTCGCAAACAATCGAACGAGGATTGAAGAGTTTGACTTTGTCATATTCGATGAAT
GTCATGTTCACGACGCTAATGCAATGGCGATGAGATGTTTGCTACATGAGTGCGACTACTCCGGCAAGGTCATAA
AGGTTTCCGCCACACCACCAGGTCGCGAGGTTGAATTCTCCACTCAATATCCTGTGACAATTAGCACAGAAGACA
CATTGTCATTTCAAGACTTTGTGAATGCACAGGGTAGTGGAAGTAACTGCGACGTGATCTCAAAAGGAGACAACA
TCCTCGTATACGTAGCAAGCTACAACGAGGTGGACACACTTTCAAAGCTTCTAGTCGAGCGTGATTTCAAAGTTA
CGAAAGTTGACGGAAGAACAATGAAAGTCGGAAATATTGAAATCACCACGAGCGGGACACCTAGTAGGAAACACT
TCATAGTCGCAACCAACATTATTGAGAACGGGGTCACCCTGGATATTGATGTTGTTGCCGACTTTGGAACAAAAG
TACTCCCGTATCTTGACACGGATAATAGAATGCTTAGTACAACGAAAACAAGCATTAACTATGGGGAGCGAATCC
AAAGGCTAGGGAGAGTTGGAAGACACAAGCCAGGCCACGCTCTGAGAATTGGCCACACGGAGAAAGGGCTGAGTG
AAGTTCCAAGCTGCATTGCAACAGAAGCAGCCCTAAAGTGTTTTACGTATGGACTTCCAGTTATAACCAACAACG
TGTCTACAAGCATTCTAGGCAATGTAACGGTGAAACAGGCACGAACAATGTCTGTGTTTGAAATAACACCGTTCT
ACACAAGCCAAGTGGTTAGATATGATGGCTCGATGCATCCTCAGGTCCATGCGCTTTTGAAGAGGTTCAAGCTCA
GAGATTCTGAAATTGTCTTGAACAAATTAGCCATACCTAACCGGGGAGTAAATGCTTGGCTCACAGCTAGTGAGT
ATGCACGACTTGGTGCAAGCGTTGAGGACAGGCGTGACGTACGGATCCCTTTCATGTGTCGAGACATCCCAGAGA
AACTCCATCTAGAGATGTGGGATGTGATTGTCAAGTTCAAAGGCGATGCAGGTTTCGGGCGACTTTCAAGCGCCA
GCGCAAGCAAGGTAGCTTACACCTTGCAAACAGACGTCAACTCCATCCAGCGAACAGTCACTATTATAGATACAC
TAATCGCTGAGGAGAGAAGGAAGCAGGAGTACTTCAAAACAGTAACCTCCAACTGTGTCTCTTCTTCAAACTTCT
CACTACAGAGCATCACAAATGCCATAAAATCTCGTATAATGAAAGATCACACGTGCGAGAATATATCAGTGCTTG
AAGGAGCAAAGTCACAGTTACTCGAGTTCAGAAACTTAAACGCTGATCACTCGTTCACTACTAAGTCTGACGGGA
TTTCTCGCCATTTTATGAGTGAATATGGAGCACTCGAGGCAGTTCACCACCAAAACACCAACGACATGAGCAAGT
TCTTAAAGCTGAAGGGCAAATGGAACAAAACGCTTATCACGCGTGATGTGTTAGTGATTTGTGGAGTTCTTGGAG
GTGGAATATGGATGATCATCCAACACCTGCGTGCGAAGATCTCCGAACCCGTGACCCACGAGGCAAAAGGCAAGA
GGCAGAGACAGAAGTTGAAGTTTCGCAATGCCCGCGACAACAAAATGGGTAGGGAGGTTTACGGAGAAGACGACG
TCATTGAACACTTCTTTGGAGATGCCTACACAAAGAAGGGAAAGAGTAAAGGCAGAACTCGTGGCCTCGGCCATA
AAAACAGGAAATTCATCAATATGTATGGATTCGATCCTGAAGACTTCTCTGCAGTTAGATTCGTAGATCCACTCA
CTGGGGCAACGATAGATGAAAGTCCAATCATGGACATAGCCCTTGTGCAGGAACATTTTGGAAAAATACGGATGA
ATTTACTTGGAGAAGACGAACTGGAACCCGATGAGTTGCGAATGAATAAGACGATTCAGGCTTACTACATGAATA
ACAAAACAGGTAAAGCCCTGAAAGTGGATTTGACACCACACATACCACTAAAGGTATGTGACCTTCATGCAACAA
TTGCTGGTTTCCCGGAACGAGAACACGAACTGAGGCAAACTGGAAAAGCTCAGCCTATAGACTTAAGTGAAGTAC
CTAAAGCTAACACTGAGCTAATTCCAGTTGACCATGAAAGTAGCTCCATGTTTAGAGGGTTGCGCGACTACAACC
CAATATCCAATAACATTTGTCATCTTACAAATGTGTCAGATGGAGCATCAAATTCGTTGTATGGGGTTGGTTTTG
GACCACTCATATTAACGAACCGGCATCTCTTTGAAAGGAATAATGGTGAACTCGTGATAAAGTCAAGACATGGTG
AGTTCGTGATTAAGAACACGACTCAGCTACACTTGCTACCGATTCCGGACAGGGACCTTCTGCTAATCCGACTAC
CTAAAGACATTCCACCCTTCCCGCAAAAACTGGGATTTAGACAACCTGAGAGGGGTGAGAGGATCTGTATGGTAG
GTTCCAACTTTCAAACAAAGAGCATCACGAGTGTAGTTTCTGAGACTAGCACAATAATGCCAGTGGAGAATAGCC
AATTTTGGAAGCACTGGATTAGCACCAAGGATGGCCAATGTGGAAGCCCGATGGTGAGCACAAAGGATGGAAAAA
TACTTGGGCTGCACAGCCTGGCAAATTTCCAAAATTCCATCAACTACTTTGCTGCCTTCCCAGATGATTTTGCTG
AGAAGTACCTCTACACTATTGAGGCACACGAGTGGGTCAAGCATTGGAAGTATAACACCAGCGCAATTAGTTGGG
GCTCCCTAAATATACAAGCATCGCAGCCAGCGGGCTTGTTTAAAGTGAGCAAACTAATATCAGACCTTGACAGCA
CAGCGGTTTATGCGCAAACACAGCAGAATCGCTGGATGTATGAACAGCTGAATGGGAACTTAAAGGCAATAGCAC
ACTGCCCTAGTCAACTTGTGACGAAGCACACGGTTAAAGGGAAGTGCCAGATGTTCGACTTGTACCTCAAGTTGC
ATGATGAAGCGCGAGAATACTTCCAGCCAATGTTAGGGCAGTACCAGAAGAGTAGACTCAATCGAGAGGCATACG
CAAAGGATCTTCTCAAATATGCAACGCCAATCGAAGCGGGAAATATTGATTGTGATCTGTTTGAAAAGACAGTTG
AGACAGTCATATCAGACTTGCGAGGTTATGGTTTTGAGACGTGTAATTACGTCACTGATGAGATCGACGTATTCG
AAGCCCTGAATATGAAATCCGCAGTCGGAGCATTGTATAAAGGCAAGAAGAAAGATTATTTTGCTGAGTTCACGC
CCGAGATGAAAGAAGAGATACTCAAACAGAGTTGTGAACGCCTCTTCCTTGGGAGGATGGGAGTCTGGAATGGCT
CACTAAAGGCAGAATTGCGACCACTAGAGAAAGTGGAAGCAAACAAAACACGGACGTTTACAGCCGCGCCACTAG
ATACACTGTTAGGTGGGAAAGTCTGTGTGGATGACTTCAACAATCAGTTCTATGACCACAACCTTAGAGCTCCGT
GGAGCGTAGGTATGACAAAGTTCTATTGTGGTTGGGATCGCTTGCTAGAGTCACTACCAGATGGTTGGATTTACT

GCGATGCTGATGGGTCACAGTTCGATAGTTCACTATCGCCATACTTGATTAACGCAGTGCTCAACATCCGCTTGG
AATTTATGGAAGAGTGGGACATAGGGGAAGTAATGCTAAGAAACTTGTATACTGAGATTGTGTATACCCCTATCT
CAACGCCAGATGGCACACTCGTCAAGAAGTTCAAAGGGAACAATAGCGGACAACCGTCAACAGTCGTGGACAACA
CGCTCATGGTCATATTAGCAGTCAACTACTCACTCAGGAAGAGTGGAATTCCAAATGAATTGCGTGATAGCCTCA
TCAGGTTTTTCGTCAATGGAGATGACTTGCTGCTAGGCGTACATCCAAAGTACGAGTATGTCCTTGACACTATGG
CGGATAATTTCCGTGAACTGGGCTTGAAGTATACCTTCGATTCAAGAACCAGAGAAAAAGGTGACCTTTGGTTTA
TGTCGCACCAAGGACACAAAAGAGAAGGGATCTGGATCCCCAAGCTGGAACCAGAGCGAATAGTATCGATCCTAG
AGTGGGATCGATCAAAAGAGCCATGCCACCGACTTGAGGCAATCTGCGCAGCAATGATTGAATCGTGGGGATATG
ATAAGTTGACCCATGAGATACGCAAGTTTTATGCATGGATGATCGAACAGGCTCCATATAGCTCTCTAGCACAAG
AGGGAAAAGCTCCTTACATAGCGGAAACGGCACTAAGGAAACTTTACCTTGACAAAGAACCAGCTCAAGAAGATC
TTGCTCAATACTTACAAGCAATCTTCGAAGATTACGAAGATAGTGCTGAAGTGTGTGTTTATCACCAGGCAGGAG
AGGCGCCTGAGGTGTGCGTTTACCACCAAGCAGGTGAGACGCTTGATGCCGAGCTAACAGAAGAGCAGAAGCAGG
CTGAAAAGGAGAGAAAGGAGAGAGAGAGATCAGAGAAAGAGCGAGAAGGGCAAAGACAGCTAGCGCTTAAGAAAG
GCAAAAACGCAGCACAAGAAGAGGGCGAACGTGACAATGAAGTAAACGCCGGAACTTCCGGAACTTTTAGCGTGC
CTAGACTCAAAAGTCTTACAAGCAAAATGCGTGTGCCGAAGTATGAGAAAAGAGTCGCTCTCAACCTCGATCATT
TGATCTTATACACGCCGGAGCAGACAGATTTATCCAACACGCGTTCAACGCGGAAGCAGTTTGACACTTGGTTTG
AAGGTGTCATGGCTGACTATGAGCTAACGGAGGATAAAATGCAAATAATTCTCAATGGTTTAATGGTCTGGTGCA
TTGAGAATGGAACCTCCCCGAACATAAACGGAATGTGGGTGATGATGGACGGCGATGATCAGGTGGAATTCCCGA
TCAAACCGCTCATTGACCACGCCAAACCCACATTTAGGCAGATAATGGCCCATTTCAGTGACGTAGCTGAAGCGT
ACATTGAAAAGCGCAACCAAGACCGACCATACATGCCACGATATGGTCTTCAGCGCAATTTAACCGACATGAGCT
TAGCTCGATACGCGTTTGATTTCTATGAAATGACTTCTAGAACTCCAATACGTGCGAGAGAAGCACACATCCAGA
TGAAAGCAGCAGCACTGCGTGGCGCAAATAACAATTTGTTCGGCTTGGATGGAAACGTTGGTACAACGGTAGAGA
ACACGGAGAGGCACACGACCGAGGACGTTAATCGGAACATGCATAACTTACTTGGCGTTAAGGGGTTATGAAGTT
GTATGCTGGTAGACTATAAGTATTTAAGTTTACTCGTTAGTATTCTCGCTTATGGGAAATATGTAAGTTTGTTAA
AGCAGCCAGTGTGACTTTGTCATGTGTGTTGTTGTTACTTTCTATATTTTCGCCGAACATTTTATTGGTGTTAGC
GCATGGGGTGAGGTTCGTCCTCGATTGCCTTAACATTTGATAGGATGCAAGGGACAAAAAAAAAAAAAAAAAAAA
AAAAAAAAAAAAAAAAAAAAAAAAAAAAAACGCTGAAATCACCAGTCTCTCTCTACAAATCTATCTCTCTCTATT
TTCTCCATAAATAATGTGTGAGTAGTTTCCCGATAAGGGAAATTAGGGTTCTTATAGGGTTTCGCTCATGTGTTG
AGCATATAAGAAACCCTTAGTATGTATTTGTATTTGTAAAATACTTCTATCAATAAAATTTCTAATTCCTAAAAC
CAAAATCCAGGGGCCCTCGACGTTCCTTGACAGGATATATTGGCGGGTAAACTAAGTCGCTGTATGTGTTTGTTT
GAGATCCTCTAGGGCATGCAAGCTGATCTGGATCTCATGTGAGCAAAAGGCCAGCAAAAGGCCAGGAACCGTAAA
AAGGCCGCGTTGCTGGCGTTTTTCCATAGGCTCCGCCCCCCTGACGAGCATCACAAAAATCGACGCTCAAGTCAG
AGGTGGCGAAACCCGACAGGACTATAAAGATACCAGGCGTTTCCCCCTGGAAGCTCCCTCGTGCGCTCTCCTGTT
CCGACCCTGCCGCTTACCGGATACCTGTCCGCCTTTCTCCCTTCGGGAAGCGTGGCGCTTTCTCATAGCTCACGC
TGTAGGTATCTCAGTTCGGTGTAGGTCGTTCGCTCCAAGCTGGGCTGTGTGCACGAACCCCCCGTTCAGCCCGAC
CGCTGCGCCTTATCCGGTAACTATCGTCTTGAGTCCAACCCGGTAAGACACGACTTATCGCCACTGGCAGCAGCC
ACTGGTAACAGGATTAGCAGAGCGAGGTATGTAGGCGGTGCTACAGAGTTCTTGAAGTGGTGGCCTAACTACGGC
TACACTAGAAGAACAGTATTTGGTATCTGCGCTCTGCTGAAGCCAGTTACCTTCGGAAGAAGAGTTGGTAGCTCT
TGATCCGGCAAACAAACCACCGCTGGTAGCGGTGGTTTTTTTGTTTGCAAGCAGCAGATTACGCGCAGAAAAAAA
GGATCTCAAGAAGATCCTTTGATCTTTTCTACGGGGTCTGACGCTCAGTGGAACGAAAACTCACGTTAAGGGATT
TTGGTCATGAGATTATCAAAAAGGATCTTCACCTAGATCCTTTTAAATTAAAAATGAAGTTTTAAATCAATCTAA
AGTATATATGTGTAACATTGGTCTAGTGATTAGAAAAACTCATCGAGCATCAAATGAAACTGCAATTTATTCATA
TCAGGATTATCAATACCATATTTTTGAAAAAGCCGTTTCTGTAATGAAGGAGAAAACTCACCGAGGCAGTTCCAT
AGGATGGCAAGATCCTGGTATCGGTCTGCGATTCCGACTCGTCCAACATCAATACAACCTATTAATTTCCCCTCG
TCAAAAATAAGGTTATCAAGTGAGAAATCACCATGAGTGACGACTGAATCCGGTGAGAATGGCAAAAGTTTATGC
ATTTCTTTCCAGACTTGTTCAACAGGCCAGCCATTACGCTCGTCATCAAAATCACTCGCATCAACCAAACCGTTA
TTCATTCGTGATTGCGCCTGAGCAAGACGAAATACGCGATCGCTGTTAAAAGGACAATTACAAACAGGAATCGAA
TGCAACCGGCGCAGGAACACTGCCAGCGCATCAACAATATTTTCACCTGAATCAGGATATTCTTCTAATACCTGG
AATGCTGTTTTCCCTGGGATCGCAGTGGTGAGTAACCATGCATCATCAGGAGTACGGATAAAATGCTTGATGGTC
GGAAGAGGCATAAATTCCGTCAGCCAGTTTAGTCTGACCATCTCATCTGTAACAACATTGGCAACGCTACCTTTG
CCATGTTTCAGAAACAACTCTGGCGCATCGGGCTTCCCATACAATCGGTAGATTGTCGCACCTGATTGCCCGACA
TTATCGCGAGCCCATTTATACCCATATAAATCAGCATCCATGTTGGAATTTAATCGCGGCCTTGAGCAAGACGTT
TCCCGTTGAATATGGCTCATAACACCCCTTGTATTACTGTTTATGTAAGCAGACAGTTTTATTGTTCATGATGAT
ATATTTTTATCTTGTGCAATGTAACATCAGAGATTTTGAGACACAACGTGGCTTTGTTGAATAAATCGAACTTTT
GCTGAGTTGAAGGATCAGATCACGCATCTTCCCGACAACGCAGACCGTTCCGTGGCAAAGCAAAAGTTCAAAATC
ACCAACTGGTCCACCTACAACAAAGCTCTCATCAACCGTGGCTCCCTCACTTTCTGGCTGGATGATGGGGCGATT
CAGGCGATCCCCATCCAACAGCCCGCCGTCGAGCGGGCTTTTTTATCCCCGGAAGCCTGTGGATAGAGGGTAGTT
ATCCACGTGAAACCGCTAATGCCCCGCAAAGCCTTGATTCACGGGGCTTTCCGGCCCGCTCCAAAAACTATCCAC
GTGAAATCGCTAATCAGGGTACGTGAAATCGCTAATCGGAGTACGTGAAATCGCTAATAAGGTCACGTGAAATCG
CTAATCAAAAAGGCACGTGAGAACGCTAATAGCCCTTTCAGATCAACAGCTTGCAAACACCCCTCGCTCCGGCAA
GTAGTTACAGCAAGTAGTATGTTCAATTAGCTTTTCAATTATGAATATATATATCAATTATTGGTCGCCCTTGGC

TTGTGGACAATGCGCTACGCGCACCGGCTCCGCCCGTGGACAACCGCAAGCGGTTGCCCACCGTCGAGCGCCTTT
GCCCACAACCCGGCGGCCGGCCGCAACAGATCGTTTTATAAATTTTTTTTTTGAAAAAGAAAAAGCCCGAAAGG
CGGCAACCTCTCGGGCTTCTGGATTTCCGATCCCCGGAATTAGATCC**GTTTAAACTACGTA**AGATCGATCT**TGGC**
AGGATATATTGTGGTGTAAACGTTCCTGCGGCGGTCGAGATGGATCT**TGGCAGGATATATTGTGGTGTAAAC**GTT
CCT

TuMV-JPN1 in bold. Mutation DAG-**DAE** to eliminate aphid transmission in red. **KasI** cloning site on yellow background flanked with an artificial **NIaPro** cleavage site in purple (**silent mutations** underlined). Inserted cDNAs must regenerate an G nucleotide at the 3' side deleted from the artificial NIaPro proteolytic site to accommodate the KasI cloning site. **Plasmid backbone** in blue. Sites **NotI** and **ApaI** on yellow background. CaMV 35S **promoter** (transcription start yellow background) and **terminator** (processing and polyadenylation site underlined) in red and fuchsia, respectively. **pUC replication origin** on gray background. Marcador de selección ***Kanamycin*** selection marker (complementary) on dark gray background with a **silent mutation**. **pSa replication origin** on gray background.T-DNA **RB** with **overdrive** (underlined) on yellow background and **double LB** on read background.

>pGTuMVJPN1-crRNA-3
Insert between positions 9077 and 9082 of pGTuMVJPN1-K
GTAATTTCTACTAAGTGTAGATGCAAGAAGACCAGGCTCGAAAGCG

>pGLMV-X2 (13 374 bp)
GCGGCCGCGATTCCATTGCCCAGCTATCTGTCACTTTATTGTGAAGATAGTGGAAAAGGAAGGTGGCTCCTACAA
ATGCCATCATTGCGATAAAGGAAAGGCCATCGTTGAAGATGCCTCTGCCGACAGTGGTCCCAAAGATGGACCCCC
ACCCACGAGGAGCATCGTGGAAAAAGAAGACGTTCCAACCACGTCTTCAAAGCAAGTGGATTGATGTGATATCTC
CACTGACGTAAGGGATGACGCACAATCCCACTATCCTTCGCAAGACCCTTCCTCTATATAAGGAAGTTCATTTCA
TTTGGAGAGGAAAATAAAACAACCCAACACAACTCAAGAAAATTCATACAAACAAACAAATTCGTATTTTCAAGC
AATTCACTTTCAAGCAATTACAACATTTTCAATCACAAATGGCAACTCTAGATAACTGCACTCAAGTACACCACA
TGTTCGCCTACAATCGTGAACACGGAACGAACTACACGAGAAACCATTTCAGAAGATACTTAGCAGCCCAGCGAA
TAGGTTTCTATTACGATTGGGACGATGATGTCTATGAATGTCCAACGTGTGAAGCTATATACCATTCTTTGGACG
AAATAAAGAACTGGCACGAGTGCGATCCACCAGCATTCGATCTTAACGATTTCATAACTGATGCTAGGCTGAAAT
CAGCACCAGTTCCAGATCTCGGACCGGTGATCATCGAAACCCCACAAGCGGAGGAAAAACAAGAGCTTAATTTCT
TCGCTGCAACCCCGGCACCTGAAGTTTCACAATGGAAATGTAGAGGGTTGCAATTTGGTTCATTTACTGAACTTG
AAACATCTGAACCAGTTGTTTCGGTACCAGAACCCAAGTGTGAAGAGCCGGTTAGGACCATCGCAAAGCCAGAAG
AGTCAATCGAACAAGAGACCTGTGGAGACGGAAAAAGACTTCTTCAGGCGCAGATGGAAGTCGACAAAGCCGAAC
AAGATCTTGCATTCGCTTGCCTGAACGTTAGCCTAAAACCTAGGTTGGAGGGCAGAACTACTGCAACTATTGCAA
GACGCAGGGATGGATGTTTGGTTTATAAAACCAAATCAAGCTGGTCGCAGAGGAAGAGAACAAAGAAGATCCTTA
AGGGTGATACCCTAGCTTGTAAAAACCCATACACTCCAGCGGTTGTTGACAAAATCTCAATCGCAGGAGGCTCAT
CAGCCAGTGTGATGCACGAACAACAGAAACCTAAAATTCTGCACACTACACCATCAAGAAAGGTGGCCACGCATT
ATAAACGGACGGTGATGAACCAACAGACACTCGCAGCTCTTATAGATCAAGTTGGCACGATTCTACTAAACGCAG
GAAAAGAGTTTGAAGTGGTGGGCCGCAGGAAGCAGAAAGTAACTGGCAAGGGAACTAGACATAACGGTGTGAGGC
TGGTAAAGCTTAAAACTGCACATGAGGAAGGGCATCGGAGAAGAGTAGACATACGCATCCCCAATAGCCTGCGCT
CAATTGTTATGCGTATTTCAGCTCGGGGCGGTTGGCACAGAACATGGAAAGATTCAGAACTATCCCCAGGATCAA
GCGGTTACGTATTGAACTCGTCAAAAATCATAGGAAAATTTGGTTTAAGGCGACACAGCATATTCATTGTTAGGG
GGAGGGTAGATGGTGAAGTTATAGACTCGCAAAGTAAGGTCACACATTCCATCACGCACCGTATGGTTCAATACA
GCGACGTCGCACGAAACTTCTGGAACGGGTACTCAACTTGTTTCATGCACAATACCCCAAAGGACATACTCCATA
CCTGCACATCAGATTTTGATGTTAAAGAATGTGGCACTGTCGCAGCACTTTTAACTCAAACACTGTTTCAATTTG
GGAAAATCACCTGTGAAAAGTGCGCAATCGAGTACAAAAATCTAACGCGAGACGAGCTCGCTACGCGTGTTAATA
AGGAGATTGATGGAACCATAATCAGCATTCAAACTCAGCATCCACGCTTCGTGCATGTACTCAATTTTCTCAGGT
TAATTAAACAAGTACTCAATGCTAAGAATGAGAACTTTGGAGCATTTCAAGAGACGGAGAGGATAATTGGGGATC
GAATGGATGCACCTTTCTCACACGTAAATAAGCTGAATGCTATCGTCATTAAAGGTAATCAAGCAACATCTGATG
AGATGGCACAAGCATCGAACCATGTCCTCGAAATCGCACGATATCTCAAGAACCGAACTGAGAACATCCAAAAGG
GCTCACTAAAGTCATTCAGGAACAAAATCTCCGGTAAGGCACACTTAAATCCGAGTCTTATGTGTGACAATCAAC
TTGATAAGAACGGCGGGTTTGAATGGGGGCAGCGAAGTTACCATGCTAAAAGGTTTTTCGACGGATACTTCGAAA
CCATTGACCCATCTGATGGCTATAGCAAATACACCATAAGACGCAATCCAAATGGACATCGAAAGTTGGCAATTG
GTAATTTGATCGTCTCCACGAACTTTGAATCACATAGAAGAAGCATGATTGGAGAATCAATCGAAGACCCTGGTC
TCACTAACCAGTGCGTGAGCAAAGAGGGAGATGCCTTCATCTATCCATGCTGCTGTGTAACAGATGAATATGGTA
AACCAACATTATCTGAGATTAAAATGCCTACAAAGCATCATCTAGTCCTAGGAAATGCCGGTGACCCCAAATATG
TGGATTTACCAAAGGAAGCGGAAGGAAAGATGTTCGTAGCAAAAGACGGATATTGTTACATAAACATCTTCTTGG

CTATGCTTGTTGACGTCCCAGAGGATCAAGCTAAAGATTTCACGAAGATGGCACGCGAGATAGCAGTGAAACAGC
TCGGGGAGTGGCCCTCAATGATGGATGTAGCAACGGCTTGTAATATATTAGCTACATTTCATCCAGACACTCGAA
GATCGGAGTTACCTCGAATCTTAGTCGACCACGCAACGAAAACATTCCATGTAATTGATTCATATGGTTCAATCA
CGACTGGATTCCATATTCTGAAAGCCAACACCGTGACGCAACTCGTCAAGTTCGCGCATGAGTCACTAGAATCTG
AGATGCAACACTACAGAGTAGGGGGGGAACCAGATAAAGCACCCAGGAAACCAGCTGGCAGTGTCCCAACTCTAG
GAATTTCAGACCTCAGGAACCTTGGAGTGGAATCAGAAAACGAAGAGCACTCAATCCGGCCAAATCTCCAAAGGT
TGATCAAGGCGATTTACAGACCCCGAATGATGCGCAGCCTCTTAACAGAGGAACCATACTTGTTAATCCTAAGTA
TTGTGTCCCCTGGCGTTCTGATGGCACTCTACAACAGTGGTTCCTTGGAGCGGACAATGCATGAATTCTTGCAAA
CTGACCAGAGACTGAGCGCCACCGCCCAAATCCTAAAACATCTAGCAAAGAAAGTTTCACTCGCAAAGACACTTA
CGATTCAGAATGCTATCTTGGAGGGTGGAGCAGGATCACTGAATGAAATTCTTGACGCGCCCGCGGGACGATCCC
TATCGTATAGACTAGCAAAGCAGACGGTGGAGGTGATGATGGCGCGGAGCGACATGGATAAGGAACTGGTGGACG
TTGGATTTAGCGTTCTTAGGGATCAGAAGAATGAACTCATAGAAAAAAGTTATCTCATGGATTTGGAGGACTCGT
GGCACGCACTACCATTGTGTGGAAAATTATCGGCAATGCGAGCCTTGCGGCGATGGCGGGACACCTCTACTCCCG
AAGCAATCCCGACAGGTGCCGCAGATTTGAAAGGCAGATACAGTATCTCGGTTGGATCTGTTTCCAAAAGCGCGA
TCTTACACCTAAAGGGAATTTGTTCAGGCGCAGTAAAGAGAGTTAGAGACAAGTGGGTCGGAGTGCAAGTGCAAG
GCGTAAAATGGTTAGCTAAATCAGTACACTACATGATACCAGAACTAATGAACGTACTGAATGTTGGAACTCTCC
TCCTAACGCTGATATCGCTAGGCGTAGCATTCCGGAATTTGACTGGCCAGTTCAAAGAGATGAAACACAAAGAAA
CGCTGGCAAAGGAGGAGGAACTACGTAAGCGCATACGCACCTACAATAGCACTTACTACGAAATCCATGGAAAGC
ACGCTGATGCCAAACAAATCACTAAATTCATAACACACCATGATCCAAAACTACTGGAAGTAGTTGAGTTTTACG
AAGGTCCTGAAGAAGAAGAAGTGGAACATCAAGCAAAGCGAGAGGATCAAGCTAATCTCGAACGCATTATCGCTT
TTACAGCCTTAGTGATGATGATGTTTGACAGCGAGAGAAGTGATTGTGTGTACAGGAGTCTATCAAAGCTTAAAT
CATTAGTATCAACGTGTGAGGATAATGTACGCCACCAGAGCGTTGATGAGATCATTGACCTGTTCGACGAGAAGA
AAGAAACTATAGATTTCGAGATTGAAGGAAAGGAACTTTACTCTTCTCGCGTGGTGGATTCGACATTCAGCAAGT
GGTGGGACAATCAATTGGCACGGGGCAACACGATGGCACATTACAGAACAGAGGGACACTTCATGACGTTCACTC
GTGAAACAGCTGCGAGCGTGGCAGCCGAAATAGCACATAACGAGTATAGAGATATTCTCTTGCAAGGTGGTGTCG
GCTCCGGCAAGTCTACAGGTCTCCCGTTCCATTTGCACAAGAAGGGAGGTGTACTACTCATCGAACCAACTCGTC
CGTTAGCTCAAAATGTATACAAGCAGCTTGGAAGTAGCCCTTTTCACCTGTCACCAAATTTGCGTATGCGAGGTT
CTTGCAAGTTTGGATCTAGTCAGGTGACTGTGTCCACGAGTGGTTATGCCTTACATTTCATAGCGAACAATGCGC
AAAGTCTTAAGGCATATGACTTCATCATTTTTGATGAATGTCACGTATTAGATGCCAGTGCAATGGCGTTTAGAT
GCTTACTGCAGGAGTTCGAGTATCAAGGGAAGATCATAAAGGTATCAGCCACGCCACCGGGAAGAAAGCTTGACT
TCAAACCAATGCACATGGTCGATATTGCTACAGAAAATGAACTATCGATACAGCAATTCGTCCAAGGTCAAGGAA
CTGGAGTAAACTGTGATGCAACAAAGAAAGGAGACAATATCTTGGTCTACGTCTCAAGTTACAACGAAGTGGACA
TGTTGAGCAAAATGTTAAACGACAAAGGTTACAAAGTGACAAAGGTTGACGGTAGGACGATGAAGTTAGGGAGTG
TGGAAGTAGAAACAGTAGGCACCCCACAGCGGAAACACTTTGTAGTGGCAACCAACATCATAGAAAATGGCGTTA
CATTGGATGTCGATGTCGTGGTGGATTTCGGGCAGAAGGTAGTTCCCATTCTTGACAGCGAGCACCGAATGATCC
GGTATACAAAGAAAAGTATCACATATGGTGAGCGAATCCAAAGAGTGGGAAGGGTCGGGCGAAACAAAGCTGGTT
CTGCCATCCGGATAGGGAGCACTGAGATGGGGACGGAAGAAATACCGGCATCAATCGCAACAGAAGCAGCCTTTT
TGTGTTTCACATACGGATTTCCTGTCATGACAAGCAACGTGAGCACAAGTGTACTTGGCAATTGCACAGTTAGAC
AAGCACGGACGATGCAAAAGTTTGAGCTGTCCCCCTTCTTTATGGTTGACTTGGTACACCACGACGGTACAATGC
ACCCAGCAATTAACAGCCTCTTGAAACAGTTCAAATTGAAGGAATCAGACATCACACTAAGCACGCTGGCAATAC
CGAATGCAGTAACCACATTCTGGAAAAGCGCTCGAGAGTATAACTCCTTAGGTGCTCGCACAACAATCGATGACG
CAGCTAAAATACCATTTATGATCAAGGATGTTCCAGAACACTTGCAGGAAAAGCTTTGGGAGACAATCCAGCAGT
ATAAGGGTGATGCAGGTTTTGGAAGATGCACATCAGCTAACGCGTGCAAAATAGCATACACACTCTCTGTGAGTC
CCTTTATGATCCCAGCAACAATCAACAAGATTGATGCTCTGATGGCCGAAGAACGACAGAAGATGGAATATTTCC
AAACAGTCACAGCTAATACATGTACAATCTCAAACTTCTCCATTAGTAGCATTGGCGACATGATCAGGTCGAGAT
ACTCAACAAACCATTCTAGAGAAAACCTGCAGAAATTACAAACGGTCAGAGACACAATCATCAACTTTGAGTGCC
AGGCTGGAACTAGTGATGGGGGTACCTTCGACATGGAGACAGCACAGAAATTGGCAGAGGAGTATGGGTGCATCG
ATGTCATTTATCACCAATCGAAAGGAGCTCTTAGCAAACGACTCGGTCTCAAAGGCAGGTGGAATCAGAGCCTTA
TATGCAAAGACCTATTGATTTTCTGCGGCGTAGCCATTGGCGGCACATGGATGATGTTTCAGAGCTTCAAGGACG
GGATGGCTGACGTAATTCGACACCAAGGCAAAGGTAAACGGCAAAGACAGAAGCTTCGTTATCGACAAGCAAGGG
ATAATAAGATGGGCATTGAAGTTTATGGTGACGATGCGACGATGGAACACTATTTTGGAGCTGCGTACACAGAGA
AAGGAAAGAAATCCGGAAAGACGAAAGGAATGGGGACGAAAAATCGAAGATTTGTTAACATGTATGGGTACAACC
CAGAAGATTACTCGTTCATCCGATTTCTGGACCCACTCACAGGGAAAACAATGGATGAACAAGTATTCACTGACA
TAAGTCTCGTCCAAGATGCCTTTGGTAAAGAAAGACTCAAACTCCTGTCCGAAGGGGAAATTGAGTCAGAGCACA
TGCGAAATGGGATTAGAGCTTATCTTGTCAAGAATCTTACCACAGCAGCTCTCGAAATAGACATGACCCCTCACA
ACTCTTGCCAGCTCGGAACCAAGACAAACAACATAGCAGGATTTGTAGACAGGGAGTACGAATTGCGTCAAACCG
GGGAAGCCAGGGTTGTTGCTCCAGCACTGATTCCAAAAGACAATCCAATCACGGACGAGGATATTCCCGTAAAGC
ATGAAAGCAAGACATTGTTCAGGGGCTTAAGAGATTATAACCCTATAGCATCAGCAATATGCTTGCTCACTAACG
AATCAGACGGAATGAAAGAGACAATGTACGGCATTGGTTTTGGCAACACAATTATTACAAACCAACACCTATTCA
GACGCAACAATGGCGTACTAAGGGTTCAGTCGAGACATGGTGAATACGTTCTTCCAAATACAACGCAACTCAAAG
TACTTCCTTGCGAAGGAAGGGACATAATGGTCATCATTCTTACACCAGACTTCCCTCCGTTCCCACAAAAACTGA

AATTCCGTCCACCGATCAAAGGAGAGAAGATTTGCCTTGTTGGATCCTTATTTCAAGATAAGAGCATAACCAGCA
CCGTATCCGAGACGAGCGTGACAACACCCGTGGACAACAGCTTCTTGTGGAAACACTGGATCACTACAAAAGACG
GACATTGCGGGCTTCCACTAGTATCATCAAATGATGGATACATAGTCGGAATCCACAGCGCCACAAGCTCACGGC
AAACACAGAACTACCATGCAGCGATGCCCGAAGATTTTCATCAAACACATCTTATTGATCCAGTTTCGAAATCGT
GGGTGAAACATTGGAAGTATAATCCAGATAACATGGTTTGGGGAGGCATAAATCTCATTAATAGCACGCCAAGGG
AACCCTTCAAGATAAACAAATTAGTGACAGACTTATTCGGGGATGCAGTACAGTTTCAGTCCAAACAGGATGAGT
GGTTCGCAAGTCAGTTGAAAGGCAACTTGAAAGCAGTGGGGAAAAGCACAAGCCAACTCGTGACAAAGCACACAG
TCAAGGGTAAGTGCATGATGTTCGAGTTATACCTGCAAACACACGAAGAGGAAAAGGAATTCTTCAAACCACTGA
TGGGAGCCTACCAGAAGAGCCGCTTAAACAGAGAAGCATTTACAAAAGACATCATGAAATACTCCACACCGATAA
CAGTAGGCATCGTCGATTGTGACACGTTTCTGAAAGCCGAAAAAGGAGTCATAAAACGCTTAGAGAAACTTGGGT
TCAGTGGTTGCGAATATGTCACAGATGAAGAGGCAATATTTCAAGCCCTAAATATGAAGGCGGCTGTTGGTGCAC
TATATAGTGGAAAGAAGAGAGATTACTTTGAGAGCTACGGTCCAGAAGAAAAAGAGAATATCTTGAGAGAAAGCT
GTAAGCGACTATACACAGGTAAGTTTGGAGTGTGGAATGGGTCACTCAAGTCGGAACTGAGACCTATGGAGAAAG
TTATGGCAAATAAGACACGTGTCTTTACAGCAGCGCCGCTCGACACTCTGCTTGCTGGAAAAGTCTGTGTTGATG
ACTTCAACAATTACTTCTACAGCAAAAACATCGAGGCACCTTGGACAGTTGGCATGACGAAGTTTTATGGTGGGT
GGAACGAACTCCTTACGAAGTTACCAGACGGCTGGGTTTATTGCGATGCAGATGGATCACAATTCGATAGTTCTT
TGTCACCATTTCTCATCAACTCAGTTCTCAGAATACGTTTAAAGTTCATGGAAGATTGGGATCTTGGTGAACAAA
TGCTCAAAAATTTGTACACAGAAATTGTATATACAGCGATCCTCACTCCAGATTCAACCATAGTGAAGAAATTCA
AGGGGAATAACAGCGGACAGCCATCCACGGTAGTAGATAACACATTGATGGTCGTGCTAGCAATGACATACACGC
TGCACAAGTTAGGCTTCGAGGACGAGGAACAAGACTCTATGTGCAAATACTTCGTCAATGGAGATGACCTGATCA
TCGCGATAAAACCAGAATATGAATCACTACTGGATCAGTTCCAACACTGTTTCAAAAGTTTGGGCTTAAATTACG
ATTTCAACTCACGAACGAGGAAGAGGGAGGAATTGTGGTTCATGTCACATTGCGGCATCAAGAAGGATGGAATCT
TCATCCCAAAACTCGAACCAGAGCGCATTGTGTCAATCCTAGAGTGGGATAGATCAGACCAGCCAGTACATCGCT
TAGAGGCCATATGCGCAGCAATGATCGAATCATGGGGTTACGACAAGTTAACCCATGAAATTCGAAAATTTTACA
AGTGGTGTCTGGACGAAGCACCATATGCTGATTTGGCAAAAGCGGGAAAAGCACCGTACATAGCAGAGTGTGCTC
TTAAACGATTGTATACCAGCAAGGAAGCCAGCGAAGCAGAATTAGAAAAATACATGGAAGCCATACGTAGTCTTG
TCAATGATGAAGATGACGACGATATGGATGAAGTCTATCATCAAGTGGACACCCGGGACATGGACGAAGTTTATC
ACCAAGTAGACACGAAGCTTGATGCAGGCCAAGGCAGTAAAAATGATGATAAACAGAAGAGCTCAGCGGATTCAA
AAGATAATGTCATCACGGAGAAAGGAAGTGGTTCTGGGCAGGTGAGGAAGGATGACGACATCAACGCAGGGCTAC
ATGGCAAACACACCATACCTCGTACAAAGGCAATCACACAGAAAATGAAGTTACCAATGATCCGAGGTAAAGTGG
CTTTGAACCTTGATCATTTGCTGGAGTACGAACCAAACCAGAGAGACATATCAAACACACGCGCGACTCAAAAAC
AATACGAGTCATGGTACGACGGAGTTAAGAATGACTATGATGTGGATGATAATGGCATGCAATTAATTCTGAACG
GATTGATGGTTTGGTGTATAGAAAACGGGACATCCCCGAATATAAATGGAACATGGGTGATGATGGACAGTGAAG
AACAAGTAGAATATGCTCTGAAACCCATCATCGAACACGCGAAACCCACGTTTCGCCAGATAATGGCCCATTTTA
GTGACGCAGCCGAGGCGTACATTGAGATGAGAAACAAGAAGAAACCGTATATGCCACGATACGGACGGCTACGAG
GCTTGAACGATATGGGGTTAGCTCGCTACGCTTTCGACTTTTACGAAACAACATCAGCGACCCCAAATCGGGCGA
GAGAGGCGCACAATCAAATGAAGGCAGCTGCTCTAGTGGGAACACAGAACAGACTGTTTGGAATGGATGGAGGCG
GTTCAACCCAGGAAGAGAACACGGAGAGGCACACAGCCGCAGATGTAAATCAGAATATGCACACTCTCTTAGGCG
TGAGAGGGTTGCACTAAAGGCGTGTGTTGGCATTTAAAGACTGTAGTATAAATCTATAATATAGTGAGTGTTTCA
CTCCTTTTATGTTTATGTATGCACTTATTTGTCCGTTAGTATTCTCTCCCTGTACTTCGCTCGTAAGGGCGTAGG
TTCAGCTGAGGGCTTTTCTAGTTTCCGCTGTGAGGTTTTACCTCGAAGGTTGCTAGTCTGGTTTTCAGTCGGAGA
CAAAAAAAAAAAAAAAAAAAAAAAAAAAAAAAAAAAAAAAAAAAAAAAAAACGCTGAAATCACCAGTCTCTCTCT
ACAAATCTATCTCTCTCTATTTTCTCCATAAATAATGTGTGAGTAGTTTCCCGATAAGGGAAATTAGGGTTCTTA
TAGGGTTTCGCTCATGTGTTGAGCATATAAGAAACCCTTAGTATGTATTTGTATTTGTAAAATACTTCTATCAAT
AAAATTTCTAATTCCTAAAACCAAAATCCAGGGGCCCTCGACGTTCCTTGACAGGATATATTGGCGGGTAAACTA
AGTCGCTGTATGTGTTTGTTTGAGATCCTCTAGGGCATGCAAGCTGATCTGGATCTCATGTGAGCAAAAGGCCAG
CAAAAGGCCAGGAACCGTAAAAAGGCCGCGTTGCTGGCGTTTTTCCATAGGCTCCGCCCCCCTGACGAGCATCAC
AAAAATCGACGCTCAAGTCAGAGGTGGCGAAACCCGACAGGACTATAAAGATACCAGGCGTTTCCCCCTGGAAGC
TCCCTCGTGCGCTCTCCTGTTCCGACCCTGCCGCTTACCGGATACCTGTCCGCCTTTCTCCCTTCGGGAAGCGTG
GCGCTTTCTCATAGCTCACGCTGTAGGTATCTCAGTTCGGTGTAGGTCGTTCGCTCCAAGCTGGGCTGTGTGCAC
GAACCCCCCGTTCAGCCCGACCGCTGCGCCTTATCCGGTAACTATCGTCTTGAGTCCAACCCGGTAAGACACGAC
TTATCGCCACTGGCAGCAGCCACTGGTAACAGGATTAGCAGAGCGAGGTATGTAGGCGGTGCTACAGAGTTCTTG
AAGTGGTGGCCTAACTACGGCTACACTAGAAGAACAGTATTTGGTATCTGCGCTCTGCTGAAGCCAGTTACCTTC
GGAAGAAGAGTTGGTAGCTCTTGATCCGGCAAACAAACCACCGCTGGTAGCGGTGGTTTTTTTGTTTGCAAGCAG
CAGATTACGCGCAGAAAAAAAGGATCTCAAGAAGATCCTTTGATCTTTTCTACGGGGTCTGACGCTCAGTGGAAC
GAAAACTCACGTTAAGGGATTTTGGTCATGAGATTATCAAAAAGGATCTTCACCTAGATCCTTTTAAATTAAAAA
TGAAGTTTTAAATCAATCTAAAGTATATATGTGTAACATTGGTCTAGTGA*TTAGAAAAACTCATCGAGCATCAAA*
TGAAACTGCAATTTATTCATATCAGGATTATCAATACCATATTTTTGAAAAAGCCGTTTCTGTAATGAAGGAGAA
AACTCACCGAGGCAGTTCCATAGGATGGCAAGATCCTGGTATCGGTCTGCGATTCCGACTCGTCCAACATCAATA
CAACCTATTAATTTCCCCTCGTCAAAAATAAGGTTATCAAGTGAGAAATCACCATGAGTGACGACTGAATCCGGT
GAGAATGGCAAAAGTTTATGCATTTCTTTCCAGACTTGTTCAACAGGCCAGCCATTACGCTCGTCATCAAAATCA

CTCGCATCAACCAAACCGTTATTCATTCGTGATTGCGCCTGAGCAAGACGAAATACGCGATCGCTGTTAAAAGGA
CAATTACAAACAGGAATCGAATGCAACCGGCGCAGGAACACTGCCAGCGCATCAACAATATTTTCACCTGAATCA
GGATATTCTTCTAATACCTGGAATGCTGTTTTCCCTGGGATCGCAGTGGTGAGTAACCATGCATCATCAGGAGTA
CGGATAAAATGCTTGATGGTCGGAAGAGGCATAAATTCCGTCAGCCAGTTTAGTCTGACCATCTCATCTGTAACA
ACATTGGCAACGCTACCTTTGCCATGTTTCAGAAACAACTCTGGCGCATCGGGCTTCCCATACAATCGGTAGATT
GTCGCACCTGATTGCCCGACATTATCGCGAGCCCATTTATACCCATATAAATCAGCATCCATGTTGGAATTTAAT
*CGCGGCCTTGAGCAAGACGTTTCCCGTTGAATATGGCTCAT*AACACCCCTTGTATTACTGTTTATGTAAGCAGAC
AGTTTTATTGTTCATGATGATATATTTTTATCTTGTGCAATGTAACATCAGAGATTTTGAGACACAACGTGGCTT
TGTTGAATAAATCGAACTTTTGCTGAGTTGAAGGATCAGATCACGCATCTTCCCGACAACGCAGACCGTTCCGTG
GCAAAGCAAAAGTTCAAAATCACCAACTGGTCCACCTACAACAAAGCTCTCATCAACCGTGGCTCCCTCACTTTC
TGGCTGGATGATGGGGCGATTCAGGCGATCCCCATCCAACAGCCCGCCGTCGAGCGGGCTTTTTTATCCCCGGAA
GCCTGTGGATAGAGGGTAGTTATCCACGTGAAACCGCTAATGCCCCGCAAAGCCTTGATTCACGGGGCTTTCCGG
CCCGCTCCAAAAACTATCCACGTGAAATCGCTAATCAGGGTACGTGAAATCGCTAATCGGAGTACGTGAAATCGC
TAATAAGGTCACGTGAAATCGCTAATCAAAAAGGCACGTGAGAACGCTAATAGCCCTTTCAGATCAACAGCTTGC
AAACACCCCTCGCTCCGGCAAGTAGTTACAGCAAGTAGTATGTTCAATTAGCTTTTCAATTATGAATATATATAT
CAATTATTGGTCGCCCTTGGCTTGTGGACAATGCGCTACGCGCACCGGCTCCGCCCGTGGACAACCGCAAGCGGT
TGCCCACCGTCGAGCGCCTTTGCCCACAACCCGGCGGCCGGCCGCAACAGATCGTTTTATAAATTTTTTTTTTTG
AAAAAGAAAAAGCCCGAAAGGCGGCAACCTCTCGGGCTTCTGGATTTCCGATCCCCGGAATTAGATCCGTTTAAA
CTACGTAAGATCGATCTTGGCAGGATATATTGTGGTGTAAACGTTCCTGCGGCGGTCGAGATGGATCTTGGCAGG
ATATATTGTGGTGTAAACGTTCCT

LMV (**XbaI** recombinant between LMV-E [X97705.1] and LMV-AF199 [AJ278854.1]) in black with a cloning site **XmaI** to insert cDNAs of interest by Gibson assembly between NIb and CP cistrons. The heterologous protein will be flanked by an artificial **NIaPro** proteolytic site (+3/-8, purple with silent mutations with respect to the native NIb/CP site underlined). Inserted cDNAs must regenerate an A nucleotide at the 5' side deleted from the artificial NIaPro proteolytic site to accommodate the XmaI cloning site. Unique restriction sites **NheI-Bpu1102I (=BlpI)** on green background. **Plasmid backbone** in blue. Sites **NotI** and **ApaI** on yellow background. CaMV 35S **promoter** (transcription start on yellow background) and **terminator** (processing and polyadenylation site underlined) in red and fuchsia, respectively. **pUC replication origin** on gray background. Marcador de selección ***Kanamycin*** selection marker (complementary) on dark gray background with a **silent mutation**. **pSa replication origin** on gray background.T-DNA **RB** with **<u>overdrive</u>** (underlined) on yellow background and **double LB** on read background.

>pGLMV-crRNA-3
Insert between positions 9352 and 9357 of pGLMV-X2
AGTAATTTCTACTAAGTGTAGATGCAAGAAGACCAGGCTCGAAAGC
